# Stochastic Modeling of Gene Expression Evolution Uncovers Tissue- and Sex-Specific Properties of Expression Evolution in the *Drosophila* Genus

**DOI:** 10.1089/cmb.2022.0121

**Published:** 2023-01-05

**Authors:** Soumitra Pal, Brian Oliver, Teresa M. Przytycka

**Affiliations:** ^1^ National Center for Biotechnology Information, National Library of Medicine, National Institutes of Health, Bethesda, Maryland, USA.; ^2^ Laboratory of Cellular and Developmental Biology, National Institute of Diabetes and Digestive and Kidney Diseases, Bethesda, Maryland, USA.

**Keywords:** adaptive evolution, expression evolution models, organ-specific gene expression, Ornstein-Uhlenbeck process, sex-specific gene expression, stabilizing selection

## Abstract

Gene expression evolution is typically modeled with the stochastic Ornstein-Uhlenbeck (OU) process. It has been suggested that the estimation of within-species variations using replicated data can increase the predictive power of such models, but this hypothesis has not been fully tested. We developed EvoGeneX, a computationally efficient implementation of the OU-based method that models within-species variation. Using extensive simulations, we show that modeling within-species variations and appropriate selection of species improve the performance of the model. Further, to facilitate a comparative analysis of expression evolution, we introduce a formal measure of evolutionary expression divergence for a group of genes using the rate and the asymptotic level of divergence. With these tools in hand, we performed the first-ever analysis of the evolution of gene expression across different body-parts, species, and sexes of the *Drosophila* genus. We observed that genes with adaptive expression evolution tend to be body-part specific, whereas the genes with constrained evolution tend to be shared across body-parts. Among the neutrally evolving gene expression patterns, gonads in both sexes have higher expression divergence relative to other tissues and the male gonads have even higher divergence than the female gonads. Among the evolutionarily constrained genes, the gonads show different divergence patterns, where the male gonads, and not the female gonads, show less constrained divergence than other body-parts. Finally, we show interesting examples of adaptive expression evolution, including adaptation of odor binding proteins.

## 1. INTRODUCTION

Studies of species evolution typically focus on the evolution of DNA sequence. However, in complex multi-cellular organisms, all cells utilize the same genetic information, yet they show remarkable phenotypic differences arising from distinct transcriptional programs executed in different organs, tissues, and cells. Although evolution ultimately acts at the level of the successful reproduction of individuals, divergent transcriptional programs within the individual are subject to evolutionary adaptation, constraint, and drift. Evolutionary analysis of gene expression can, thus, shed light on the evolutionary processes in ways that cannot be achieved by the analyses of sequence alone, and can provide a richer understanding of evolution.

Currently, our understanding of the interplay between species evolution and tissue-specific expression evolution is still limited. The results of initial analyses of gene expression evolution have not provided a clear picture of the dominant forces. For example, early studies of the evolution of primate gene expression suggested that expression evolution is largely consistent with the neutral theory of evolution (Khaitovich et al., 2004, 2005) whereas subsequent analyses uncovered signatures of positive selection in the brain (Khaitovich et al., 2006b) and testis (Khaitovich et al., 2006a).

In the past decade, several gene expression datasets encompassing a larger number of species have been collected and analyzed [mammals (Brawand et al., 2011; Romero et al., 2012; Chen et al., 2019), vertebrates (Chan et al., 2009), primates (Blekhman et al., 2008)]. Studies in mammals indicated that, when restricted to individual tissues or organs, gene expression evolution tends to follow the species tree fairly closely (Chen et al., 2019). However, multi-tissue samples generally clustered first by tissues rather than by species (or study) (Chan et al., 2009; Brawand et al., 2011; Breschi et al., 2016; Chen et al., 2019), suggesting strong evolutionary constraints on tissue-/organ-specific gene expression. However, a recent study identified sets of gene expression levels that cluster first by species (Breschi et al., 2016). Finally, sex-biased expression also imposes constraints on evolution (Meiklejohn et al., 2003). However, the nature of constraints imposed by combination of tissue and sex specificity is less clear. In addition, tissue- and sex-specific studies of gene expression adaptation are very limited.

Recently, we collected a large dataset of body-part and sex-specific gene expression focusing on the *Drosophila* phylogeny (Yang et al., 2018). We use these data along with new computational tools developed in this study, to examine modes of gene expression evolution of *Drosophila* genus in the context of body-parts, sex, and species groups.

Following the pioneering work of Felsenstein (1973), neutral evolution of continuous traits, such as gene expression, is formally modeled by Brownian Motion (BM). Along a similar line of thought, Lande (1976) pioneered the use of the Ornstein-Uhlenbeck (OU) process to model evolutionary constraint and adaptive evolution of continuous traits. The OU process is stochastic and extends the BM model by adding an “attraction force” toward an optimum value. Combining the OU process on gene expression with the information about the underlining evolutionary tree (obtained, e.g., from sequence analysis) allows modeling the differences in evolution along individual branches and, thus, helps uncover branch-specific adaptation (Hansen, 1997; Butler and King, 2004; Brawand et al., 2011; Chen et al., 2019).

Phylogenetically diverse gene expression datasets that span many organisms across the tree of life and include expression data on multiple tissues/organs have challenged us to put these theoretical models into practical use. For example, several recent works used stochastic models to study evolution of gene expression (Bedford and Hartl, 2009; Kalinka et al., 2010; Brawand et al., 2011; Nourmohammad et al., 2017; Chen et al., 2019). Unfortunately, these formal stochastic models for continuously varying phenotypic traits usually do not account for within-species variation. It has been long suggested that ignoring within-species variation might bias the results in comparative data analysis (Ives et al., 2007; Felsenstein, 2008; Rohlfs et al., 2014).

In line with this assumption, within-species variation was considered in the initial analysis of mammalian gene expression (Brawand et al., 2011). However, early simulations failed to confirm the expectation that within-species variations help in differentiating between neutral and constrained evolution (Rohlfs et al., 2014). Therefore, providing a better understanding of the benefits and limitation of both models is of paramount importance.

Our contributions here are threefold. First, given the earlier conflicting results and having in mind the need to facilitate future analyses of gene expression evolution, we developed EvoGeneX, a computationally efficient implementation of the OU process-based model of gene expression evolution for a given set of species under the assumption that for each species, expression data from several individual organisms are available. Using detailed simulations, we demonstrated the advantages and limitations of such an extended model.

Specifically, we show that by modeling the within-species diversity EvoGeneX improves over the model that averages replicates implemented by program OUCH (King and Butler, 2009) that is currently accepted as the basic framework for gene expression evolution (Chen et al., 2019). Further, previous studies suggested that the performance of the OU-based methods can depend on the size of the evolutionary tree (Rohlfs et al., 2014; Catalán et al., 2019), suggesting, in particular, that the OU model can require a tree of at least 50 nodes (Catalán et al., 2019).

However, these analyses have not separated the impact of the number of nodes from the influence of the evolutionary distances to the root. Our extensive simulations provide a window on this question clarifying previous misunderstandings. These results demonstrate that selecting the species with larger evolutionary distances is more important than the number of selected species. At the same time, thanks to our efficient Maximum Likelihood estimation, EvoGeneX is very efficient, despite the increased size of data and the number of parameters to be optimized.

Next, to facilitate comparative analysis of the dynamics of gene expression evolution between organs and sexes, we introduce a formal approach based on Michaelis-Menten kinetics (Michaelis and Menten, 1913) from the enzyme kinetics theory, to measure the dynamics of evolutionary divergence of a group of genes in terms of the group's asymptotic divergence level and rate to reach the asymptotic level.

Finally, we used EvoGeneX, along with other methods we developed in this study, to perform the first ever analysis of organ- and sex-specific gene expression evolution in *Drosophila*. Specifically, we applied EvoGeneX to provide a comparative analysis of gene expression evolution based on a dataset encompassing five different body-parts (head, gonads, thorax, viscera, and abdomen), from carefully selected representatives of the *Drosophila* genus (both male and female) collected for the purpose of this study.

For each of the 10 total sample types, gene expression was measured by RNA-seq in 4 biological replicates (Yang et al., 2018). The genus *Drosophila* is particularly well suited for studying gene expression evolution. The last common ancestor of the genus is assumed to date to the Cretaceous period about 112 ± 28 million years ago (Wheat and Wahlberg, 2013). The *Drosophila* species occupy diverse geographic locations and ecological niches (Morales-Hojas and Vieira, 2012). Compared with the previous studies in mammals (Brawand et al., 2011; Chen et al., 2019) and vertebrates (Chan et al., 2009), the morphology of the *Drosophila* species is similar while at the same time the evolutionary distances are significantly larger relative to mammals or even vertebrates typically included in such studies. This makes *Drosophila* an ideal phylogeny to study the interplay among different modes of gene expression evolution.

Our analysis demonstrated that, in *Drosophila*, constrained evolution of gene expression is more common than neutral evolution; however, neutral evolution of gene expression cannot be rejected in a very large fraction of the genes. We found that the expression of many genes is evolutionary constrained in multiple body-parts. However, not all genes follow this pattern and have different evolutionary trends depending on where they are expressed. To gain further understanding of variations in evolutionary dynamics between organs and sexes, our Michaelis-Menten kinetics-based approach revealed striking differences in evolutionary dynamics of gene expression in male and female gonads, for example.

We also provide analysis of adaptive expression evolution at the subgenus level. We show that although the property of constrained evolution of the expression of specific genes is often shared across many organs, in contrast, adaptive evolution tends to be body-part specific.

Previous studies in mammals (Chen et al., 2019) observed that gene expression evolution in testis is least constrained among the organs analyzed by these studies. However, no analysis of female gonads has been performed earlier. We found that, among the genes with neutrally evolving gene expression, gonads in general evolve the fastest, whereas male gonads evolve even faster than female gonads. In addition, we show that male and female gonads also have the largest sets of unique adaptive gene expression levels.

Finally, EvoGeneX revealed compelling and interesting examples of adaptive evolution of gene expression in the *Sophophora*/*Drosophila* branches of the phylogeny.

## 2. RESULTS AND DISCUSSION

### 2.1. Formal description of OU-based gene expression models and high-level overview of EvoGeneX

Given a gene with one-to-one orthology relation across a set of species, the species tree, and expression values of the gene measured in multiple biological replicates for each species, our goal is to uncover the most likely scenario for the gene expression evolution. The three basic modes of evolution considered in this study are: (i) neutral evolution, (ii) constrained evolution (purifying/stabilizing selection) where the evolution of gene expression is biased against divergence from some optimum value, and (iii) adaptive evolution where the expression in different groups of species is biased toward different optimum values.

In this study, we use biological replicates to model within-species variations (Supplementary Figs. S3, S4, and S6; Supplementary Table S1). Although the animals used in biological replicates are isogenic, even between genetically identical multi-cellular organisms, such as *Drosophila*, there are significant individual-to-individual expression variations (Lin et al., 2016; Lee et al., 2018) often comparable to the expression variation between different laboratory strains of the same species (Supplementary Fig. S5).

Following standard practice, we assume that the evolution of gene expression follows a stochastic process. In particular, we assume that neutral evolution follows BM—a special case of the more general mean-reverting OU process—the broadly accepted model for the evolution of continuous traits such as gene expression (Hansen, 1997; Butler and King, 2004; Bedford and Hartl, 2009; King and Butler, 2009; Brawand et al., 2011; Chen et al., 2019). This model assumes that gene expression follows a stochastic process that is “attracted” toward some optimum value. The strength of the bias is modeled as a constant α. The optimum value to which the process is attracted is allowed to change over the evolutionary time, reflecting changes in environmental or other constraints acting on the trait.

Assuming neutral evolution as a null model, our goal is to use a hypothesis testing framework to uncover alternative modes of gene expression evolution. Toward this end we developed EvoGeneX, a mathematical framework to estimate the parameters of the process given the data. To account for the within-species variability, we assume that for each species *i*, there are several observations reflecting the within-species variability of the trait of interest (here expression of a specific gene). Denoting the value of the *k*th observation of the trait for species *i* by $y_{i,k}$, we set:

(1) $$Y_{i,k}\left(T_{i}\right)=y_{i,k}$$

where *T_i_* is the evolutionary time from the least common ancestor of all species in this tree to species *i*. Further, for $t\leq T_{i}$ we define:

(2) $$Y_{i,k}\left(t\right)=X_{i}\left(t\right)+\delta_{i,k}$$

where $X_{i}\left(t\right)$ follows the OU process:

(3) $$dX_{i}\left(t\right)=\alpha_{i}\left(\beta_{i}\left(t\right)-X_{i}\left(t\right)\right)dt+\sigma dB_{i}\left(t\right).$$

In Equation (6), the term $\sigma dB_{i}\left(t\right)$ models the increments of standard BM, and $\beta_{i}\left(t\right)$ is the optimum trait value for species *i* at time *t*. To make the estimation of model parameters tractable, $\beta_{i}\left(t\right)$ is assumed to change in discrete steps corresponding to the speciation events (internal nodes of the evolutionary tree) (Hansen, 1997; Butler and King, 2004; Chen et al., 2019) and only a limited number of changes (expression shifts) in the optimal expression value in one gene during the evolution are assumed.

Finally, $\delta_{i,k}∼N\left(0,\gamma \sigma^{2}\right)$ is an identically distributed Gaussian variable with mean 0 and variance $\gamma \sigma^{2}$ that models the within-species variability. Importantly, we assume that within-species variance is smaller than evolutionary variance, hence the factor γ is assumed to be less than 1. In addition, utilizing the estimate for asymptotic variance $\sigma^{2}∕2\alpha$ derived by Bedford and Hartl (2009), the condition that within-species variation is smaller the than between-species variation implies that 1∕2α≥γ, or alternatively, αγ≤1∕2.

**FIG. 1.**
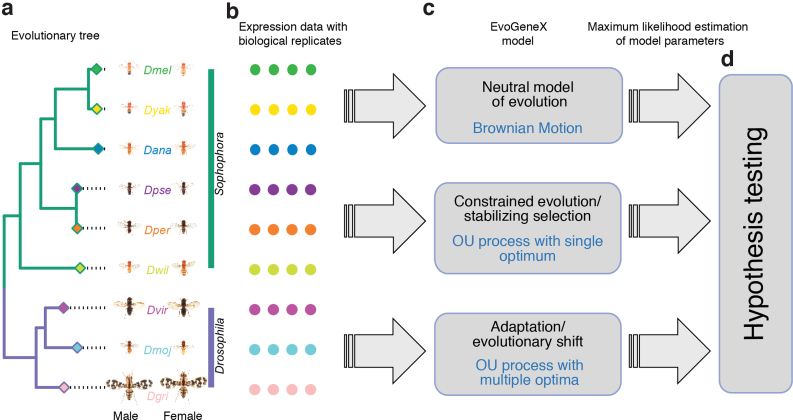
Workflow diagram. EvoGeneX takes as input an evolutionary tree (including evolutionary distances as branch lengths) **(a)** and normalized expression values across biological replicates and species **(b)**. It models evolutionary scenarios as stochastic processes as described in the text and uses maximum likelihood approach to fit the parameters of the model to the data **(c)**. For the adaptive model, the proposed adaptation regimes (here *Sophophora* and *Drosophila* subgroups) are required as part of the input. Finally, model selection is performed using hypothesis testing **(d)**. Photos of flies from *Drosophila* species are courtesy of Nicolas Gompel.

Given this general framework, we use hypothesis testing (Fig. 1d) to differentiate among the following evolutionary models: (i) neutral evolution: α=0, (ii) constrained evolution: α>0 and one common optimum $\beta_{i}\left(t\right)=\theta_{0}$ for all *i* and *t*, and (iii) adaptive evolution (evolutionary shift): α>0 and two different optima $\theta_{0},\theta_{1}$ representing two regimes with optimal β values $\theta_{1}$ in a specific subtree and $\theta_{0}$ in the rest of the evolutionary tree. In all cases, appropriate θ values together with α,σ,γ must be estimated from the data (i.e., values $y_{i,k}$). Toward this end, in Section 3, we describe our efficient method to compute Maximum Likelihood estimates of model parameters.

### 2.2. Accounting for within-species expression variations improves OU-based model

We use simulations to compare the performance of two competing models: a model that includes within-species expression variability versus a model that averages samples from the same species assuming that more than one sample is available. The former approach is implemented by our new EvoGeneX software, whereas the latter is implemented by OUCH (King and Butler, 2009)—currently the most popular software implementing an OU-based approach (Hansen, 1997; Butler and King, 2004; Bedford and Hartl, 2009; Chen et al., 2019).

We simulated expression values for 1000 genes using the stochastic differential Equations (2) and (3) governing our OU-based model of gene expression evolution on phylogenetic trees, for each setting of the parameters α,σ,β, and γ subject to biologically realistic constraints discussed next. For realistic evaluation of the effectiveness of our approach, we use for the simulation the same sequence-based phylogenetic tree (Chen et al., 2014) that we use for the real data. To match our real data, we simulate four replicates.

In addition, we include simulation with varying tree sizes and number of replicates as described next. In all of our simulations we set the expression level at the root (the most recent common ancestor of all the species) at $\beta_{i}\left(0\right)=1000$. We vary $\sigma^{2}$, the variance of the random change due to the BM, from 0.1% to 10% of the root expression and use the absolute values of 1,2,5,10,20,50,100. The ratio γ of within-species variance to $\sigma^{2}$ is varied as 1,1∕2,1∕4,1∕8,1∕16,1∕32,1∕64. We simulated all three modes of gene expression evolution.

In the neutral mode of evolution, the rate of attraction to the optimal value in the OU process,α, is set to 0 making the gene expression evolve as BM only. In the constrained mode of evolution, α of the OU process is positive and all nodes in the phylogenetic tree have the same optimum expression level. In our simulations, α is varied as 1∕8,1∕4,1∕2,1,2,4,8,16,32. Finally, in the adaptive mode of evolution, we considered the two-regime scenario where two sub-genera are assumed to converge toward two different optimal expression levels as described next.

To ensure an optimal performance of OUCH (King and Butler, 2009) in our evaluation, we let OUCH use the mean expression of simulated replicates and we refer to this model as OUCH.AV. Both methods, EvoGeneX and OUCH.AV, are evaluated as classifiers distinguishing (i) genes with constrained expression evolution from the genes with neutrally evolving expression, and (ii) genes with adaptive expression evolution from the genes with nonadaptive expression evolution.

In both cases, we use a variant of the area under precision recall curve metric that we call auPRC_*R*_ and is defined as the area under the part of auPRC curve for the recall value at most *R*, normalized so that maximum auPRC_*R*_ value is 1. This allows us to summarize how the precision changes as recall increases. Note that auPRC_1_ is the standard auPRC. We note that OUCH software can also be used to test for simultaneous selection of multiple traits by modeling those traits as a multivariate trait.

We asked whether such a model can be used to account for multiple replicates by modeling the expression of the replicates for a given gene as a multivariate trait. However, we found that such an approach produced results hardly better than a random method in all of our test settings (Supplementary Section S4 and Supplementary Fig. S18).

#### 2.2.1. Constrained evolution

**FIG. 2.**
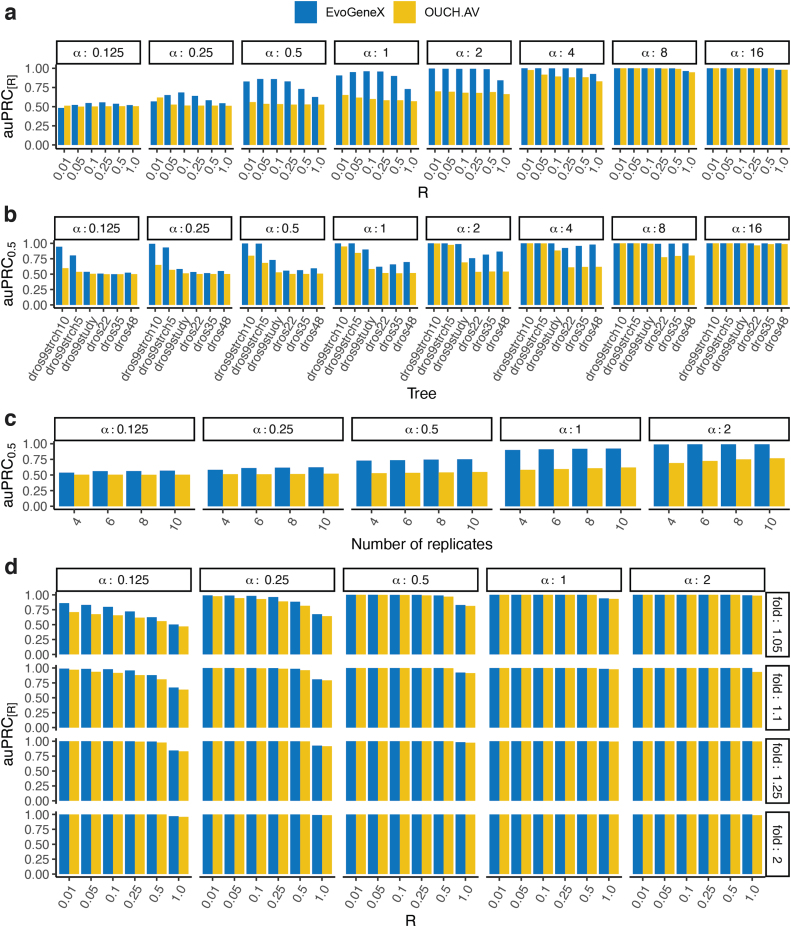
Performance of the methods on simulated data. Shown are values of auPRC at recall cut-off *R*. That is, auPRC_*R*_ is the area under the auPRC curve for the recall value at most *R*, normalized so that maximum auPRC_*R*_ value is 1. Each panel corresponds to different parameters of the simulation given within the white boxes. **(a)** Results for constrained evolution simulations, bars correspond to different values of *R* in auPRC_*R*_. **(b)** Results for varying species trees. Bars show auPRC_0.5_ for different tree shapes. **(c)** The values of auPRC_0.5_ for varying number of replicates. **(d)** Results for adaptive evolution simulations; the bars correspond to different values of *R* in auPRC_*R*_. auPRC, area under precision recall curve.

Figure 2a shows the auPRC_*R*_ of the two methods under the cutoff values R=0.01,0.05,0.1,0.25,0.5,1 for all tested values of α. The plots for each α aggregate the simulations for all $\sigma^{2}$ and γ such that $\sigma^{2}∕2\alpha \leq \gamma \sigma^{2}$, as the within-species variance $\gamma \sigma^{2}$ is assumed not to exceed asymptotic between-species variance $\sigma^{2}∕2\alpha$ (Bedford and Hartl, 2009). Overall, EvoGeneX outperforms OUCH.AV, though the improvement reduces as α increases. The higher the alpha, the expression becomes more constrained and it becomes easier for either of the two models to differentiate between the constrained and the neutrally evolved expression values.

Next, to understand the role of the number of species and evolutionary distances for the performance of the methods, we preformed simulations using trees with different number of species and evolutionary distances (branch lengths). First, starting from our initial nine-species tree *dros9study*, we obtained two trees by increasing the length of all edges by factors 5 and 10, respectively (trees: *dros9strch5, dros9strch10*). In addition, we included a large 48 species phylogeny (*dros48*) from Longdon et al. (2015). Despite a larger number of species in *dros48*, the evolutionary distances from the root to the leaves are smaller compared with *dros9study*.

We randomly extracted from it subtrees of 22 (*dros22*) and 35 (*dros35*) species. The methods had a better performance on the tree with larger distances even when the number of species was significantly smaller (Fig. 2b and Supplementary Figs. S18–S20). Adding more species while preserving the evolutionary distance to the root improves the performance of EvoGeneX but not OUCH (Fig. 2b and Supplementary Figs. S21–S23). These results demonstrate that the distribution of species on evolutionary tree is more important than the number of the species.

Surprisingly, including a larger number of biological replicates had a modest impact on EvoGeneX but benefits OUCH, suggesting that using EvoGeneX is particularly helpful when the number of biological replicates is small and estimated averages might be noisy (Fig. 2c and Supplementary Figs. S24 and S25).

Overall, our simulations demonstrate the advantage of a model that accounts for within-species variation. This outcome was expected and even assumed by some studies (Chen et al., 2019) but, surprisingly, it was not observed in previous simulations (Rohlfs et al., 2014). To understand the possible reason behind the discrepancy, we relaxed the constraints imposed by the assumption that within-species variation should be no bigger than between-species variation. We found that OUCH performs better in some settings where this assumption is strongly violated (Supplementary Figs. S12–S17). However, within-species variation is unlikely to be larger than between-species variation; thus, increased OUCH performance in those settings is not particularly useful.

#### 2.2.2. Adaptive evolution

In the adaptive mode of evolution, we first considered the two-regime scenario where the two sub-genera *Sophophora* and *Drosophila* are assumed to converge toward two different optimal expression levels. Previous studies indicate that OUCH-like models can detect adaptive evolution if the separation between optimal values for each regime is large (Cressler et al., 2015). Thus for the simulation of adaptive evolution, we also varied an extra parameter fold, the ratio of the optimum OU level in the *Drosophila* subgenus to that in *Sophophora* subgenus, as 0.5,0.8,0.9,0.95,1.05,1.1,1.25,2.

The scenarios for the two fold values 0.5 and 2 are symmetric to each other, so for 0.8, 1.25, and so on. Figure 2d shows the auPRC_*R*_ of the two methods for the fold values greater than 1. Both the methods are able to correctly detect the adaptive genes when the separation between two optima is larger due to a relatively large fold or α; however, EvoGeneX works better for the harder cases when both fold and α are low. Supplementary Figure S26 shows the same plots with similar trends for all fold values that we tested. Simulations using a larger tree form (Longdon et al., 2015) yielded similar results (Supplementary Figs. S9–S11 and S27–S31).

Overall, EvoGeneX achieves better auPRC in detecting constrained and adaptive genes, especially in the more difficult cases when selection/adaptation is not very strong. In addition, despite the need of estimating an additional parameter and considering larger input data, with more complex relations between them, EvoGeneX is only about twofold slower than OUCH to infer from four replicates (Supplementary Section S4.3 and Supplementary Fig. S32). Further, EvoGeneX scales well with the number of species in the tree and/or the number of replicates (Supplementary Figs. S33 and S34).

### 2.3. Stabilising selection and neutral gene expression evolution in *Drosophila*

In this study, we used expression data (Yang et al., 2018) from nine *Drosophila* species (Fig. 1a): *Drosophila melanogaster*, *Drosophila yakuba*, *Drosophila ananassae*, *Drosophila pseudoobscura*, *Drosophila persimilis*, *Drosophila willistoni*, *Drosophila virilis*, *Drosophila mojavensis*, and *Drosophila grimshawi* across both sexes and dissected into five body-parts: head, gonads, thorax, viscera, and abdomen (Supplementary Fig. S2 for an illustrative cartoon of the tissues) and a set of 8591 genes with one-to-one orthology in each species as determined in Yang et al. (2018) using multiple evidences.

**FIG. 3.**
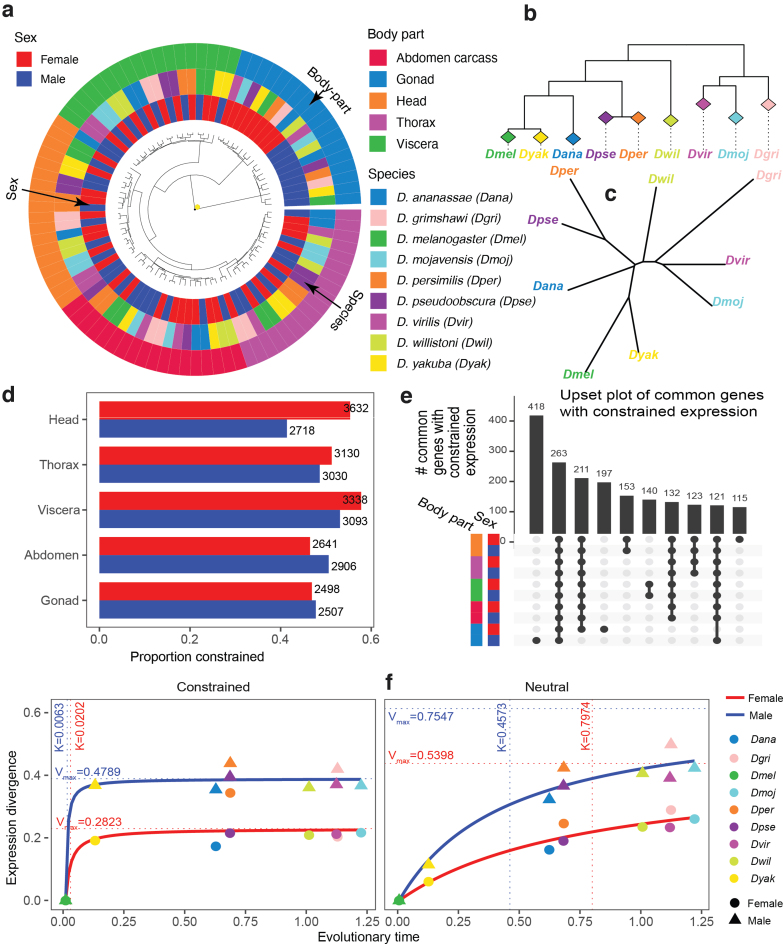
Constrained and neutral gene expression evolution in *Drosophila* genus. **(a)** Hierarchical clustering of gene expression data (using 1—Spearman's correlation). The outside ring represents body-parts, the middle ring species, and the inside ring sex. The main organizing factor is body-part (except for gonads that cluster first by sex). **(b, c)** Neighbor Joining trees constructed using gene expression similarity in specific body-parts (the tree shown is for the female head) illustrate that gene expression evolution **(c)** typically follows the species evolution **(b)**. **(d)** The proportions of the number of genes with constrained expression evolution to the number of genes for which neutral expression evolution could not be rejected. **(e)** UpSet plot (Lex et al., 2014) showing groups of common genes that have constrained expression in a subset of body-parts and sexes (black circles connected by vertical line). The groups are ordered by the decreasing number of common genes, and 10 most abundant groups are shown. **(f)** Modeling evolutionary dynamics in female gonads (ovaries) with the Michaelis-Menten curve. $V_{max}$ estimates equilibrium divergence, and *K* denotes the time to reach half of $V_{max}$. Constrained genes are characterized by smaller *K* and smaller $V_{max}$.

Given the large evolutionary distances within the *Drosophila* genus, we first asked whether the main observations about tissue-specific gene expression in mammals, where the distances are smaller, also hold for *Drosophila*. In particular, it has been observed that when the expression data across different tissues are clustered together the samples cluster first by tissue (Brawand et al., 2011; Chan et al., 2009; Sudmant et al., 2015; Chen et al., 2019). We found that this observation also holds for *Drosophila* (Fig. 3a) with one interesting caveat.

Specifically, when we used log gene expression values and Euclidean distances then head and thorax of the same species often clustered together (Supplementary Fig. S7). This co-clustering may reflect the fact that in the fly these two anatomical regions have extensive central nervous system components (brain and thorasic ganglia). Thus, both tissue-specific and species-specific trends can be observed, depending on the weight given to genes in the tails of the expression distributions. In addition, studies in mammals (Chen et al., 2019) report that expression evolution within individual tissues generally follows species evolution. We confirmed that this is also the case for *Drosophila* (Fig. 3c and Supplementary Table S2).

In particular, we could reconstruct the phylogenetic tree (either perfectly or with small Robinson-Foulds distances from the known sequence-based tree) using gene expression values in any specific body-part in either sex. Thus, although the evolution of gene expression is consistent with species evolution, the fact that all samples cluster predominately by body-parts, and in some cases by sex, suggests that gene expression evolution is also subject to strong tissue and sex-specific constraints.

Next, we used EvoGeneX to identify genes that are subject to constrained expression evolution and those for which the null hypothesis of neutral expression evolution could not be rejected. To focus on the genes that are relevant for a given tissue and sex, we included in this analysis only those genes that have normalised read counts of more than one in all four replicates and all species. Independent of organ and sex, and despite the stringency of our model, the hypothesis of neutral evolution could be rejected for more than 40% of the expressed genes (Fig. 3d).

In the following, we refer to a gene as “constrained” if the null hypothesis was rejected for that gene, and “neutral” otherwise. Note that, technically, this neutral group of genes with drifting expression also contains genes with weakly constrained expression that could not be confidently distinguished from the neutrally evolving group (analogous to nearly neutral sequence evolution; Ohta, 1973). Interestingly we found that gene expression evolution in females was often more constrained than in males with the biggest differences in head and viscera.

As discussed in the previous section, gene expression evolution for individual body-parts and sexes follows the previously estimated sequence-based species tree (Chen et al., 2014) either perfectly or with small Robinson-Foulds distances. We asked whether the occasional small differences between the sequence-based tree and expression-based tree would be eliminated if we use only the genes with neutral (or nearly neutral) expression evolution in the give body-part and sex only. Indeed, we found this was the case, although some minor differences persisted (Supplementary Table S2).

It is widely accepted that genes performing essential cell functions show constrained sequence evolution (Fisher, 1930). Therefore, we asked whether genes with constrained expression in one body-part or sex show constrained expression in other body-parts or in the opposite sex. Interestingly, the largest set of genes with constrained expression in more than one body-part occurred in the intersection of all the body-parts independent of the sex (Fig. 3e for overlaps of more than 100 genes and Supplementary Fig. S35 for more overlaps).

Gene Ontology (GO) Enrichment Analysis (Table 1) revealed that the most significantly enriched GO term for biological process for this common gene-set is mRNA splicing, via spliceosome. Alternative splicing is a highly conserved mechanism common to eukaryotes acting to modify the expression of specific isoforms in specific spatiotemporal patterns. We also considered gene expression constrained in smaller subsets of body-parts and sexes. For example, genes with constrained expression only in the head of both male and female flies were enriched in the GO terms for visual perception. Vision is an important evolutionary trait, and thus, many associated genes are, indeed, expected to undergo purifying selection. Finally, consistent with the fact that fly head and thorax share extensive central nervous system components, the common genes with constrained expression exclusively in those two body-parts were found to have enriched GO terms for the nervous system process and dendrite development.

**Table 1.** Gene Ontology Enrichment Analysis of the Common Constrained Genes

NS	ID	Description	*P*-value	*Q*-value
*{Testis}: 418 genes*				
BP	GO:0035082	Axoneme assembly	8.61e-08	0.00020
BP	GO:0007283	Spermatogenesis	6.22e-07	0.00098
BP	GO:1905349	Ciliary transition zone assembly	5.53e-06	0.00655
BP	GO:0030317	Flagellated sperm motility	0.00003	0.02019
BP	GO:0036158	Outer dynein arm assembly	0.00003	0.02019
BP	GO:0007018	Microtubule-based movement	0.00003	0.02019
BP	GO:0060271	Cilium assembly	0.00005	0.03227
BP	GO:0044458	Motile cilium assembly	0.00008	0.04034
BP	GO:0051039	Positive regulation of transcription involved in meiotic cell cycle	0.00011	0.04925
BP	GO:0097711	Ciliary basal body-plasma membrane docking	0.00011	0.04925
BP	GO:1905515	Nonmotile cilium assembly	0.00020	0.07974
BP	GO:0003341	Cilium movement	3.59e-10	1.70e-06
MF	GO:0045503	Dynein light chain binding	7.81e-06	0.01645
MF	GO:0045504	Dynein heavy chain binding	0.00002	0.01729
*{Head (both), Thorax (both), Viscera (both), Abdomen (both), Gonad (both)}: 263 genes*				
BP	GO:0000398	mRNA splicing, via spliceosome	1.08e-06	0.00304
BP	GO:0032436	Positive regulation of proteasomal ubiquitin-dependent protein catabolic process	1.28e-06	0.00304
BP	GO:0007029	Endoplasmic reticulum organization	0.00001	0.01672
MF	GO:0005515	protein binding	3.43e-07	0.00072
*{Head (both), Thorax (both), Viscera (both), Abdomen (both), Ovary}: 211 genes*				
		No significant GO terms		
*{Ovary}: 197 genes*				
		No significant GO terms		
*{Head (both)}: 153 genes*				
BP	GO:0007186	G protein-coupled receptor signaling pathway	2.04e-08	0.00010
BP	GO:0007601	Visual perception	0.00002	0.04425
*{Viscera(both)}: 140 genes*				
BP	GO:0016348	Imaginal disk-derived leg joint morphogenesis	0.00001	0.06227
*{Head (both), Thorax (both), Viscera (both), Abdomen(both)}: 132 genes*				
		No significant GO terms		
*{Head (both), Thorax (both)}: 123 genes*				
BP	GO:0007271	Synaptic transmission, cholinergic	1.35e-06	0.00642
BP	GO:0007268	Chemical synaptic transmission	6.31e-06	0.01112
BP	GO:0034220	Ion transmembrane transport	9.39e-06	0.01112
BP	GO:0050877	Nervous system process	9.39e-06	0.01112
BP	GO:0016358	Dendrite development	0.00006	0.05258
BP	GO:0042391	Regulation of membrane potential	0.00007	0.05519
MF	GO:0022848	Acetylcholine-gated cation-selective channel activity	4.77e-06	0.01005
MF	GO:0005230	Extracellular ligand-gated ion channel activity	0.00001	0.01266
*{Head (both), Thorax (both), Viscera (both), Abdomen (both), Testis}: 121 genes*				
		No significant GO terms		
*{Female head}: 115 genes*				
		No significant GO terms		

BP, biological process; GO, gene ontology; MF, molecular function; NS, name space.

### 2.4. Differences in evolutionary dynamics across sexes and organs

Next, we wanted to compare the evolutionary dynamics of gene expression between the sexes and the body-parts. To do this, we considered the relationship between the evolutionary distance (as determined by sequence evolution) of a species with respect to a reference, say *D. melanogaster*, and the expression divergence measured as 1−S(dmel,X) where S(dmel,X) is some measure of similarity, say the square of Spearman's correlation, between the replicate-averaged expressions in *D. melanogaster* and species *X* for a group of genes.

Among closely related taxa, such expression divergence is expected to be a linear function of time, but on larger distances it becomes nonlinear and saturates on a level that depends on the selection strength (Whitehead and Crawford, 2006; Bedford and Hartl, 2009) and potentially other biological limitations. This type of kinetics is similar to classical enzymatic kinetics, and it can be well approximated by the Michaelis-Menten curve $f\left(x\right)=xV_{max}∕\left(K+x\right)$. Figure 3f shows such curves for the constrained and neutrally evolving gene sets separately in the gonads of either sex. The parameter $V_{max}$ is the maximum (putative equilibrium value) of 1−S(dmel,X), and the parameter *K* is the value of *x* where $f\left(x\right)=V_{max}∕2$.

Thus, $V_{max}$ estimates the limit of the divergence of the given group of genes whereas *K* measures how fast this divergence is achieved: *K* close to zero corresponds to the situation where the value $V_{max}∕2$ corresponding to the half of the equilibrium divergence is immediately achieved. In contrast, larger *K* corresponds to slower relative divergence to the putative equilibrium at $V_{max}$. The values $V_{max}$ and *K* can be used to compare the evolutionary dynamics of gene expression between different organ systems, or gene groups.

Table 2 shows the values of $V_{max}$ and *K* for the two gene groups for all combinations of tissues and sexes (Supplementary Fig. S36 shows the fitted curves). As expected, $V_{max}$ values for the groups of genes with constrained expression evolution are systematically smaller than the $V_{max}$ values of genes with neutrally evolving expression for the same tissue/sex, although in the case of abdomen carcass the difference is small (consistent with the small *K* values for neutral expression evolution in abdomen carcass).

**Table 2.** Summary of $V_{max}$ and K of Constrained and Neutral Genes for Different Tissues and Sexes

Genes	V_max_				K			
	Constrained		Neutral		Constrained		Neutral	
Sex	Female	Male	Female	Male	Female	Male	Female	Male
Body-part								
Head	0.245	0.272	0.417	0.459	3.174e-02	2.506e-02	5.041e-01	8.012e-01
Thorax	0.274	0.288	0.403	0.532	4.843e-02	6.337e-02	3.486e-01	7.621e-01
Viscera	0.261	0.278	0.323	0.377	4.665e-02	5.348e-02	2.537e-01	3.996e-01
Abdomen	0.303	0.271	0.343	0.382	4.647e-02	4.613e-02	1.996e-01	1.927e-01
Gonad	0.282	0.479	0.540	0.755	2.022e-02	6.266e-03	7.974e-01	4.573e-01

Strikingly, male gonads show far less constrained expression evolution (larger $V_{max}$) than any other body-part in any sex. This is true not only for the constrained expression group, but also for the high $V_{max}$ for genes with neutrally evolving expression in male gonads. The testis shows the highest sex-biased expression in *Drosophila* (Arbeitman et al., 2002; Parisi et al., 2004), and it has been observed that the evolution of genes with sex-biased expression is accelerated (Begun et al., 2007; Haerty et al., 2007; Zhang et al., 2007; Meisel, 2011; Brown et al., 2014; Chen et al., 2014).

The high $V_{max}$ value for neutrally evolving genes in female gonads is surprising since, unlike males, female gonad gene expression in the constrained group is not particularly less constrained than other organ systems. These results show that the expression evolution of male and female gonads follows drastically different dynamics. Independently of sex, among the neutrally evolving gene expression trends, gonads have higher divergence than genes in any other tissue, whereas among the constrained group only gene expression in the testis shows less constraint relative to other organs. Importantly, these results do not depend on the species taken as reference (*D. melanogaster* here) as we observe similar results when *D. pseudoobscura* or *D. virilis* is taken as reference (Supplementary Fig. S37 and Supplementary Table S3). These appear to be general features of gene expression evolution in the phylogeny.

### 2.5. Adaptive evolution in the context of expression and sequence evolution

Differences in habitat, reproductive strategy, and other factors can lead to adaptive evolution in specific branches of the tree. We first tested whether EvoGeneX can detect examples of such adaptive evolution and examined the biological relevance of these findings. Finally, we considered the adaptive expression evolution in the context of sequence evolution.

#### 2.5.1. EvoGeneX reveals body-part specific adaptive expression evolution

Differences in habitat, reproductive strategy, and other factors should lead to evolutionary shifts of optimal expression values for some genes in a particular sex and/or organ system. As a result, the optimum value of a gene expression for a given sex and organ system might be different in different branches of the evolutionary tree.

Statistically, the detection of such evolutionary adaptation is very challenging and current methods have limited statistical power that has been attributed to the relatively small sizes of evolutionary trees for which tissue-specific gene expression data are available (Chen et al., 2019). We reasoned that by utilizing replicates for the estimation of within-species variability, EvoGeneX might have greater power to detect such adaptive evolution of gene expression. We used a hypothesis testing framework, and we tested whether both the neutral BM model and constrained model (OU with a single optimum expression) are rejected in favor of the adaptive evolution model (OU with multiple optima).

We tested for the evidence of adaptive evolution in two main settings. First, we used a two-regime setting where the regimes correspond to the two sub-genera: *Sophophora* and *Drosophila* (Fig. 1a). In addition, to capture potential adaptation in smaller subgroups of species, we also consider a three-regime setting where we partition the tree into three regimes of equal number of species (*D. melanogaster*, *D. yakuba*, *D. ananassae*), (*D. pseudoobscura*, *D. persimilis*, *D. willistoni*), (*D. virilis*, *D. mojavensis*, *D. grimshawi*). We call the first and the third group as *Melanogaster* and *Drosophila*, respectively, and the remaining species containing *Obscura* group and *D. willistoni* as *Obscurawil* due to the lack of standard nomenclature for such a group (Supplementary Fig. S1).

**FIG. 4.**
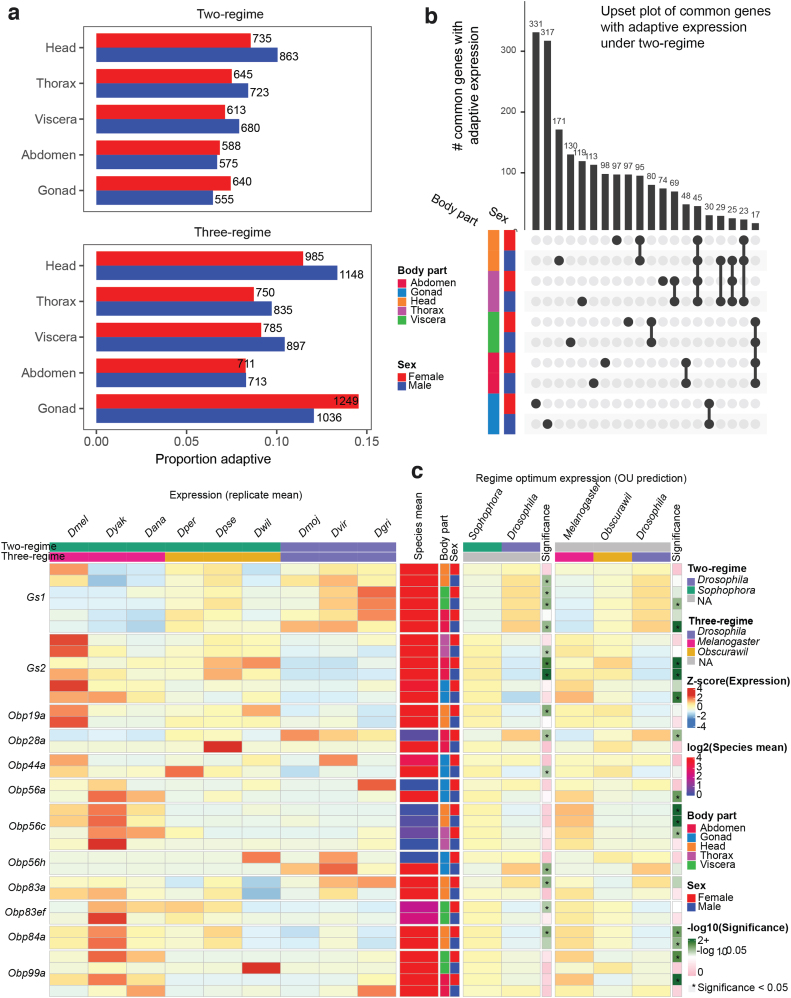
Adaptive gene expression evolution in *Drosophila* genus. **(a)** The proportion and number of adaptive genes in each body-part and sex for the two partitions into evolutionary regimes considered in the study. **(b)** UpSet plot showing number of common genes with adaptive expression evolution in two regimes (see Fig. 3e for full description). **(c)** Examples of adaptive genes. Shown from left to right are: z-score of gene expression in the given body-part and sex, average expression for the region and sex, and color code for body-part and sex (here we show both sexes for any body-part where adaption was detected, see Supplementary Fig. S41 for all body-parts), the optimal expression values obtained for each regime by EvoGeneX in the two-regime scenario and the max *p*-values for rejecting neutral and constrained evolution. Next, the same information is shown for the tree-regime scenario.

Interestingly, in the two-regime scenario, the highest number of genes with adaptive expression evolution was observed in the male head (Fig. 4a). For the three-regime scenario, in addition to the male head, high numbers of genes with adaptive expression evolution were observed in female gonads (Fig. 4a). Expression adaptation in male heads might be related to the fact that males have extensive sets of species-specific mating behaviors ranging from singing to displaying in mating arenas (Singh and Singh, 2014). We also note that there are differences in wiring and gross anatomy between male and female fly brains (Cachero et al., 2010). *Drosophila* ovaries also vary in morphology (Mahowald and Kambysellis, 1980). The set of adaptive genes uncovered by EvoGeneX can provide additional cues for sex-specific adaptation and warrants further investigation.

Interestingly, in contrast to the constrained expression evolution, where many genes with constrained expression were common to all the body-parts (Fig. 3e and Supplementary Figs. S38–S40), the adaptive evolution is largely body-part specific.

#### 2.5.2. Selected examples of adaptive expression evolution

It has been suggested that one important mechanism facilitating adaptation is gene duplication. Gene duplication is usually followed by the degeneration of one copy, but sometimes each of the duplicates evolves a specialized function. There are two Glutamine Synthase encoding genes in the *Drosophila* genus (encoded by *Gs1* and *Gs2*). These genes play an important role in nitrogen metabolism, amino acid synthesis, neurotransmitter recycling, and ammonia detoxification in the mitochondria (*Gs1*) and cytoplasm (*Gs2*) (Caizzi et al., 1990; Vernizzi et al., 2020). We found that *Gs1* expression was elevated, whereas reciprocally *Gs2* was depressed in the *Drosophila* subgenus, raising the possibility of compensatory expression evolution of these enzyme encoding genes (Fig. 4c).

One important element of adaptation is sensing the environment, including food sources, and mates. Therefore, we focused on the expression of odor binding proteins (Obps) as potential examples of adaptive change. *Obp* genes are members of a gene family evolved by duplication that has extensively diversified. Although the exact molecular role of a large number of Obps is not completely clear, they do play a role in pheromone-dependent species and sex recognition (Xiao et al., 2019). For example, *D. grimshawi* males paint attractive pheromones onto the substrate in mating arenas, known as leks, to attract competing males and female observers. In addition, both *D. virilis* and *D. mojavensis* use aggregation pheromones to mark resources for cooperative exploitation.

Expression could come under selection for higher expression in opposite sexes due to a switch in the role of a particular odor and/or hormone from intra-sexual to extra-sexual, or between females and males. Indeed, we found that the expression of many genes encoding Obps is subject to adaptive expression evolution, often in a sex-biased manner (Fig. 4c). As expected, such adaptive expression evolution of Obps is mostly found in the head (where olfactory organs are located) but sometimes in other body-parts as well (Fig. 4c). For example, the Obp28a protein binds floral odors (Gonzalez et al., 2020) and we found higher adaptive expression of *Obp28a* in *Drosophila* subgenus female abdomens.

In addition, although we have not detected statistically significant adaptation in males within the tested regimes, we saw a species-specific increase in *D. pseudoobscura* male abdomen. Next, we observed high adaptive expression of *Obp56a* in the melanogaster group testis. Expression of *Obp56a* is known to be downregulated in mated female *D. melanogaster* (McGraw et al., 2004). *Obp56c* expression showed adaptive increases in melanogaster group heads in the three-regime analysis. Knockdown of *Obp56h* results in rapid male remating and those males produce less 5-tricosene, an anti-aphrodisiac that inhibits male–male courtship (Shorter et al., 2016). Interestingly, we found *Obp56h* to be adaptive in testis. Very little is known about the *Obp83* and *Obp84* gene families or *Obp19a*, but these showed striking female-specific adaptation.

#### 2.5.3. Adaptive evolution in the context of sequence evolution

Our analysis demonstrated that adaptive expression evolution is tissue dependant and thus cannot be fully explained by sequence evolution, which is tissue independent. Therefore, we examined another interesting aspect of sequence evolution, which is the presence of rapidly evolving genes (REGs). Previous sequence-based studies identified 28 such REGs (Jagadeeshan and Singh, 2005) out of which CG12901 is currently annotated as an lncRNA, and it was not considered in our analysis. We were interested to see whether these REGs tend to be the subjects of adaptive expression evolution.

Out of the 27 REGs, only 19 had one-to-one orthologs in all species, thus they were included in our analysis using EvoGeneX. Interestingly, 13 out of these 19 genes were found to be adaptive in at least one tissue. In fact, one of these REGs with adaptive gene expression evolution is OS9 (olfactory-specific 9). This gene is adaptive in the male and female thorax and is involved in the sensory perception of smell, similarly to the Obps discussed in the previous subsection. However, although the number of adaptive REGs is higher than expected, the total number of REGs identified based on sequence analysis was too small to achieve statistical power in our analysis.

## 3. MATERIALS AND METHODS

### 3.1. Data and data/software availability

The expression data (Yang et al., 2018) were obtained from the NCBI GEO database under accession numbers GSE99574 and GSE80124, and the phylogenetic tree was obtained from Chen et al. (2014) (Supplementary Section S1). The source code for EvoGeneX is available at the NCBI public Github repository: https://github.com/ncbi/EvoGeneX, which also packages the *Drosophila* gene expression data. A software pipeline to analyze the data using EvoGeneX was built using JUDI (Pal and Przytycka, 2020). More information about EvoGeneX, *Drosophila* data and results of EvoGeneX on *Drosophila* data are available at Przytycka Lab webpage at *https://www.ncbi.nlm.nih.gov/CBBresearch/Przytycka/index.cgi#software*

### 3.2. EvoGeneX model and its parameters

EvoGeneX takes as its input a rooted evolutionary tree and the values of quantitative characters $y_{i,k}$ for all terminal taxa *i* and biological replicates *k*. Two sets of random variables, $X_{i}\left(t\right)$ at the taxa level and $Y_{i,k}$ at the replicate level, are used to model the evolution of trait value across time such that the observed trait value at time *T_i_*, the evolutionary time from the least common ancestor of all species in this tree to species *i*, is $Y_{i,k}\left(T_{i}\right)=y_{i,k}$. The two sets of random variables are governed by Equations (2) and (3).

EvoGeneX further assumes that the optimum value $\beta_{i}\left(t\right)$ of “attraction” in Equation (3) changes at speciation events only and remains constant along individual edges of the phylogenetic tree. The history of the *i*th lineage consists of a number, κ(i), of sequential branch segments demarcated by speciation events $0=t_{i}^{0}<t_{i}^{1}<t_{i}^{2}<\ldots <t_{i}^{\kappa \left(i\right)}=T_{i}$. Let all $t_{i}^{\tau -1}\leq t\leq t_{i}^{\tau }$ represent a *selective regime* where the evolution “attracts” toward a fixed optimum value $\beta_{i}^{\tau }$ of $\beta_{i}\left(t\right)$. EvoGeneX further simplifies the model by letting a small number, *R*, of distinct optimum values $\theta_{r}$, r=1,…,R, with each corresponding to one selective regime. In fact, one of the most interesting cases corresponds to the model with two optima where one branch of the tree follows a regime of optimum values $\theta_{1}$ and the rest of the tree $\theta_{0}$ (Brawand et al., 2011; Chen et al., 2019).

Let the binary variable $\beta_{i,r}^{\tau }$ indicate if the *τ*th branch on lineage *i* has operated in the *r*th regime. Then, we have $\beta_{i}^{\tau }=\sum_{r=1}^{R}\beta_{i,r}^{\tau }\theta_{r}$. Since each branch is associated with exactly one optimum, for each i,τ there is exactly one *r* such that $\beta_{i,r}^{\tau }=1$ and $\beta_{i,r\prime }^{\tau }=0$ for all r≠r′. Further, self-consistency requires that $\beta_{i,r}^{\tau }=\beta_{j,r}^{\eta }$ whenever lineage *i* and *j* share the branch ending in epoch $t_{i}^{\tau }=t_{j}^{\eta }$.

Thus, for a given tree, $y_{i,k}$s and $\beta_{i,r}^{\tau }$s, EvoGeneX estimates the parameters $\alpha ,\sigma ,\gamma ,\theta_{0},\theta_{1},\ldots ,\theta_{R}$.

### 3.3. Inference of EvoGeneX parameters using Maximum Likelihood estimates

In the following, it will be convenient to make use of matrix notation. Accordingly, we collect our random variables, $X_{i}\left(t\right)$ for the trait values at the taxa, and $Y_{i,k}\left(t\right)$ for the replicated trait values, in vectors x(t) and y(t), respectively, and our observed quantitative data in vector y with components $y_{i+\left(k-1\right)N}=Y_{i,k}\left(T_{i}\right)$, the observed value of replicate *k* of taxa *i* at the evolutionary time *T_i_*. The expected value of random variable y(t) at the taxa can be shown to be (Supplementary Section S3)


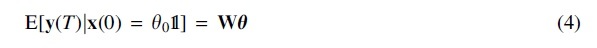


where 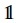 is a vector of all 1s, column vector 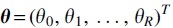 and the weight matrix *W* is dependent only on α among the parameters and has entries 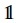

$$\begin{array}{ccc} W_{i+\left(k-1\right)N,0} & =e^{-\alpha T_{i}},W_{i+\left(k-1\right)N,r} & =\sum_{\tau =1}^{\kappa \left(i\right)}\left(e^{-\alpha \left(T_{i}-t_{i}^{\tau }\right)}-e^{-\alpha \left(T_{i}-t_{i}^{\tau -1}\right)}\right)\beta_{i,r}^{\tau } \end{array}$$

for i=1,…,N, k=1,…,M and r=1,…,R. Similarly, let an MN×MN matrix V give the covariance between species *i*, replicate *k* and species *j*, replicate *l* by the entry (Supplementary Section S3)


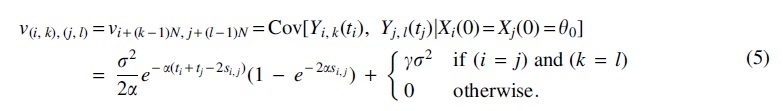


It is known that y is a multi-variate Gaussian ∼N(Wθ,V) with mean and co-variance given by Equations (1) and (2) (Hansen and Martins, 1996). Thus, the likelihood of the parameters α,σ,γ, and θ, given the data y is

$$ℒ\left(\alpha ,\sigma ,\gamma ,\theta |y\right)=\frac{1}{\sqrt{{\left(2\pi \right)}^{NM}detV}}\exp\left[-\frac{{\left(y-W\theta \right)}^{T}V^{-1}\left(y-W\theta \right)}{2}\right]$$

As maximizing ℒ is equivalent to minimizing U=−2logℒ, we seek to minimize


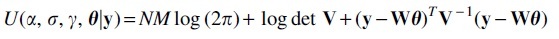


However, it can be noted that V has a nice structure and can be expressed as $\sigma^{2}\left(\tilde{V}+\gamma ℐ\right)$ where $\tilde{V}$ is dependent on α only among all the parameters and ℐ is an identity matrix of size *MN*. The elements of $\tilde{V}$ are given by ${Ṽ}_{\left(i,k\right),\left(j,l\right)}=\frac{1}{2\alpha }e^{-\alpha \left(t_{i}+t_{j}-2s_{i,j}\right)}\left(1-e^{-2\alpha s_{i,j}}\right)$. Thus, *U* can be expressed as


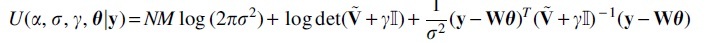


whose minimum can be estimated using any off-the-self nonlinear optimization solver.

However, we improve efficiency by utilizing Karush-Kuhn-Tucker conditions. By setting the partial derivatives of *U* with respect to σ and θ to 0 at an optimal solution $\left(\hat{\alpha },\hat{\sigma },\hat{\gamma },\hat{\theta }\right)$, we get

$$\begin{array}{ccc} {\hat{\sigma }}^{2} & =\frac{1}{NM}{\left(y-W\hat{\theta }\right)}^{T}{\left(\tilde{V}+\hat{\gamma }ℐ\right)}^{-1}\left(y-W\hat{\theta }\right),and,\hat{\theta } & ={\left(W^{T}{\left(\tilde{V}+\hat{\gamma }ℐ\right)}^{-1}W\right)}^{-1}W^{T}{\left(\tilde{V}+\hat{\gamma }ℐ\right)}^{-1}y \end{array}$$

Thus, instead of minimizing function *U* of four parameters α,σ,γ and θ, it is enough to minimize a new function of two parameters, α and $\gamma ,Ũ\left(\alpha ,\gamma \right)=NM\left[1+\log2\pi {\hat{\sigma }}^{2}\left(\alpha ,\gamma \right)\right]+\log det\left(\tilde{V}+\gamma ℐ\right)$ where the following two intermediate functions


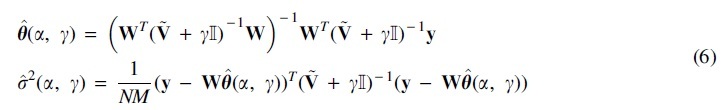


give the values of the remaining two parameters σ and θ at the optimal solution.

### 3.4. Maximum Likelihood estimates for BM model

To compare EvoGeneX model of evolution with the BM model, we need to compute maximum likelihood estimates for the BM model accounting for the within-species variation. The BM is a simplified model in comparison to EvoGeneX: There is no “attracting” optimal values and hence there is no α parameter and θ has only one value, $\theta_{0}$, to be estimated. Using a procedure similar to the previous section, we estimate $\sigma ,\gamma ,\theta_{0}$ from the given data (Supplementary Section S3).

### 3.5. Computing statistical significance

We use statistical hypothesis testing to decide which of the three different modes of evolution the trait has undergone: (i) neutral, (ii) constrained, and (iii) adaptive (Section 3.1). For this purpose, we use the likelihood ratio test (Supplementary Section S3).

## 4. CONCLUSIONS

Natural selection is a complex organism-level force that has well-studied effects on the sequence of genes, gene duplication and divergence, and de novo gene formation. However, because all cell types have the same genome, the genome sequence is always a compromise for the expression optimum in any given tissue. Sequence is selected based on the survival of a genotype, whereas genes are almost always expressed in multiple contexts within an organism and are thus free to optimize expression levels in different cell types through gene regulatory networks. Thus, studies of the evolution of gene expression are fundamental to understanding the molecular basis of phenotypic trait evolution.

Early in the genomic era, biological replication was not often a priority. The steadily decreasing cost of sequencing now motivates researchers to measure gene expression in multiple biological replicates. Our own data utilized in this study includes four replicates per each species, sex, and body-part. We started with the assumption that information about within-species variation should boost the performance of the OU-based method.

We then developed EvoGeneX, which implements an OU model that includes within-species variation estimated using biological replicates. Overall, our results demonstrate that, by taking full advantage of the existing gene expression data, EvoGeneX provides a new and more powerful tool to study gene expression evolution relative to the currently leading method.

We note that purposefully generated data are not always available, and that it would be useful to use data generated in a variety of studies with different initial objectives. Reusing data produced in different labs for different reasons should increase noise. As per our simulations, large variance is not a problem for our method. However, the assumption that the within-species variance is not to exceed asymptotic between-species variance is relevant for the good performance of the model. For a very noisy data that, for some reason, violates this assumption, averaging of the replicates might be preferred. However, one could argue that this might be better addressed by collecting new data.

Finally, it has been assumed that an OU model performs better on a tree with more species. However, our simulations indicate that it is more important to consider how the species are distributed over the evolutionary tree than how many species are used. Our simulations demonstrate, that despite the small number of species used in our study, the performance of either of the two methods was superior to the performance that could be achieved on a much larger tree but utilizing more closely related species. These analyses help to guide the data collection effort by balancing the number species, their evolutionary distances, and number biological replicates to achieve the best results.

There have been several studies of *Drosophila* expression evolution as it relates to sex, protein coding sequence, and expression breadth (Ellegren and Parsch, 2007; Meisel, 2011; Campos et al., 2018). Some of those studies have been contradictory or focused on a single prevalent pattern of divergence rather than distinct evolutionary mechanisms. We used EvoGeneX to provide a mathematical framework for an extensive analysis of sex and body-part specific gene expression evolution in the *Drosophila* genus. In agreement with some of the previous studies, we showed that neutral gene expression evolution can be confidently rejected in favor of constrained evolution (presumably due to purifying selection) in nearly half of the genes.

However, it also showed that for a comparable number of genes the neutral expression evolution could not be rejected, indicating either truly neutral expression evolution or expression evolution under very weak constraints.

Previous studies in mammals observed that gene expression evolution in testis is the least constrained among the organs analyzed (Chen et al., 2019). However, these studies did not include female gonads. Our results show that, among the neutrally (or nearly neutrally) evolving gene expression patterns, gonads evolve faster than other tissues, and male gonads evolve faster than female gonads. However, among the genes identified as constrained, only the testis showed relaxed constraints relative to any other tissue, whereas the constraints acting on female gonads were comparable to the constraints acting on other tissues. Thus, there are interesting differences in the evolutionary dynamics of male and female gonads.

Using EvoGeneX, we also detected interesting examples of adaptive evolution, including prominent examples of odor binding proteins as well as an example of adaptive evolution followed by gene duplication. Interestingly, in contrast to the constrained expression expression evolution where many genes with constrained expression were common to all the body-parts, we found that adaptive evolution tends to be body-part specific.

In summary, our tool performs well to analyze data and show that gene expression is an important trait under selection and subject to drift at the body-part level, to optimize the survival and reproduction of the individual—the unit of selection. This is likely also to be true at the cell-type level, and it is likely that data allowing testing of this hypotheses will soon be available. Although our study is focused on gene expression evolution, the model can be applied to the evolution of other quantitative traits, including protein abundance.

It is generally assumed that the constraints imposed on the gene expression evolution are related to the constraints imposed on protein abundance. Evolutionary models such as EvoGeneX hold the promise of providing a better understanding of this relation once protein abundance data across a large set of species, organs, and sexes become available.

## Supplementary Material

Supplemental data
