# Supplementary material for: Stochastic Modeling of Gene Expression Evolution Uncovers Tissue- and Sex-Specific Properties of Expression Evolution in the Drosophila Genus
Source: J Comput Biol. 2023 Jan 5;30(1):21–40. doi: 10.1089/cmb.2022.0121 (PMC9917317; doi:10.1089/cmb.2022.0121)
Supplement: Supplemental data [file Supp_DataS1.pdf]

Supplementary Information for "Stochastic modeling of gene expression evolution uncovers tissue and sex specific properties of expression evolution in the *Drosophila* genus"

Soumitra Pal[1] Brian Oliver[2,*] Teresa M. Przytycka[1,*]

S1 Data

S1.1 Phylogenetic tree

We obtained the phylogenetic tree for 12 *Drosophila* species (Z.-X. Chen et al. 2014) in Newick format via personal communication. The tree was trimmed to 9 *Drosophila* species of our interest using `drop.tip` function of `ape` R package. The trimmed Newick file used in our analysis is reproduced here.

```
((dgri:0.206091,(dmoj:0.235899,dvir:0.134088):0.067592):0.235776,
 (dwil:0.442195,((dper:0.006819,dpse:0.005611):0.262193,
                  (dana:0.316433,(dyak:0.061958,dmel:0.059667):0.244496):0.107103
                ):0.152356
 ):0.117888
);
```

Figure 1a in the main text shows a drawing of the phylogenetic tree. Additionally it shows the branches on which the two levels of 'optimum' expression corresponding to *Drosophila* and *Sophophora* subgenera in the Ornstein-Uhlenbeck model under two-regime are assumed to be active. Figure S1 in this document shows the branch colors according to the 'optimum' levels active on them under three-regime for the subgenera *Melanogaster* and *Drosophila* and the rest of the species that includes *D. willistoni* and the *Obscura* group and we name as *Obscurawil.*

S1.2 Gene expression data

The expression data was obtained from (Yang et al. 2018). The data is available at the NCBI GEO database with accession numbers GSE99574 (for non-Hawaiian species) and GSE80124 (for Hawaiian species). Figure S2 shows a cartoon for the nomenclature of different tissues. We took the samples from the body-parts for which all 4 replicates were available. These were: head (HD), Thorax (TX) Abdomen Carcass (AC), Viscera (VC) and Gonad (GO). We used the strains w1118 and G1 of *D. melanogaster* and *D. grimshawi* respectively.

The raw read counts are available in the supplementary folders in the GEO database. The data was normalized to allow for the gene level (between samples) analysis (Yang et al. 2018). Specifically, all body-parts and all samples were first normalized using DESeq2 (Love et al. 2014) and then only the gene-level normalized counts for 8591 orthologous genes were used for our evolutionary analysis. The mapping of *D. melanogaster* gene-IDs to the ortholog-IDs in other species were also downloaded from the GEO supplementary folders.

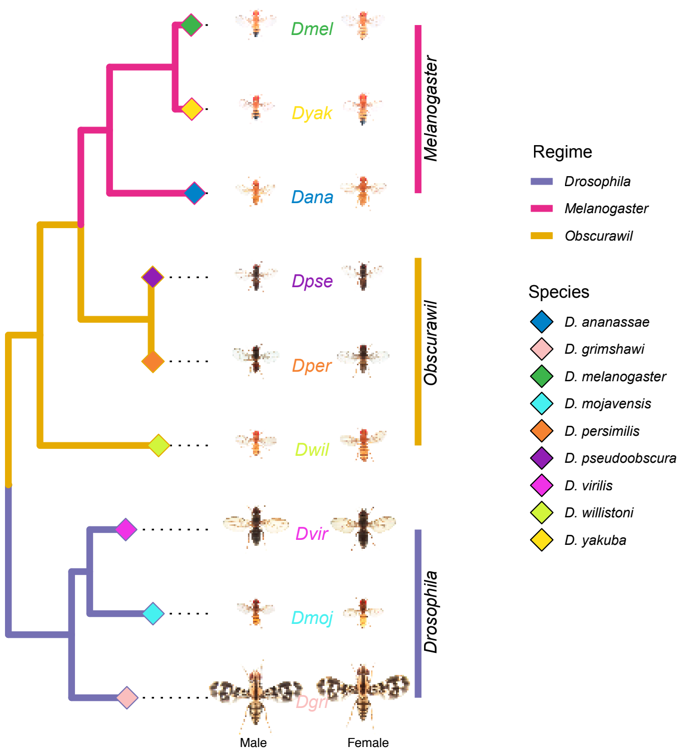

Figure S1: **Three-regime scenario**

S2 Exploratory analysis of data

S2.1 Summary statistics

The expression of the replicates were found to have 'good' correlations. Table S1 shows the Pearson's correlation coefficients for each pair among the 4 replicates for each species, body-part and sex. Most of the coefficients are more than 0.9. Figure S3 shows the summary of expression values each replicate and Figure S4 shows the violin plots.

S2.2 Between-strains expression correlation

The original dataset (Yang et al. 2018) contains expression data from multiple strains of *D. melanogaster* and *D. grimshawi*. To compare expression divergence between two strains of the same species relative to the divergence between the replicates of the same strain, we additionally considered the expression data from the 'OreR' strain of *D. melanogaster* and computed within-strain between-replicates, within-species between-strains and between-species expression correlations as shown in the box-plots in Figure S5. In comparison to the between-species, the between-replicates correlations are similar to the between-strains correlations for the same species. Note that we considered 'OreR' strain only for computing these correlations, and not for other analyses in the paper.

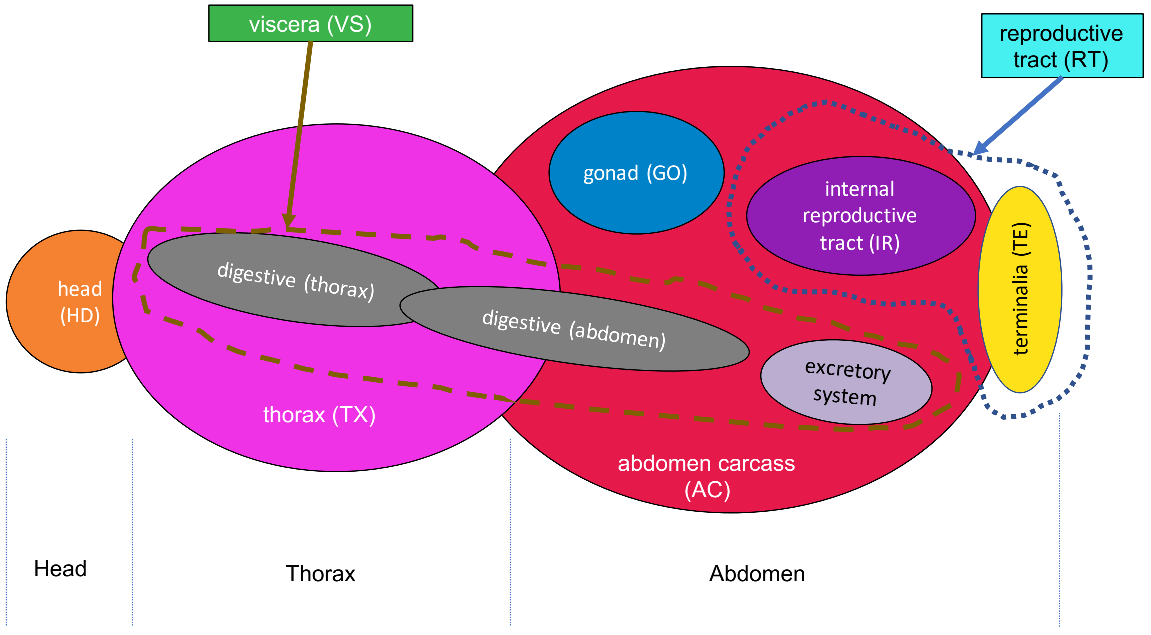

Figure S2: **An illustrative cartoon for the body-parts of a fruit-fly from where the samples were taken and bulk RNA-seq data was generated.** Only the following five body-parts were used for our analysis as only they all had all 4 replicates: head (HD), thorax (TX), viscera (VS), abdomen carcass (AC) and gonad (GO).

Table S1: **Pearson's correlation coefficients between any pair of replicate expressions.**

**Species**	**Body part**	**Sex**	R1vR2	R1vR3	R1vR4	R2vR3	R2vR4	R3vR4
**Dmel**	**Head**	**Female**	0.994284	0.990478	0.992961	0.964538	0.972433	0.985174
		**Male**	0.998499	0.996507	0.996663	0.997282	0.996951	0.998052
	**Thorax**	**Female**	0.994681	0.993190	0.994309	0.985519	0.990970	0.992146
		**Male**	0.991482	0.993884	0.992369	0.987842	0.992546	0.994792
	**Viscera**	**Female**	0.993888	0.997742	0.991912	0.982094	0.993345	0.979998
		**Male**	0.987480	0.984554	0.966164	0.990095	0.987106	0.987937
	**Abdomen**	**Female**	0.988949	0.984882	0.994223	0.990497	0.993050	0.991092
		**Male**	0.992132	0.980661	0.987349	0.970172	0.979731	0.991954
	**Gonad**	**Female**	0.990568	0.979192	0.983561	0.991353	0.995576	0.977971
		**Male**	0.999377	0.999368	0.999392	0.998940	0.999083	0.999232
**Dyak**	**Head**	**Female**	0.984920	0.977258	0.996401	0.983580	0.988325	0.991220
		**Male**	0.995152	0.983804	0.981935	0.993153	0.991170	0.984255
	**Thorax**	**Female**	0.990890	0.994924	0.995122	0.883165	0.905518	0.906166
		**Male**	0.996737	0.939085	0.925254	0.969958	0.960632	0.964784
	**Viscera**	**Female**	0.984629	0.980794	0.993663	0.919146	0.959881	0.971992

Continued on next page

Table S1 – continued from previous page

Species	Body part	Sex	R1vR2	R1vR3	R1vR4	R2vR3	R2vR4	R3vR4
		**Male**	0.989315	0.799956	0.751725	0.992267	0.992102	0.805981
	**Abdomen**	**Female**	0.993592	0.995429	0.996583	0.968139	0.980062	0.980141
		**Male**	0.963463	0.936113	0.942848	0.979312	0.939376	0.927537
	**Gonad**	**Female**	0.994961	0.996948	0.993684	0.987874	0.975705	0.989799
		**Male**	0.994361	0.994777	0.994190	0.973175	0.964849	0.982665
**Dana**	**Head**	**Female**	0.990367	0.989508	0.967599	0.994900	0.979750	0.997848
		**Male**	0.990991	0.992224	0.993568	0.982729	0.987931	0.995473
	**Thorax**	**Female**	0.941168	0.994460	0.957395	0.987055	0.960592	0.997124
		**Male**	0.944573	0.963127	0.994388	0.907228	0.987476	0.983303
	**Viscera**	**Female**	0.962710	0.932637	0.982137	0.986278	0.975557	0.963491
		**Male**	0.993044	0.990088	0.991979	0.979297	0.988458	0.983946
	**Abdomen**	**Female**	0.991240	0.978971	0.959236	0.983975	0.967665	0.996980
		**Male**	0.971883	0.964883	0.993465	0.965458	0.989691	0.990525
	**Gonad**	**Female**	0.983187	0.959404	0.988990	0.977506	0.995813	0.990288
		**Male**	0.985149	0.978059	0.997103	0.991309	0.991224	0.990138
**Dpse**	**Head**	**Female**	0.993112	0.943349	0.946848	0.838438	0.840575	0.967611
		**Male**	0.995843	0.989104	0.994996	0.985457	0.986487	0.987875
	**Thorax**	**Female**	0.954700	0.986829	0.948448	0.948178	0.898844	0.974722
		**Male**	0.909400	0.909106	0.979517	0.931304	0.965471	0.969429
	**Viscera**	**Female**	0.991218	0.996787	0.984053	0.989554	0.979395	0.992060
		**Male**	0.982862	0.980371	0.974773	0.918769	0.859093	0.907442
	**Abdomen**	**Female**	0.970571	0.979348	0.921108	0.952402	0.873621	0.992262
		**Male**	0.771104	0.840642	0.802293	0.934902	0.758733	0.844536
	**Gonad**	**Female**	0.995431	0.982422	0.971455	0.928933	0.917062	0.962804
		**Male**	0.996609	0.998662	0.996173	0.997104	0.993257	0.997435
**Dper**	**Head**	**Female**	0.984080	0.982618	0.960732	0.986538	0.988135	0.978817
		**Male**	0.979499	0.988659	0.992045	0.980987	0.990617	0.988123
	**Thorax**	**Female**	0.975192	0.973009	0.960853	0.974363	0.974062	0.967320
		**Male**	0.935970	0.864674	0.958101	0.908037	0.980721	0.950267
	**Viscera**	**Female**	0.995835	0.985633	0.986375	0.993985	0.993464	0.990415
		**Male**	0.979966	0.956007	0.977611	0.988133	0.995732	0.974185
	**Abdomen**	**Female**	0.989332	0.958943	0.977370	0.959812	0.974976	0.960716
		**Male**	0.965142	0.970291	0.970791	0.964362	0.995695	0.957293
	**Gonad**	**Female**	0.961425	0.981003	0.990609	0.970611	0.995859	0.990006
		**Male**	0.979448	0.935380	0.918542	0.985041	0.993212	0.934277
**Dwil**	**Head**	**Female**	0.997698	0.979226	0.978166	0.976718	0.976427	0.982758
		**Male**	0.997537	0.997281	0.997059	0.997151	0.998717	0.998613
	**Thorax**	**Female**	0.982212	0.968952	0.968229	0.860930	0.878991	0.917237
		**Male**	0.991469	0.993756	0.979984	0.990890	0.992521	0.981754
	**Viscera**	**Female**	0.945545	0.991329	0.937209	0.973417	0.928403	0.981598
		**Male**	0.975367	0.989616	0.987774	0.969765	0.994070	0.990410
	**Abdomen**	**Female**	0.994095	0.978959	0.978260	0.963703	0.958889	0.949065

Continued on next page

Table S1 – continued from previous page

Species	Body part	Sex	R1vR2	R1vR3	R1vR4	R2vR3	R2vR4	R3vR4
		**Male**	0.985270	0.984225	0.978118	0.962820	0.956170	0.973740
	**Gonad**	**Female**	0.986676	0.969084	0.993086	0.975491	0.940041	0.907877
		**Male**	0.997202	0.998340	0.996234	0.996643	0.991342	0.996868
**Dvir**	**Head**	**Female**	0.998306	0.954397	0.956706	0.988088	0.990246	0.921360
		**Male**	0.995746	0.980533	0.979635	0.988538	0.986982	0.992969
	**Thorax**	**Female**	0.977790	0.993576	0.981624	0.993512	0.976819	0.990958
		**Male**	0.992085	0.994168	0.987029	0.994137	0.995288	0.990043
	**Viscera**	**Female**	0.997040	0.990264	0.988539	0.977953	0.981485	0.981438
		**Male**	0.985694	0.981124	0.986304	0.981461	0.991149	0.990728
	**Abdomen**	**Female**	0.991785	0.995693	0.998636	0.997905	0.993871	0.997094
		**Male**	0.992116	0.994619	0.982653	0.991610	0.993747	0.983224
	**Gonad**	**Female**	0.991769	0.987540	0.988091	0.972785	0.978105	0.991981
		**Male**	0.993468	0.995883	0.998775	0.998449	0.996464	0.997551
**Dmoj**	**Head**	**Female**	0.962843	0.960825	0.994306	0.943728	0.994027	0.992354
		**Male**	0.989710	0.988592	0.993848	0.991283	0.994582	0.991813
	**Thorax**	**Female**	0.961575	0.963535	0.992263	0.959057	0.984834	0.988811
		**Male**	0.958826	0.950014	0.874381	0.987367	0.924431	0.971886
	**Viscera**	**Female**	0.979529	0.982159	0.986241	0.957079	0.975849	0.974537
		**Male**	0.987829	0.937080	0.941667	0.928200	0.933017	0.985201
	**Abdomen**	**Female**	0.931998	0.950575	0.981828	0.951998	0.995354	0.983527
		**Male**	0.924865	0.966528	0.929062	0.980075	0.937620	0.977476
	**Gonad**	**Female**	0.972749	0.991417	0.986517	0.940641	0.988575	0.956016
		**Male**	0.970933	0.982022	0.996073	0.987983	0.992238	0.996666
**Dgri**	**Head**	**Female**	0.974102	0.986001	0.942319	0.993673	0.975433	0.984177
		**Male**	0.996217	0.979935	0.980031	0.985497	0.992074	0.966464
	**Thorax**	**Female**	0.979418	0.982102	0.951328	0.984850	0.971397	0.981054
		**Male**	0.996772	0.991701	0.993935	0.992323	0.993478	0.993230
	**Viscera**	**Female**	0.950148	0.976583	0.966260	0.983401	0.970601	0.984913
		**Male**	0.972196	0.963684	0.987954	0.941835	0.978413	0.984942
	**Abdomen**	**Female**	0.960385	0.929732	0.914441	0.854547	0.749440	0.759291
		**Male**	0.931437	0.940168	0.927029	0.925515	0.900744	0.896803
	**Gonad**	**Female**	0.846158	0.879609	0.682405	0.978817	0.742362	0.880504
		**Male**	0.991673	0.915842	0.931773	0.990275	0.988463	0.906694

S2.3 Principal component analysis

Figure S6 shows the PCA clustering obtained from the average expression of all replicates. The PCA was done using scikit-learn library in Python. The similarity measure was 1-Spearman's correlation coefficient. The analysis confirms clustering by body-parts.

S2.4 Hierarchical clustering

Hierarchical clustering was obtained by UPGMA method (R function `agnes` method `average`). Figure S7a (repeated from Figure 3a in main text) shows the clustering obtained when the distance

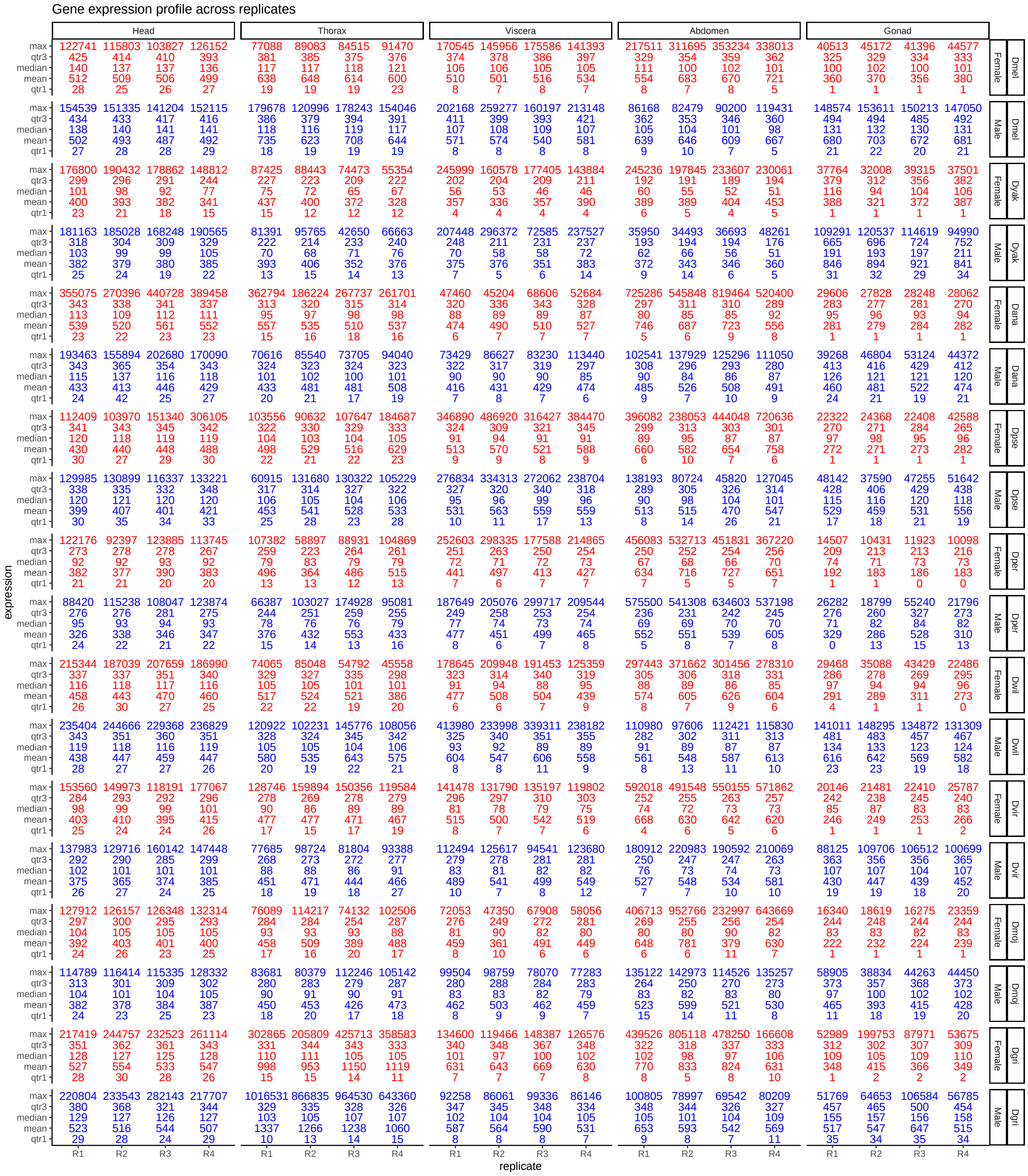

Figure S3: **Summary statistics of the replicate expression across species, body-parts and sex.**

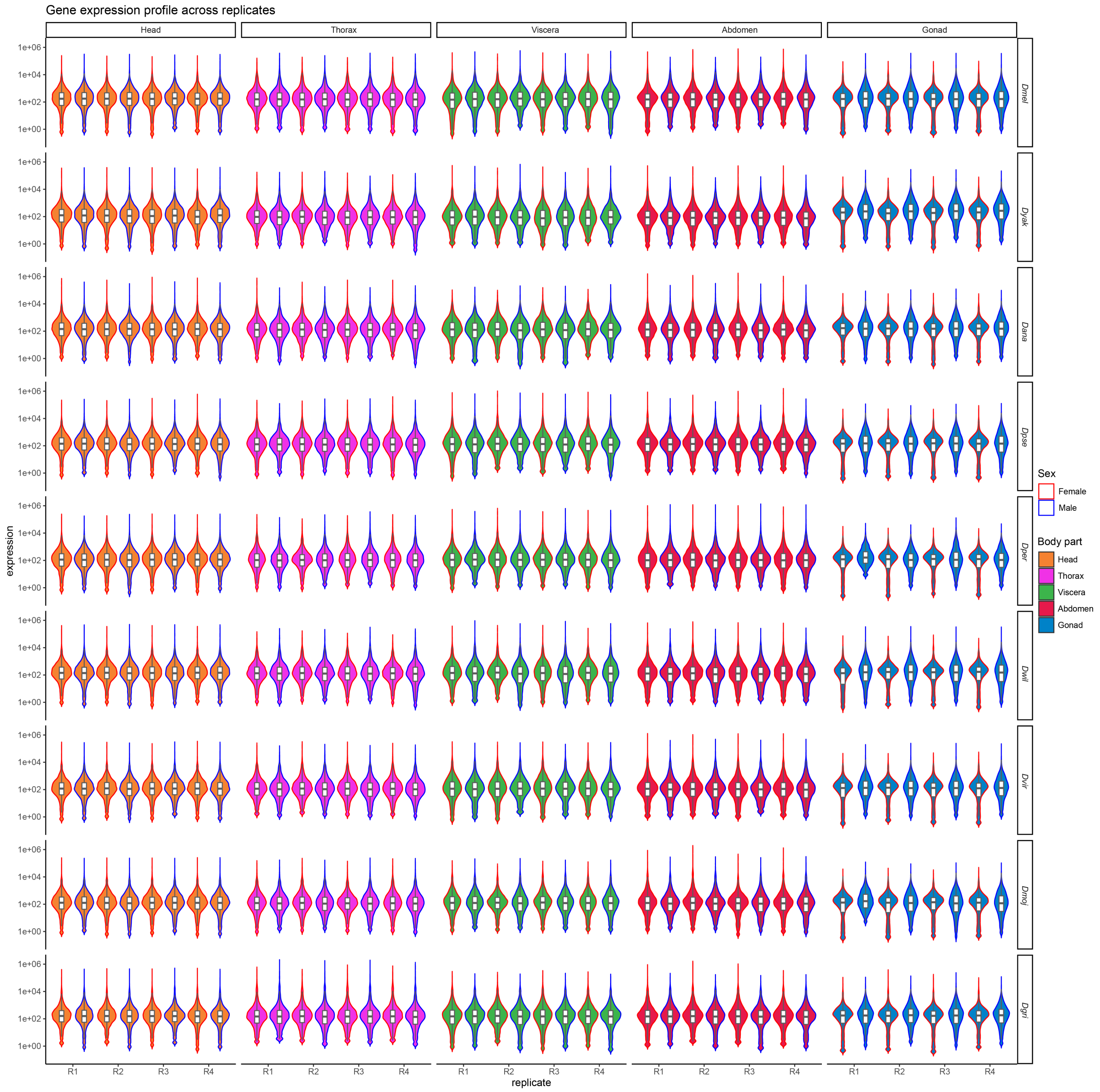

Figure S4: **Violin-plots of the replicate expression across species, body-parts and sex.**

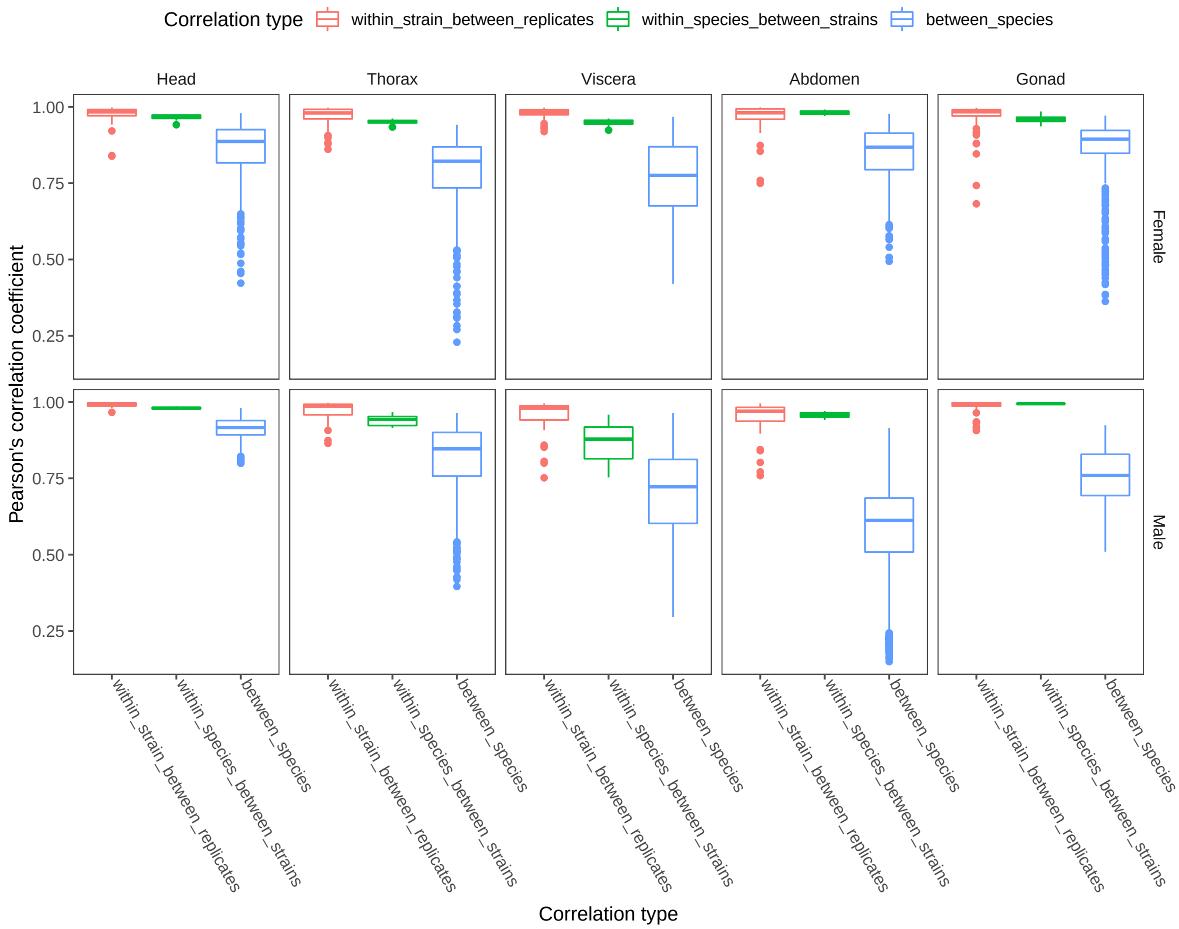

Figure S5: **Pearson's correlation coefficients between two samples of expression data under the following three categories: i) within-strain and between-replicates, ii) within-species and between-strains, and iii) between-species**.

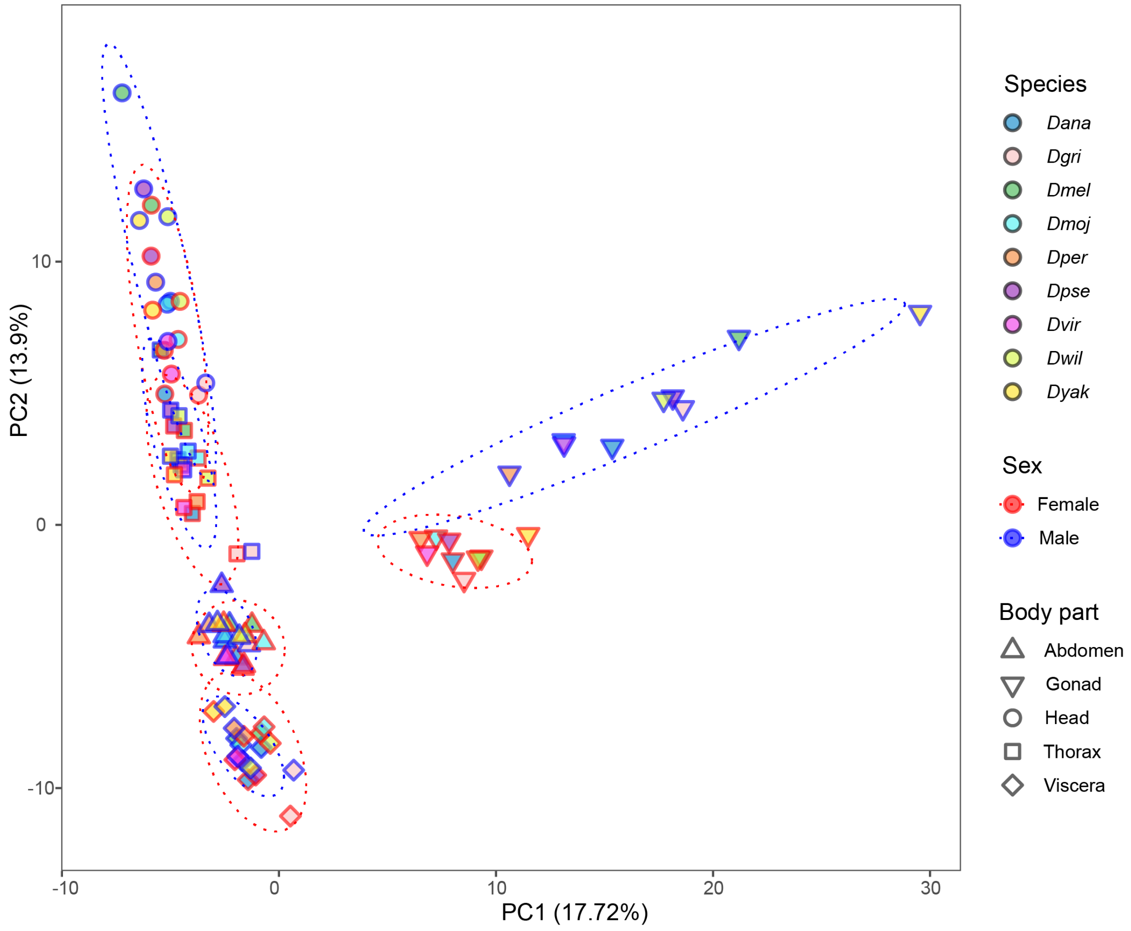

Figure S6: **Principal Component Analysis of gene expression data (replicate mean).** The gonad expressions cluster by sex first and then by species whereas non-gonad expressions cluster by body-parts first and then by species and sex, in that order.

given by 1-Spearman's correlation coefficient on the normalized gene expression values. We observe that the expression data predominantly clusters by tissue. However, when we used log gene expression values and Euclidean distances then related tissues of the same species often clustered first (Figure S7b). Specifically, with this similarity measure, head and thorax typically cluster together. This reflects the fact that in fly these two body-parts are related. Thus both tissue-specific and species-specific trends can be observed, depending on the weight given to genes in the tails of the expression distributions.

S3 EvoGeneX

EvoGeneX takes as its input i) a rooted evolutionary tree, ii) the values of quantitative characters (with biological replicates) for all terminal taxa, and iii) the labelling of all nodes for which regime it belongs. EvoGeneX estimates i) the parameters associated with three models, and reports ii)

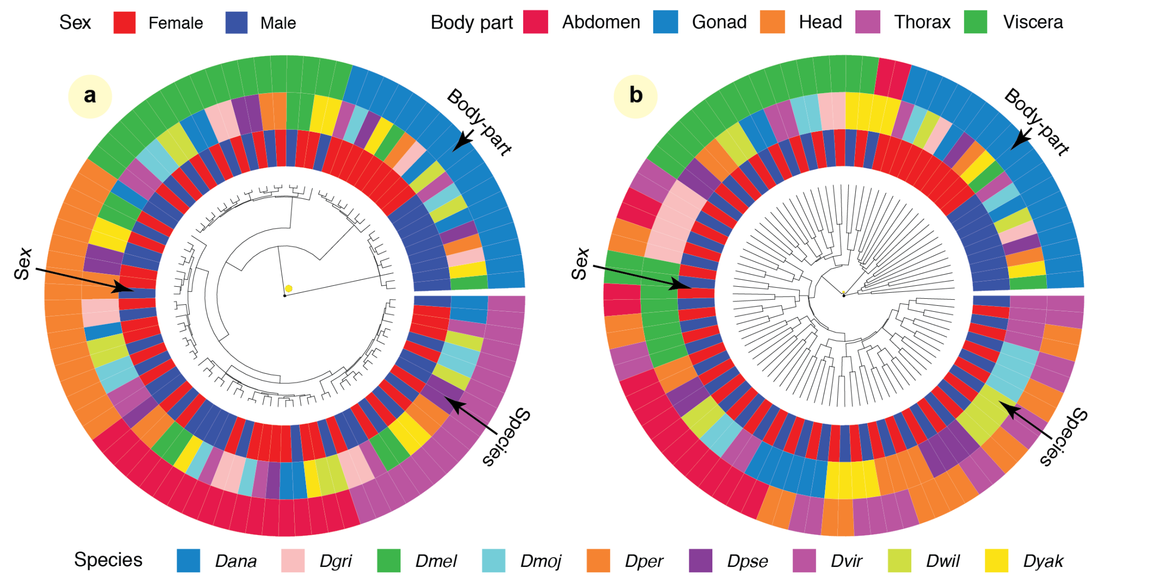

Figure S7: **Hierarchical clustering of gene expression data using two different distance matrices.** The outside circles correspond to body-part, the middle circles to species and the inside circles to sex. (**a**) Using Spearman's correlation: The main organizing factor is cluster first (except for gonads which cluster first by sex). (**b**) Using Euclidean distance: Here, related tissues, e.g., head, thorax, and abdomen carcass, for the same species often cluster together. This emphasizes the importance of choosing distance measures for inferences based on clustering of expressions.

optimum Log-likelihood value.

S3.1 Inference of the expected value and covariance in the EvoGeneX model – complete derivations

Specifically, let T be a rooted evolutionary tree with N terminal taxa (here species) where the branch lengths represent evolutionary time, and the root represents the last common ancestor (LCA) of all N taxa. For each specie i, the path from the root to the corresponding terminal node represents its lineage. Let T_i be the total length of lineage i and let $X_i(t)$ denote the value of the quantitative character of interest (here gene expression) for the ith lineage at time t. We assume $t = 0$ at the root of the tree. For two species i, j let $s_{i,j}$ denote the time of the speciation event when lineages i and j diverged, and consequently, $X_i(t) = X_j(t)$ for all $t < s_{i,j}$.

We assume that for each lineage i, $X_i(t)$ evolves according to a mean-reverting Ornstein-Uhlenbeck (OU) process (Hansen 1997)

$$dX_i(t) = \alpha \left[\beta_i(t) - X_i(t)\right] dt + \sigma dB_i(t) \tag{S1}$$

where $0 \le t \le T$, α is the rate of mean reversion (the strength of the pull to the optimum), σ is the volatility of the process, and the function $\beta_i(t)$ represents the dynamic optimum trait value of the OU process and as such identifies the selection regime acting on lineage i over the course of

its history. $dB_i(t)$ denotes increments of a standard Brownian motion (BM); heuristically, we can think of them as normal random variables with mean 0 and variance dt.

Multiplying both sides of (S1) by $e^{\alpha t}$ we get

$$dX_i(t)e^{\alpha t} = \alpha e^{\alpha t}\beta_i(t)dt - \alpha e^{\alpha t}X_i(t)dt + \sigma e^{\alpha t}dB_i(t)$$

or

$$dX_i(t)e^{\alpha t} + \alpha e^{\alpha t}X_i(t)dt = \alpha e^{\alpha t}\beta_i(t)dt + \sigma e^{\alpha t}dB_i(t)$$

implying

$$d(X_i(t)e^{\alpha t}) = \alpha e^{\alpha t}\beta_i(t)dt + \sigma e^{\alpha t}dB_i(t)$$

Integrating both sides from $t = 0$ to $t = t$ we get

$$X_i(t)e^{\alpha t} - X_i(0) = \int_0^t \alpha e^{\alpha s}\beta_i(s)ds + \int_0^t \sigma e^{\alpha s}dB_i(s)$$

implying

$$X_i(t) = e^{-\alpha t}X_i(0) + e^{-\alpha t}\int_0^t \alpha e^{\alpha s}\beta_i(s)ds + e^{-\alpha t}\int_0^t \sigma e^{\alpha s}dB_i(s)$$

Because this defines a Gaussian process, the first moment of $X_i(t)$ is completely specified by the deterministic (or non-stochastic) components as the stochastic component has mean 0. In particular, we have the expected trait value for species i as

$$\mathrm{E}[X_i(t) \mid X_i(0) = \theta_0] = \theta_0 e^{-\alpha t} + e^{-\alpha t}\int_0^t \alpha e^{\alpha s}\beta_i(s)ds$$

and the covariance between species i and species j is

$$\begin{aligned}
&\mathrm{Cov}[X_i(t_i), X_j(t_j) \mid X_i(0) = X_j(0) = \theta_0] \\
&= \mathrm{E}[(X_i(t_i) - E[X_i(t_i)] \mid X_i(0) = \theta_0)(X_j(t_j) - E[X_j(t_j)] \mid X_j(0) = \theta_0)] \\
&= \mathrm{E}\left[\left(e^{-\alpha t_i}\int_0^{t_i} \sigma e^{\alpha u}dB_i(u)\right)\left(e^{-\alpha t_j}\int_0^{t_j} \sigma e^{\alpha v}dB_j(v)\right)\right] \\
&= \mathrm{E}\left[\sigma^2 e^{-\alpha(t_i+t_j)}\left(\int_0^{t_i} e^{\alpha u}dB_i(u)\right)\left(\int_0^{t_j} e^{\alpha v}dB_j(v)\right)\right] \\
&= \sigma^2 e^{-\alpha(t_i+t_j)}\mathrm{E}\left[\left(\int_0^{s_{i,j}} e^{\alpha u}dB_i(u) + \int_{s_{i,j}}^{t_i} e^{\alpha u}dB_i(u)\right)\left(\int_0^{s_{i,j}} e^{\alpha v}dB_j(v) + \int_{s_{i,j}}^{t_j} e^{\alpha v}dB_j(v)\right)\right] \\
&= \sigma^2 e^{-\alpha(t_i+t_j)}\mathrm{E}\left[\begin{array}{c}
\underbrace{\left(\int_0^{s_{i,j}} e^{\alpha u}dB_i(u)\right)\left(\int_0^{s_{i,j}} e^{\alpha v}dB_j(v)\right)}_{\boldsymbol{A}} \\
+\underbrace{\left(\int_0^{s_{i,j}} e^{\alpha u}dB_i(u)\right)\left(\int_{s_{i,j}}^{t_j} e^{\alpha v}dB_j(v)\right)}_{\boldsymbol{B}} \\
+\underbrace{\left(\int_{s_{i,j}}^{t_i} e^{\alpha u}dB_i(u)\right)\left(\int_0^{s_{i,j}} e^{\alpha v}dB_j(v)\right)}_{\boldsymbol{C}} \\
+\underbrace{\left(\int_{s_{i,j}}^{t_i} e^{\alpha u}dB_i(u)\right)\left(\int_{s_{i,j}}^{t_j} e^{\alpha v}dB_j(v)\right)}_{\boldsymbol{D}}
\end{array}\right]
\end{aligned}$$

$$= \sigma^2 e^{-\alpha(t_i+t_j)} \mathrm{E}\left[\underbrace{\left(\int_0^{s_{i,j}} e^{\alpha u} dB_i(u) \right) \left(\int_0^{s_{i,j}} e^{\alpha v} dB_i(v) \right)}_{A} \right]$$ (by independent increment property of Brownian motion, each of $\boldsymbol{B}, \boldsymbol{C}, \boldsymbol{D}$ is 0 and $B_i(t) = B_j(t)$ for $t < s_{i,j}$)

$$= \sigma^2 e^{-\alpha(t_i+t_j)} \mathrm{E}\left[\int_0^{s_{i,j}} e^{2\alpha u} du \right]$$ (by Itô Isometry on $\boldsymbol{A}$)

$$= \sigma^2 e^{-\alpha(t_i+t_j)} \mathrm{E}\left[\frac{1}{2\alpha} \left(e^{2\alpha s_{i,j}} - 1 \right) \right] = \frac{\sigma^2}{2\alpha} e^{-\alpha(t_i+t_j)} (e^{2\alpha s_{i,j}} - 1) = \frac{\sigma^2}{2\alpha} e^{-\alpha(t_i+t_j-2s_{i,j})} (1 - e^{-2\alpha s_{i,j}})$$

Given multiple observations $Y_{i,k}$ for the trait value $X_i(T_i)$ for the terminal taxa i, the observed variance (including technical, environmental) could be explained by within-species variance (γ^2). Thus the trait value of kth replicate of species i is

$$Y_{i,k}(t) = X_i(t) + \varepsilon_{i,k} \tag{S2}$$

where each $\varepsilon_{i,k} \sim N(0, \gamma\sigma^2)$ is independent and identically distributed. Thus, we have the expected trait value for replicate k of species i as

$$\mathrm{E}[Y_{i,k}(t_i) \mid X_i(0) = \theta_0] = \mathrm{E}[X_i(t_i) \mid X_i(0) = \theta_0] = \theta_0 e^{-\alpha t_i} + e^{-\alpha t_i} \int_0^{t_i} \alpha e^{\alpha s} \beta_i(s) ds \tag{S3}$$

and the covariance between species i, replicate k and species j, replicate l is

$$\begin{aligned} &\mathrm{Cov}[Y_{i,k}(t_i), Y_{j,l}(t_j) \mid X_i(0) = X_j(0) = \theta_0] \\ &= \mathrm{Cov}[X_i(t_i), X_j(t_j) \mid X_i(0) = X_j(0) = \theta_0] + \begin{cases} \gamma\sigma^2 & \text{if } (i = j) \text{ and } (k = l) \\ 0 & \text{otherwise} \end{cases} \\ &= \frac{\sigma^2}{2\alpha} e^{-\alpha(t_i+t_j-2s_{i,j})} (1 - e^{-2\alpha s_{i,j}}) + \begin{cases} \gamma\sigma^2 & \text{if } (i = j) \text{ and } (k = l) \\ 0 & \text{otherwise} \end{cases} \end{aligned} \tag{S4}$$

S3.2 Different choices for the optimum-defining function $\beta_i(t)$, describe different modes of evolution – complete derivations

Equations (S3) and (S4) providing the expectation and covariance of different observations at the taxa are very general. Using different definitions of $\beta_i(t)$ leads to various biologically relevant models. The simplest model correspond to the situation where, for all i, $\beta_i(t)$ is a constant. This describes the *constrained evolution* model corresponding to one common optimum value.

Under the assumption of multiple optima, it is reasonable to assume that $\beta_i(t)$ changes at speciation events and remains constant along individual edges of the phylogenetic tree. We call the times of speciation events "epochs." The history of the ith lineage consists of a number, $\kappa(i)$, of sequential branch segments demarcated by epochs $0 = t_i^0 < t_i^1 < t_i^2 < \ldots < t_i^{\kappa(i)} = T_i$. Thus, for $t_i^{\tau-1} \leq t \leq t_i^{\tau}$ equation (S3) takes the following form

$$\begin{aligned} \mathrm{E}[Y_{i,k}(T_i) \mid X_i(0) = \theta_0] &= \theta_0 e^{-\alpha T_i} + e^{-\alpha T_i} \int_0^{T_i} \alpha e^{\alpha s} \beta_i(s) ds \\ &= \theta_0 e^{-\alpha T_i} + e^{-\alpha T_i} \sum_{\tau=1}^{\kappa(i)} \int_{t_i^{\tau-1}}^{t_i^{\tau}} \alpha e^{\alpha s} \beta_i^{\tau} ds \\ &= \theta_0 e^{-\alpha T_i} + e^{-\alpha T_i} \sum_{\tau=1}^{\kappa(i)} \left(e^{\alpha t_i^{\tau}} - e^{\alpha t_i^{\tau-1}} \right) \beta_i^{\tau} \end{aligned}$$

$$= \theta_0 e^{-\alpha T_i} + \sum_{\tau=1}^{\kappa(i)} \left(e^{-\alpha(T_i - t_i^\tau)} - e^{-\alpha(T_i - t_i^{\tau-1})} \right) \beta_i^\tau. \tag{S5}$$

The number of distinct β_i^τ might be as big as the number of tree edges $(2N-1)$. However, it is not practical to estimate so many parameters. Instead, following Hansen (1997), we assume a small number, R, of distinct optimum values θ_r, $r = 1, \ldots, R$, each corresponding to one *selective regime*. In fact one of the most interesting cases corresponds to the model with two optima where one branch of the tree follows a regime of optimum values θ_1 and the rest of the tree θ_0 (J. Chen et al. 2019; Brawand et al. 2011).

Let the binary variable $\beta_{i,r}^\tau$ represent if the τth branch on lineage i has operated in rth regime. Then we have $\beta_i^\tau = \sum_{r=1}^R \beta_{i,r}^\tau \theta_r$ and rearranging similar terms in equation (S5) gives

$$\begin{aligned} \mathrm{E}[Y_{i,k}(T_i) \mid X_i(0) = \theta_0] &= \theta_0 e^{-\alpha T_i} + \sum_{\tau=1}^{\kappa(i)} \left(e^{-\alpha(T_i - t_i^\tau)} - e^{-\alpha(T_i - t_i^{\tau-1})} \right) \sum_{r=1}^{R} \beta_{i,r}^\tau \theta_k \\ &= \theta_0 e^{-\alpha T_i} + \sum_{r=1}^{R} \left(\sum_{\tau=1}^{\kappa(i)} \left(e^{-\alpha(T_i - t_i^\tau)} - e^{-\alpha(T_i - t_i^{\tau-1})} \right) \beta_{i,r}^\tau \right) \theta_r \end{aligned} \tag{S6}$$

Note that since each branch is associated with exactly one optimum, for each i, τ there is exactly one index r such that $\beta_{i,r}^\tau = 1$ and $\beta_{i,r'}^\tau = 0$ for all $r \neq r'$. Further, self-consistency requires that $\beta_{i,r}^\tau = \beta_{j,r}^\eta$ whenever lineage i and j share the branch ending in epoch $t_i^\tau = t_j^\eta$.

The parameters $\theta_0, \theta_1, \ldots, \theta_R$, together with α, σ, γ must be estimated which we do using commonly used Maximum Likelihood (ML) estimation method.

S3.3 Maximum likelihood estimates – complete derivations

In the following, it will be convenient to make use of matrix notation. Accordingly, we collect our random variables, $X_i(t)$ for the trait values at the taxa, and $Y_{i,k}(t)$ for the replicated trait values, in the vectors $\mathbf{x}(t)$ and $\mathbf{y}(t)$, respectively, and our observed quantitative data in the vector $\mathbf{y}$ with components $y_{i+(k-1)N} = Y_{i,k}(T_i)$, the observed value of the quantitative character in each species i, $i = 1, \ldots, N$, at the end of an evolutionary process of length time $= T_i$. Thus, equation (S6) can be rewritten in matrix notation as

$$\mathrm{E}[\mathbf{y}(T) \mid \mathbf{x}(0) = \theta_0 \mathbb{1}] = \mathbf{W}\boldsymbol{\theta} \tag{S7}$$

where $\mathbb{1}$ is a vector of all 1s, column vector $\boldsymbol{\theta} = (\theta_0, \theta_1, \ldots, \theta_R)^T$ and the weight matrix W is dependent only on α among the parameters and has entries

$$\begin{aligned} W_{i+(k-1)N,0} &= e^{-\alpha T_i} \\ W_{i+(k-1)N,r} &= \sum_{\tau=1}^{\kappa(i)} \left(e^{-\alpha(T_i - t_i^\tau)} - e^{-\alpha(T_i - t_i^{\tau-1})} \right) \beta_{i,r}^\tau \end{aligned} \tag{S8}$$

for $i = 1, \ldots, N$, $k = 1, \ldots, M$ and $r = 1, \ldots, R$. Similarly, let an $MN \times MN$ matrix $\mathbf{V}$ denote the covariance matrix where the covariance between species i, replicate k and species j, replicate l is given by the entry

$$v_{(i,k),(j,l)} = v_{i+(k-1)N, j+(l-1)N} = \mathrm{Cov}[Y_{i,k}(t_i), Y_{j,l}(t_j) \mid X_i(0) = X_j(0) = \theta_0]$$

$$= \frac{\sigma^2}{2\alpha} e^{-\alpha(t_i+t_j-2s_{i,j})}(1 - e^{-2\alpha s_{i,j}}) + \begin{cases} \gamma\sigma^2 & \text{if } (i=j) \text{ and } (k=l) \\ 0 & \text{otherwise.} \end{cases} \tag{S9}$$

It is known that $\mathbf{y}$ follows a multi-variate Gaussian distribution $\mathcal{N}(\mathbf{W}\boldsymbol{\theta}, \mathbf{V})$ with mean and co-variance given by equations (S7) and (S9) (Hansen and Martins 1996). Thus, the likelihood of the parameters α, σ, γ, and $\boldsymbol{\theta}$, given the data $\mathbf{y}(T) = \mathbf{y}$, is

$$\mathcal{L}(\alpha, \sigma, \gamma, \boldsymbol{\theta} \mid \mathbf{y}) = \frac{1}{\sqrt{(2\pi)^{NM} \det \mathbf{V}}} \exp\left[-\frac{(\mathbf{y} - \mathbf{W}\boldsymbol{\theta})^T \mathbf{V}^{-1} (\mathbf{y} - \mathbf{W}\boldsymbol{\theta})}{2}\right] \tag{S10}$$

As maximizing $\mathcal{L}$ is equivalent to minimizing $U = -2\log\mathcal{L}$, we seek to minimize

$$U(\alpha, \sigma, \gamma, \boldsymbol{\theta} \mid \mathbf{y}) = NM\log(2\pi) + \log\det\mathbf{V} + (\mathbf{y} - \mathbf{W}\boldsymbol{\theta})^T \mathbf{V}^{-1} (\mathbf{y} - \mathbf{W}\boldsymbol{\theta}) \tag{S11}$$

However, it can be noted that $\mathbf{V}$ has a nice structure and can be expressed as $\sigma^2(\tilde{\mathbf{V}} + \gamma\mathbb{I})$ where $\tilde{\mathbf{V}}$ is dependent on α only among all the parameters and $\mathbb{I}$ is an identity matrix of size MN. The elements of $\tilde{\mathbf{V}}$ are given by $\tilde{V}_{(i,k),(j,l)} = \frac{1}{2\alpha} e^{-\alpha(t_i+t_j-2s_{i,j})}(1 - e^{-2\alpha s_{i,j}})$.

Thus, U can be expressed as

$$U(\alpha, \sigma, \gamma, \boldsymbol{\theta} \mid \mathbf{y}) = NM\log(2\pi\sigma^2) + \log\det(\tilde{\mathbf{V}} + \gamma\mathbb{I}) + \frac{1}{\sigma^2}(\mathbf{y} - \mathbf{W}\boldsymbol{\theta})^T (\tilde{\mathbf{V}} + \gamma\mathbb{I})^{-1} (\mathbf{y} - \mathbf{W}\boldsymbol{\theta})$$

whose minimum can be estimated using any off-the-self nonlinear optimization solver.

However, we improve the efficiency by utilizing Karush-Kuhn-Tucker conditions at the minimum solutions. Taking partial derivatives of U with respect to σ and $\boldsymbol{\theta}$ and equating them to 0 at an optimal solution $(\hat{\alpha}, \hat{\sigma}, \hat{\gamma}, \hat{\boldsymbol{\theta}})$, we get

$$\begin{aligned} \hat{\sigma}^2 &= \frac{1}{NM}(\mathbf{y} - \mathbf{W}\hat{\boldsymbol{\theta}})^T (\tilde{\mathbf{V}} + \hat{\gamma}\mathbb{I})^{-1} (\mathbf{y} - \mathbf{W}\hat{\boldsymbol{\theta}}), \text{ and} \\ \hat{\boldsymbol{\theta}} &= \left(\mathbf{W}^T (\tilde{\mathbf{V}} + \hat{\gamma}\mathbb{I})^{-1} \mathbf{W}\right)^{-1} \mathbf{W}^T (\tilde{\mathbf{V}} + \hat{\gamma}\mathbb{I})^{-1} \mathbf{y} \end{aligned} \tag{S12}$$

Thus, instead of minimizing function U of four parameters α, σ, γ and $\boldsymbol{\theta}$, it is enough to minimize a new function $\tilde{U}$ of two parameters, α and γ,

$$\tilde{U}(\alpha, \gamma) = NM\left[1 + \log 2\pi\hat{\sigma}^2(\alpha, \gamma)\right] + \log\det(\tilde{\mathbf{V}} + \gamma\mathbb{I}) \tag{S13}$$

where the following two intermediate functions

$$\begin{aligned} \hat{\boldsymbol{\theta}}(\alpha, \gamma) &= \left(\mathbf{W}^T (\tilde{\mathbf{V}} + \gamma\mathbb{I})^{-1} \mathbf{W}\right)^{-1} \mathbf{W}^T (\tilde{\mathbf{V}} + \gamma\mathbb{I})^{-1} \mathbf{y} \\ \hat{\sigma}^2(\alpha, \gamma) &= \frac{1}{NM}(\mathbf{y} - \mathbf{W}\hat{\boldsymbol{\theta}}(\alpha, \gamma))^T (\tilde{\mathbf{V}} + \gamma\mathbb{I})^{-1} (\mathbf{y} - \mathbf{W}\hat{\boldsymbol{\theta}}(\alpha, \gamma)) \end{aligned} \tag{S14}$$

give the values of the remaining two parameters σ and $\boldsymbol{\theta}$ at the optimal solution.

S3.4 ML estimates for Brownian model – complete derivations

We need to compute maximum likelihood estimate for the Brownian model as well for comparing with the EvoGeneX model of evolution. BM is a simplified model in comparison to OU: there is no "attracting" optimal values and hence there is no α parameter and θ has only one value to be estimated corresponding to θ_0.

Starting with the stochastic equation $Y_{i,k}(t) = X_i(t) + \varepsilon_{i,k} \sim N(0, \gamma\sigma^2)$ and the stochastic differential equation $X_i(t) = \sigma dB_i(t)$ we can follow the steps in the previous sections to derive the following log likelihood function $U = -2\log\mathcal{L}$

$$U(\sigma, \gamma, \theta_0 \mid \mathbf{y}) = NM\log(2\pi\sigma^2) + \log\det(\tilde{\mathbf{V}} + \gamma\mathbb{I}) + \frac{1}{\sigma^2}(\mathbf{y} - \theta_0\mathbb{1})^T(\tilde{\mathbf{V}} + \gamma\mathbb{I})^{-1}(\mathbf{y} - \theta_0\mathbb{1}) \quad \text{(S15)}$$

where the elements of $\tilde{\mathbf{V}}$ are given by $\tilde{V}_{(i,k),(j,l)} = s_{i,j}$, the time from the root of the phylogenetic tree to the least common ancestor of the taxa i, j. Intuitively, under Brownian motion, changes in trait values over any interval of time t are always drawn from a normal distribution with mean 0 and variance proportional to the product of the rate of evolution and the length of time ($\sigma^2 t$).

Using Karush-Kuhn-Tucker conditions at the minimum solutions, we can show that it is enough to solve a function $\tilde{U}(\gamma)$ of one parameter γ

$$\tilde{U}(\gamma) = NM\left[1 + \log 2\pi\hat{\sigma}^2(\gamma)\right] + \log\det(\tilde{\mathbf{V}} + \gamma\mathbb{I}) \quad \text{(S16)}$$

where the following two intermediate functions

$$\begin{aligned} \hat{\theta}_0(\gamma) &= \left(\mathbb{1}^T(\tilde{\mathbf{V}} + \gamma\mathbb{I})^{-1}\mathbb{1}\right)^{-1}\mathbb{1}^T(\tilde{\mathbf{V}} + \gamma\mathbb{I})^{-1}\mathbf{y} \\ \hat{\sigma}^2(\alpha, \gamma) &= \frac{1}{NM}(\mathbf{y} - \hat{\theta}_0(\gamma)\mathbb{1})^T(\tilde{\mathbf{V}} + \gamma\mathbb{I})^{-1}(\mathbf{y} - \hat{\theta}_0(\gamma)\mathbb{1}) \end{aligned} \quad \text{(S17)}$$

give the values of the remaining two parameters σ and θ_0 at the optimal solution.

S3.5 Computing statistical significance

We use statistical hypothesis testing to decide which of the three different modes of evolution the trait has undergone: i) neutral, ii) constrained and ii) adaptive (see Section 2.1 in main text). For this purpose we use likelihood ratio test. Specifically, for two models H_0, H_1 with parameters $\mathbf{\Theta}_0, \mathbf{\Theta}_1$ and likelihoods $L_0(\mathbf{\Theta}_0), L_1(\mathbf{\Theta}_1)$, to test if H_1 models the data better than H_0, we check if the ratio of likelihoods, $\lambda = L_1/L_0$ is significantly greater than 1. By a result of Wilks (1938), the test statistic $-2\log(\lambda)$ is asymptotically χ^2 distributed degrees of freedom equal to the difference in dimensionality of $\mathbf{\Theta}_1$ and $\mathbf{\Theta}_0$. Thus the statistical significance is provided by the upper tail probability $\mathrm{P}[x > -2\log(\lambda)]$ of χ^2 distribution.

S4 Simulations

In addition to the 9-species phylogenetic tree in Section S1.1 and Figure S1, which we call *dros9study* in this section, we simulated gene expression on 5 more phylogenetic trees:

1. *dros9strch5*: obtained by multiplying 5 to each branch length in *dros9study*.

2. *dros9strch10*: obtained by multiplying 10 to each branch length in *dros9study*.

3. *dros48*: 48 species *Drosophila* species tree obtained from Longdon (2015) shown in Figures S8 and S9.

4. *dros35*: 35 species *Drosophila* species tree (Figure S10) obtained from *dros48* by removing 13 randomly picked species: *D. neonasuta, D. neohypocausta, D. ruberrima, D. acutilabella, D. tripunctata, D. guarani, D. orientacea, D. angularis, D. unispina, D. falleni, D. tenebrosa, D. recens, D. macrospina* using `drop.tip` function of `ape` R package.

```
((D._grimshawi:0.09031,(D._hydei:0.10492,D._virilis:0.07623)1.00:0.02129):0.03754,
((((((D._albomicans:0.00397,D._nasuta:0.00388)0.86:0.00095,D._neonasuta:0.00580)
1.00:0.00827,D._pallidifrons:0.01555)1.00:0.04497,((D._hypocausta:0.02537,
D._siamana:0.02608)1.00:0.05639,D._neohypocausta:0.04916)0.89:0.00764)0.95:0.00649,
((D._formosana:0.00351,D._immigrans:0.00305)1.00:0.05115,D._ruberrima:0.05220)
0.89:0.00769)1.00:0.03628,((((((D._acutilabella:0.05255,D._tripunctata:0.05334)
1.00:0.02205,((((((D._arawakana:0.00390,D._nigrodunni:0.00313)1.00:0.01123,
(D._dunni:0.00049,D._similis:0.00135)1.00:0.01822)1.00:0.01104,D._polymorpha:0.02993)
0.85:0.00341,(D._cardinoides:0.00934,D._parthenogenetica:0.01121)1.00:0.02051)
1.00:0.02062,D._cardini:0.02609)1.00:0.03583,D._guarani:0.06651)0.92:0.00810)
1.00:0.03185,(D._bizonata:0.03998,((D._orientacea:0.00142,D._testacea:0.00097)
1.00:0.02680,D._putrida:0.03330)1.00:0.01114)1.00:0.02682)0.95:0.00590,
(((D._angularis:0.04271,(((D._brachynephros:0.02520,(D._curvispina:0.01053,
D._unispina:0.02066)1.00:0.00591)1.00:0.00678,D._phalerata:0.02903)1.00:0.00709,
(D._falleni:0.01882,D._innubila:0.01740)1.00:0.01632)1.00:0.00911)1.00:0.01825,
((((D._deflecta:0.01760,D._subpalustris:0.01749)1.00:0.01216,D._guttifera:0.04534)
0.88:0.00414,((D._quinaria:0.02305,D._tenebrosa:0.01599)0.74:0.00364,
(D._recens:0.01566,D._transversa:0.01026)1.00:0.00497)1.00:0.00883)0.99:0.00541,
D._nigromaculata:0.02585)1.00:0.03327)1.00:0.00935,D._histrio:0.06232)
1.00:0.00869)0.85:0.00441,((D._funebris:0.03090,D._multispina:0.03430)
1.00:0.01564,D._macrospina:0.06776)1.00:0.02986)1.00:0.01626,
D._sternopleuralis:0.08210)1.00:0.01330):0.00198)1.00;
```

Figure S8: **Newick tree for 48 *Drosophila* species.**

5. *dros22*: 35 species *Drosophila* species tree (Figure S11) obtained from *dros35* by removing 13 randomly picked species: *D. albomicans, D. nasuta, D. pallidifrons, D. hypocausta, D. siamana, D. formosana, D. immigrans, D. bizonata, D. testacea, D. brachynephros, D. curvispina, D. phalerata, D. innubila.*

Both single-regime and two-regime scenarios were simulated in all 6 trees. For the three trees with 9 species (*dros9study, dros9strch5, dros9strch10*) the two regimes correspond to *Drosophila* and *Sophophora* subgenera as shown in Figure 1a. For the three bigger trees (*dros22, dros35, dros48*), a group of species including *D. nigromaculata* was kept in one regime and the rest in the second regime as shown in Figures. S9, S10, S11.

We simulated expression values for 1000 genes using the stochastic differential equations (2) and (3) governing our OU-based model of gene expression evolution on phylogenetic trees, for each setting of the parameters α, σ, β and γ subject to biologically realistic constraints discussed below.

In all of our simulations we set the expression level at the root (the most recent common ancestor of all the species) at $\beta_i(0) = 1000$. We vary parameters as follows: $\sigma^2 \in \{1, 2, 5, 10, 20, 50, 100\}$; $\gamma \in \{1, 1/2, 1/4, 1/8, 1/16, 1/32, 1/64\}$; $\alpha \in \{1/8, 1/4, 1/2, 1, 2, 4, 8, 16, 32\}$. We simulated 4 replicated expression values for 1000 genes in each of the three different scenarios: BM and OU single-regime (OU1) on all 6 trees, and OU two-regime (OU2) on all trees except *dros9strch5* as we expected the results to be somewhat in between those for *dros9strch10* and *dros9study*. For two-regime, we varied the ratio of mean expression for the two regimes, we call this ratio as $fold \in \{0.5, 0.8, 0.9, 0.95, 1.05, 1.1, 1.25, 2\}$. Additionally, to experiment with the number of replicates, we simulated single-regime expression for 1000 genes for $K \in \{6, 8, 10\}$ replicates for a limited

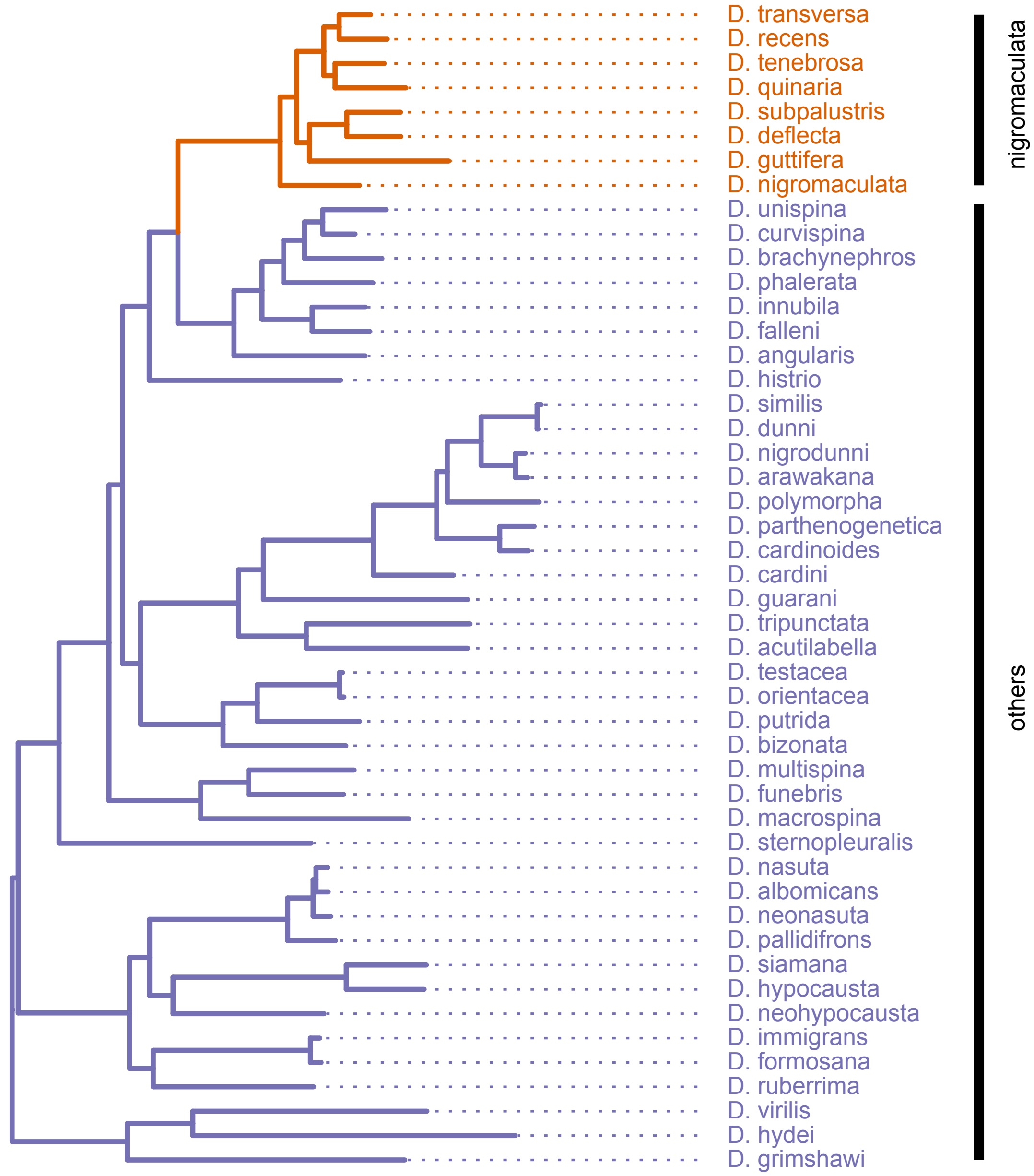

Figure S9: ***dros48*: Phylogenetic tree with 48 *Drosophila* species used for simulations.** Both single-regime and two-regime simulations were done. In two-regime, the groups of species named nigromaculata was in one regime and others were in the second regime.

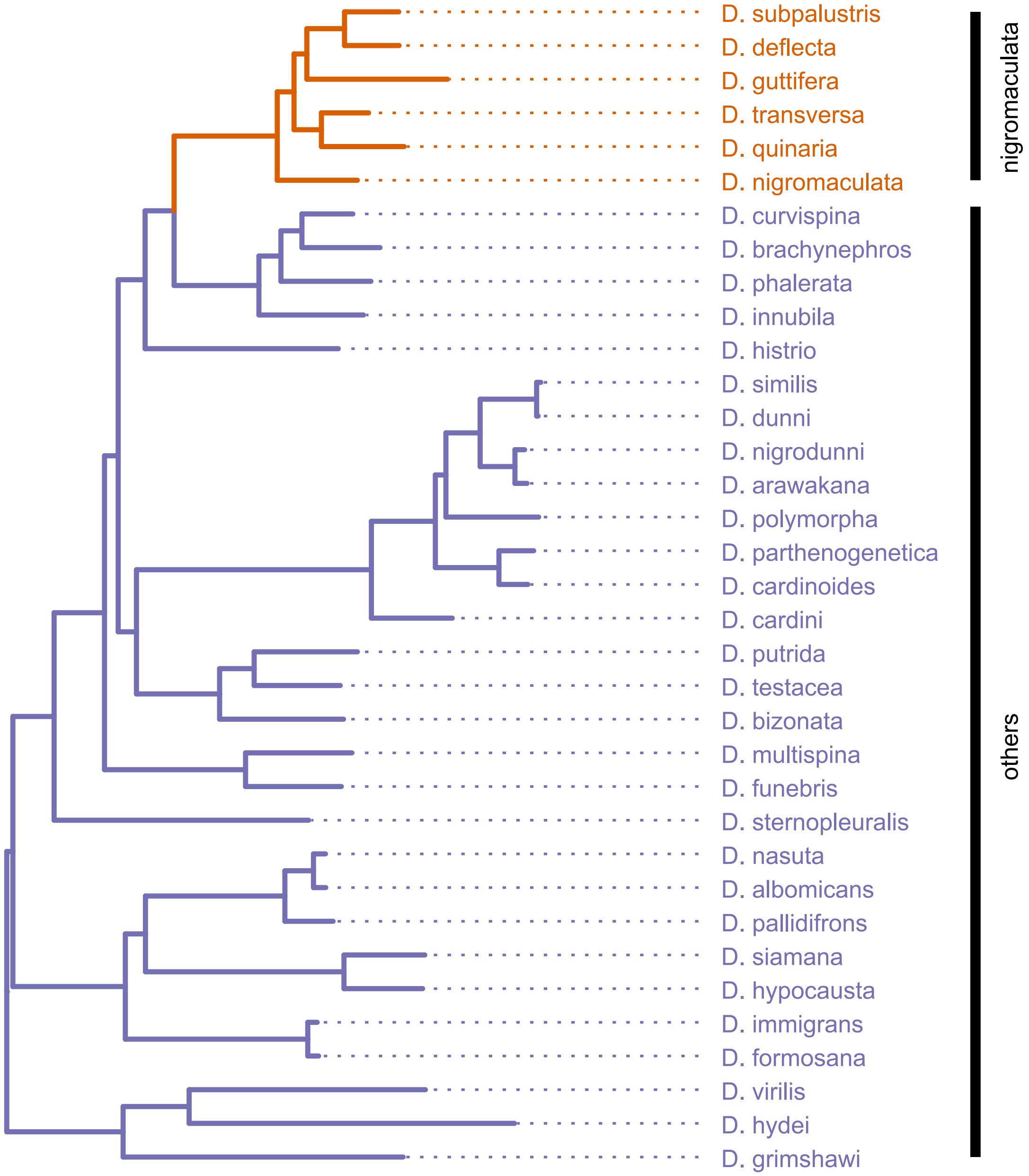

Figure S10: ***dros35*: Phylogenetic tree with 35 *Drosophila* species used for simulations.** Both single-regime and two-regime simulations were done. In two-regime, the groups of species named nigromaculata was in one regime and others were in the second regime.

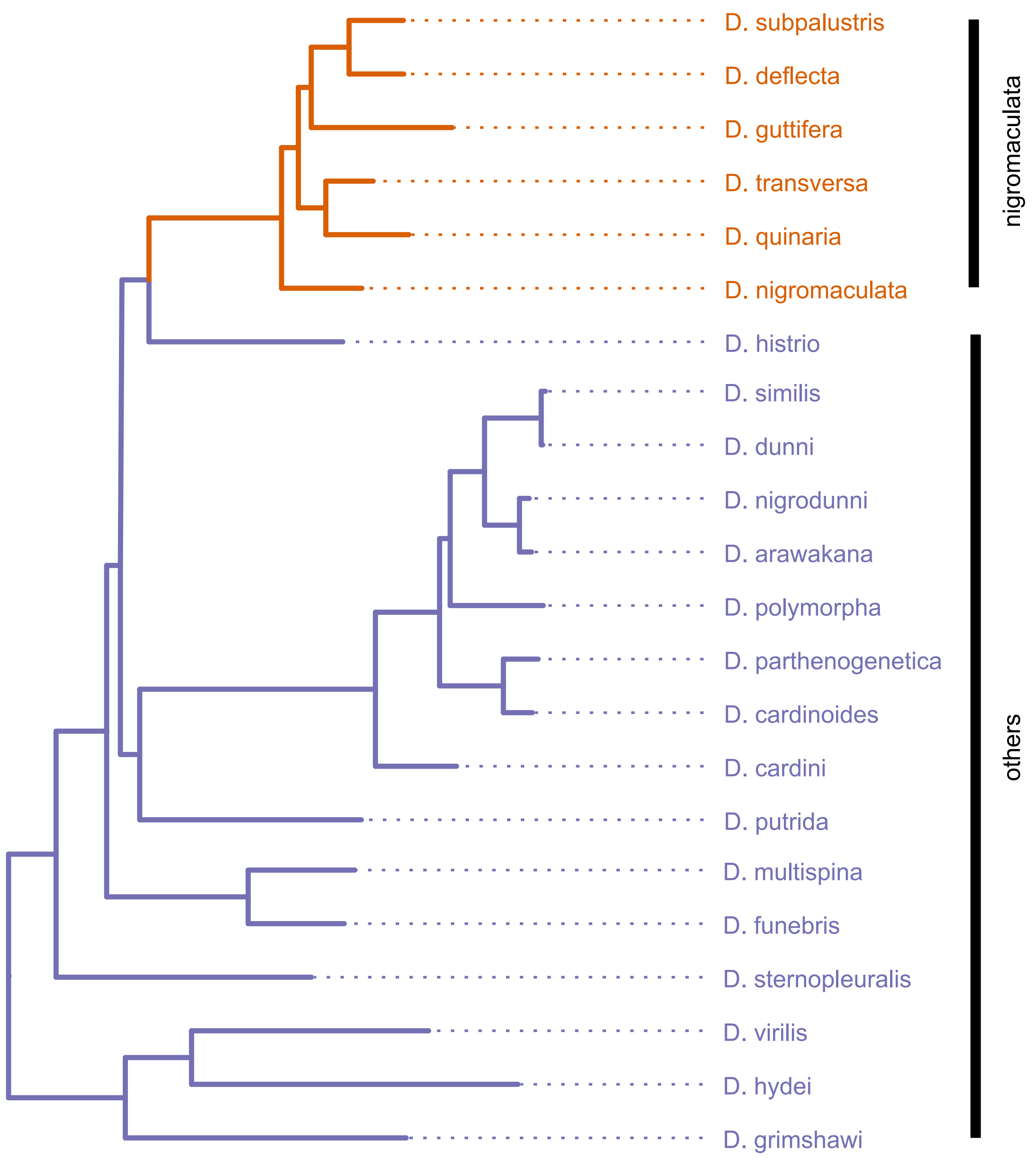

Figure S11: ***dros22*: Phylogenetic tree with 22 *Drosophila* species used for simulations.** Both single-regime and two-regime simulations were done. In two-regime, the groups of species named nigromaculata was in one regime and others were in the second regime.

sets of configurations of $\alpha \in \{1/8, 1/4, 1/2, 1, 2\}$ and $fold \in \{1.05, 1.1, 1.25\}$.

Under each of the parameter settings, we compare the effectiveness of the two algorithms, OUCH.AV (King and Butler 2009) that uses mean of replicates and our EvoGeneX. We also consider OUCH.MV, a version of OUCH (King and Butler 2009) that treats replicated expression values as multivariate trait whose components co-evolve, on genes simulated on *dros8study* with 4 replicates. The effectiveness of the algorithms are measured in terms of area under precision recall curve (auPRC_R) at cutoff values $R \in 0.01, 0.05, 0.1, 0.25, 0.5, 1$ while treating the algorithms as a classifier that separates OU from BM in single-regime and OU2 from OU1 and BM in the two-regime.

S4.1 Single-regime simulation results

Figures S12 through S17 show the gain in $\text{auPRC}_{0.5}$ by EvoGeneX over OUCH.AV for all 6 trees with 4 replicates in single-regime.

As seen in Figures S12 through S17, the cases corresponding to $\alpha\gamma > 0.5$ are contrary to our assumption that within-species variation is greater than between-species variation. So, in all subsequent results we only consider the parameter settings where $\alpha\gamma \leq 0.5$.

Figure S18 extends Figure 2a on *dros9study* and Figures S19 through S23 shows the similar results on the rest of the 5 trees.

Figure S24 compare the performance of the algorithms when the trees are varied while using the same number (4) of replicates and Figure S25 compare the performance of the algorithms when number of replicates used are varied while keeping the tree same (*dros9study*).

S4.2 Two-regime simulation results

Figure S26 extends Figure 2 by also incorporating the performance metric for fold values less than 1. It shows that the performance of EvoGeneX is not affected if the optimum expression for a branch goes down instead of going up by the same amount of fold change.

S4.3 Time taken by EvoGeneX

For each parameter setting and for each simulated gene, we captured the time and memory taken by the methods using R package `peakRAM`. The memory usage reported for all the methods were same as the starting matrix of expression values were same. We report the time taken by the algorithms in Figures S32, S33 and S34.

S5 Genes with neutral expression reconstruct expression based phylogenetic tree close to the sequence based tree

We reconstructed the phylogenetic tree from the expression values of all genes across species for each body-part and sex separately using the neighbour joining (NJ) method and the Spearman's correlation based distances. We repeated the same considering only the genes whose expression evolution could not be rejected as neutral by EvoGeneX. The Robinson-Foulds distances of the reconstructed trees from the known sequence based tree (Section S1.1) are shown in Table S2. It can be noted that the trees constructed using only the genes undergoing neutral (or nearly neutral) expression evolution are often closer to the sequence based evolutionary tree than the tree constructed using all genes.

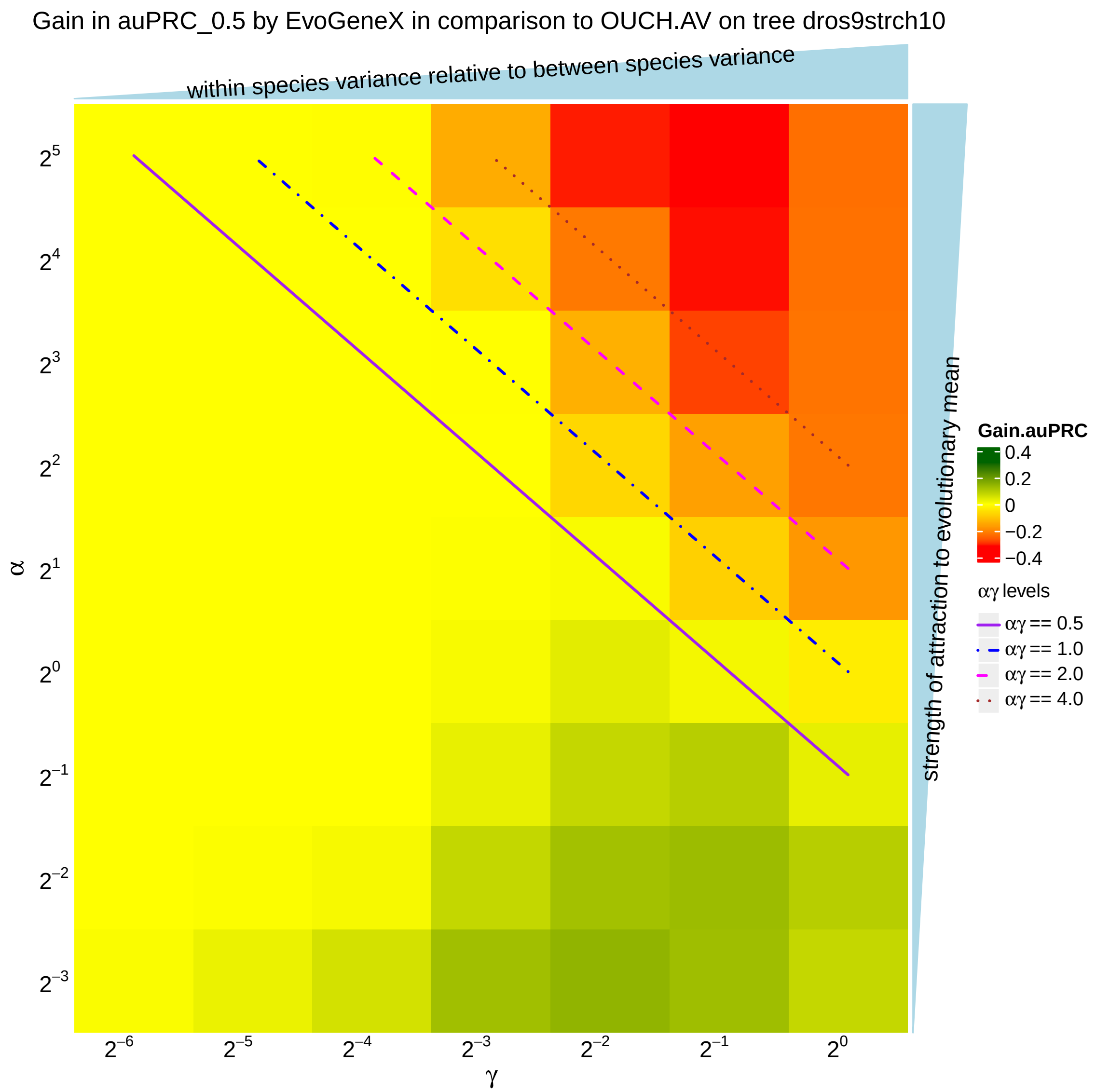

Figure S12: **Gain in auPRC$_{0.5}$ by EvoGeneX in comparison to OUCH.AV on tree *dros9strch10* with 4 replicates in single-regime.** For each combinations of α and γ the genes simulated for all values of $\sigma^2 \in \{1, 2, 5, 10, 20, 50, 100\}$ are aggregated while computing auPRC. The cases $\alpha\gamma > 0.5$ correspond to the cases where the within-species variation is greater than between-species variation, contrary to our assumption. In such cases averaging gives better result.

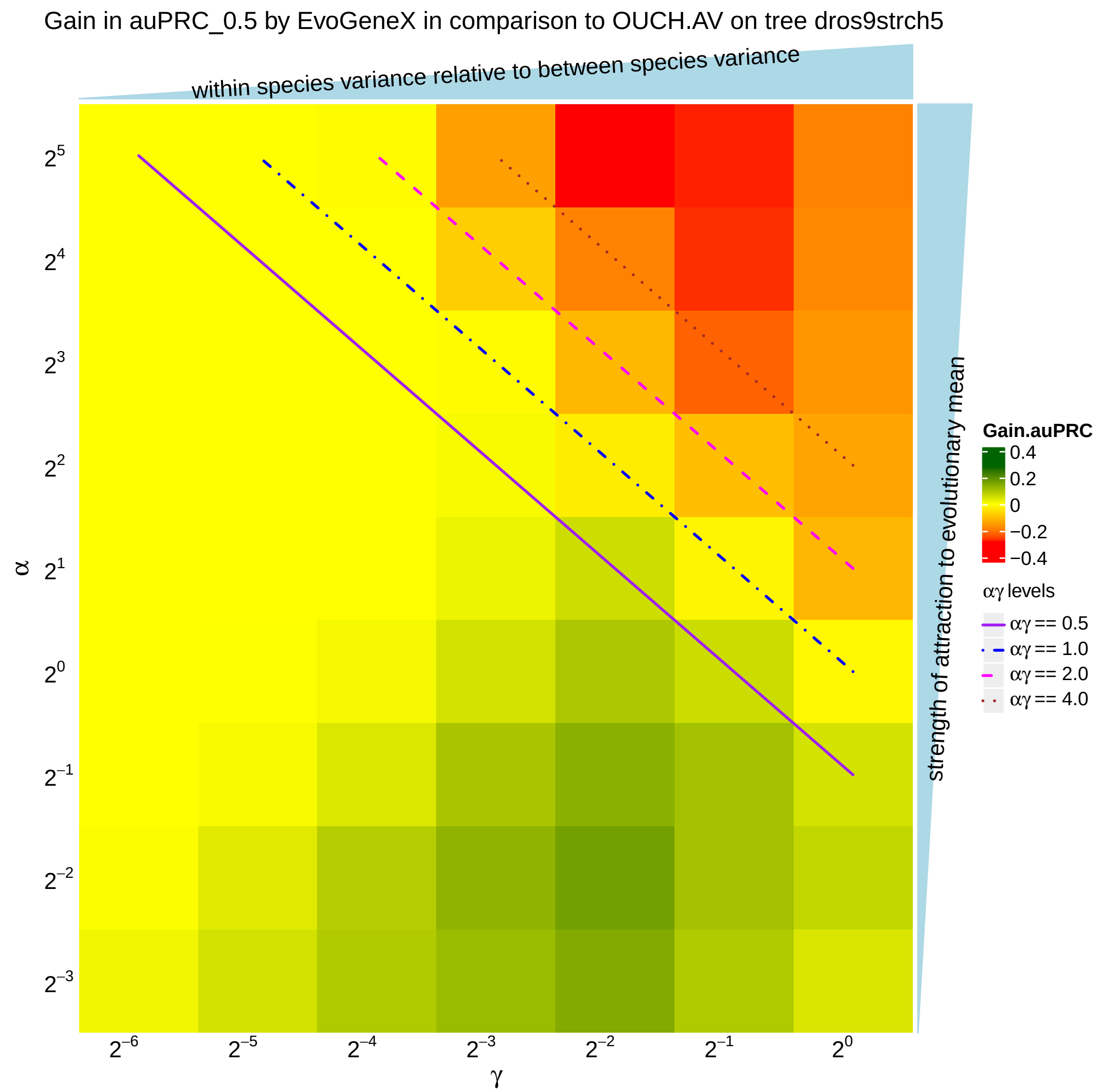

Figure S13: **Gain in auPRC$_{0.5}$ by EvoGeneX in comparison to OUCH.AV on tree *dros9strch5* with 4 replicates in single-regime.** For each combinations of α and γ the genes simulated for all values of $\sigma^2 \in \{1, 2, 5, 10, 20, 50, 100\}$ are aggregated while computing auPRC. The cases $\alpha\gamma > 0.5$ correspond to the cases where the within-species variation is greater than between-species variation, contrary to our assumption. In such cases averaging gives better result.

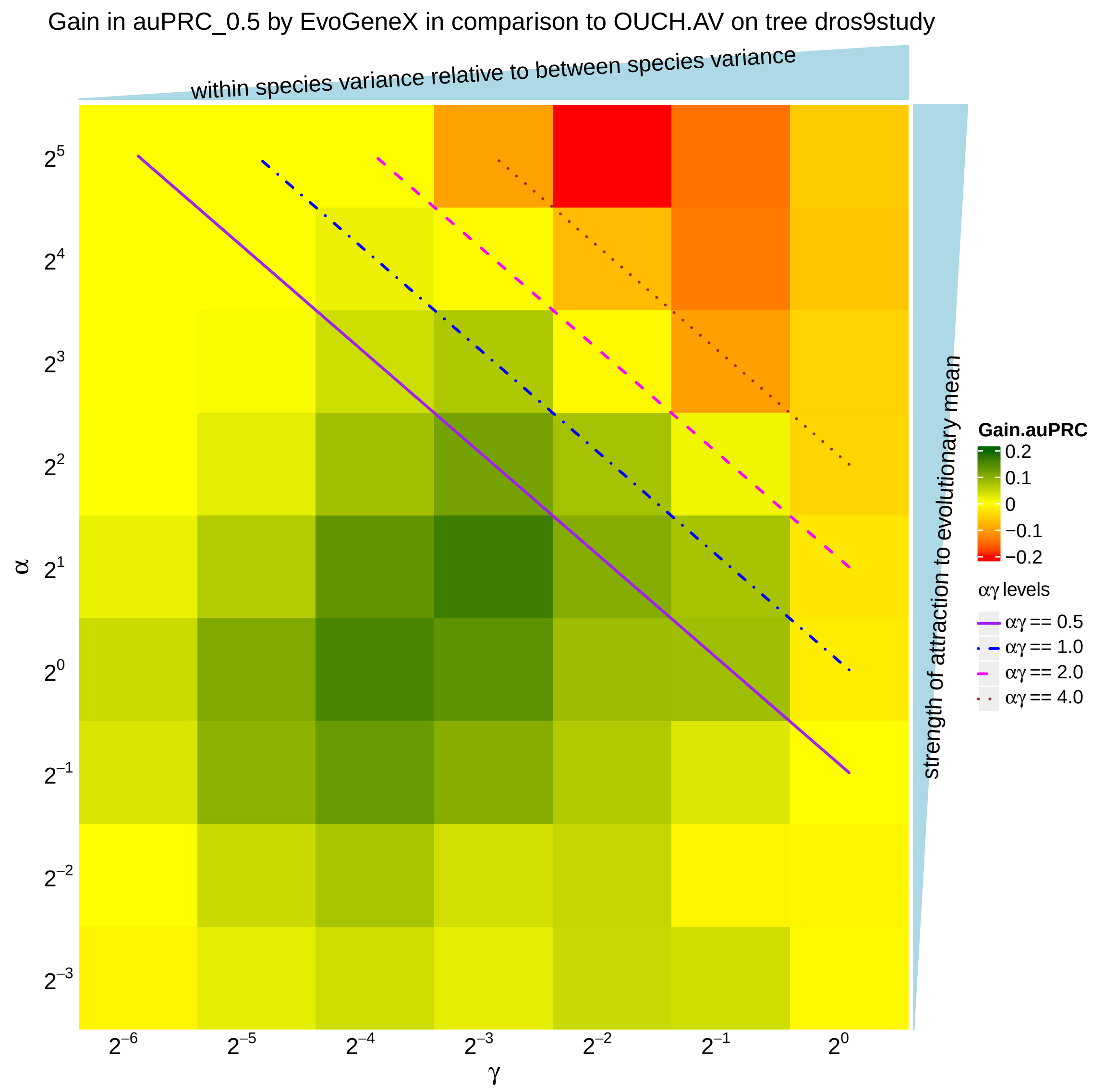

Figure S14: **Gain in auPRC$_{0.5}$ by EvoGeneX in comparison to OUCH.AV on tree *dros9study* with 4 replicates in single-regime.** For each combinations of α and γ the genes simulated for all values of $\sigma^2 \in \{1, 2, 5, 10, 20, 50, 100\}$ are aggregated while computing auPRC. The cases $\alpha\gamma > 0.5$ correspond to the cases where the within-species variation is greater than between-species variation, contrary to our assumption. In such cases averaging gives better result. However, as the branch lengths reduces implying that the time since root reduces, the advantage of averaging reduces.

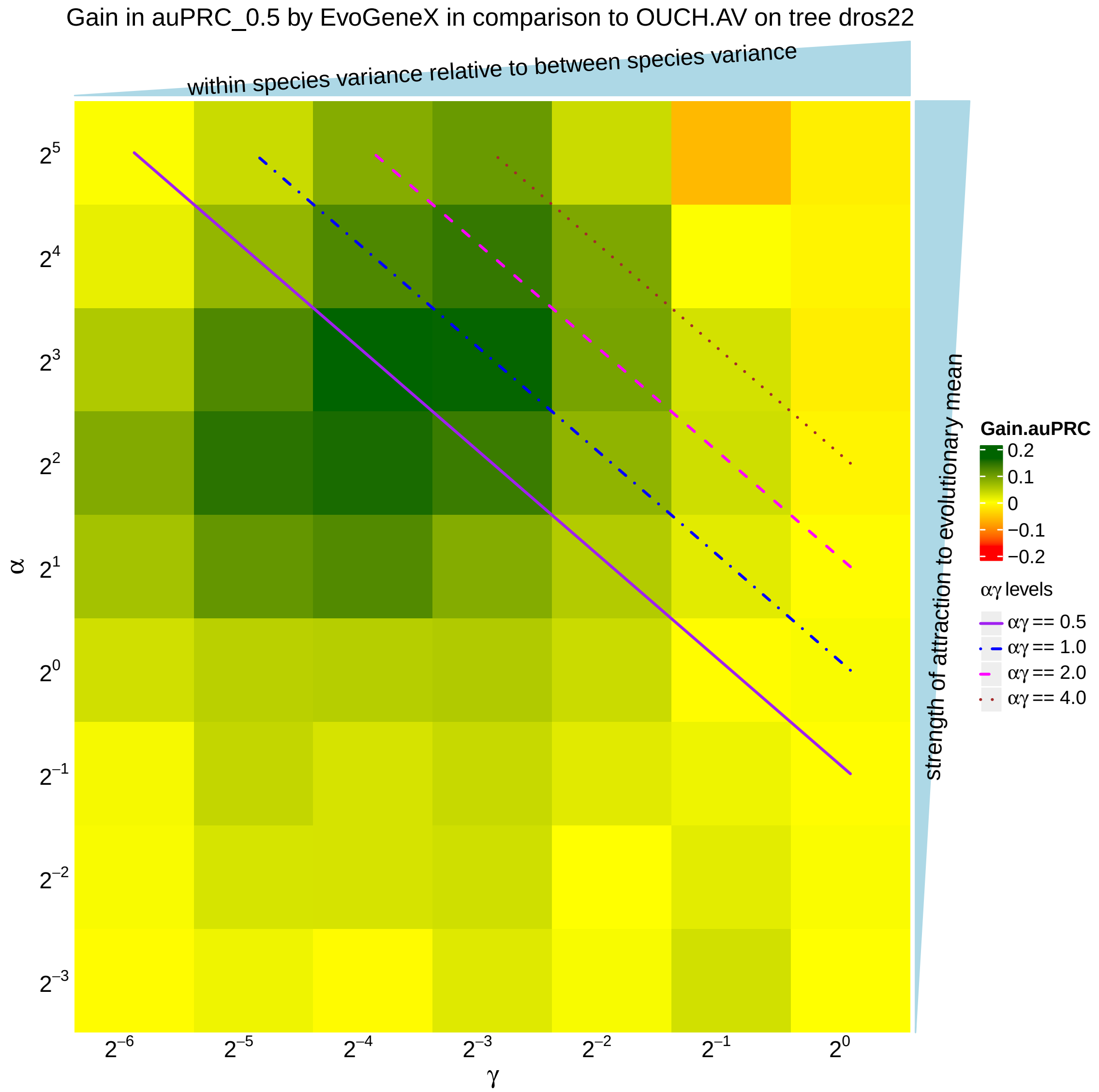

Figure S15: **Gain in auPRC$_{0.5}$ by EvoGeneX in comparison to OUCH.AV on tree *dros22* with 4 replicates in single-regime.** For each combinations of α and γ the genes simulated for all values of $\sigma^2 \in \{1, 2, 5, 10, 20, 50, 100\}$ are aggregated while computing auPRC. The cases $\alpha\gamma > 0.5$ correspond to the cases where the within-species variation is greater than between-species variation, contrary to our assumption. In such cases averaging is expected to give better result. However, we notice that as the number of species in the tree increases even our approach does better.

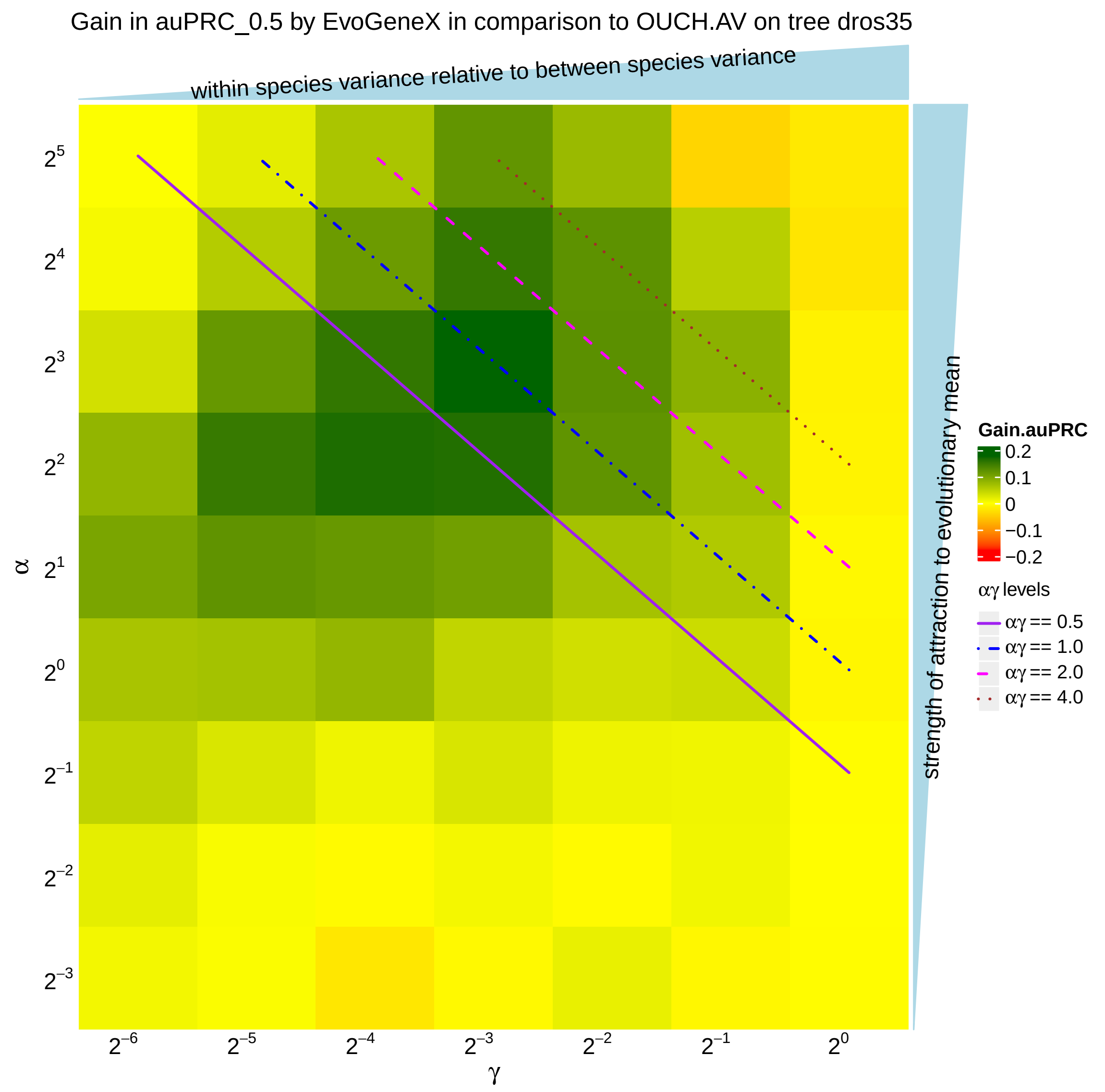

Figure S16: **Gain in auPRC$_{0.5}$ by EvoGeneX in comparison to OUCH.AV on tree *dros35* with 4 replicates in single-regime.** For each combinations of α and γ the genes simulated for all values of $\sigma^2 \in \{1, 2, 5, 10, 20, 50, 100\}$ are aggregated while computing auPRC. The cases $\alpha\gamma > 0.5$ correspond to the cases where the within-species variation is greater than between-species variation, contrary to our assumption. In such cases averaging is expected to give better result. However, we notice that as the number of species in the tree increases even our approach does better.

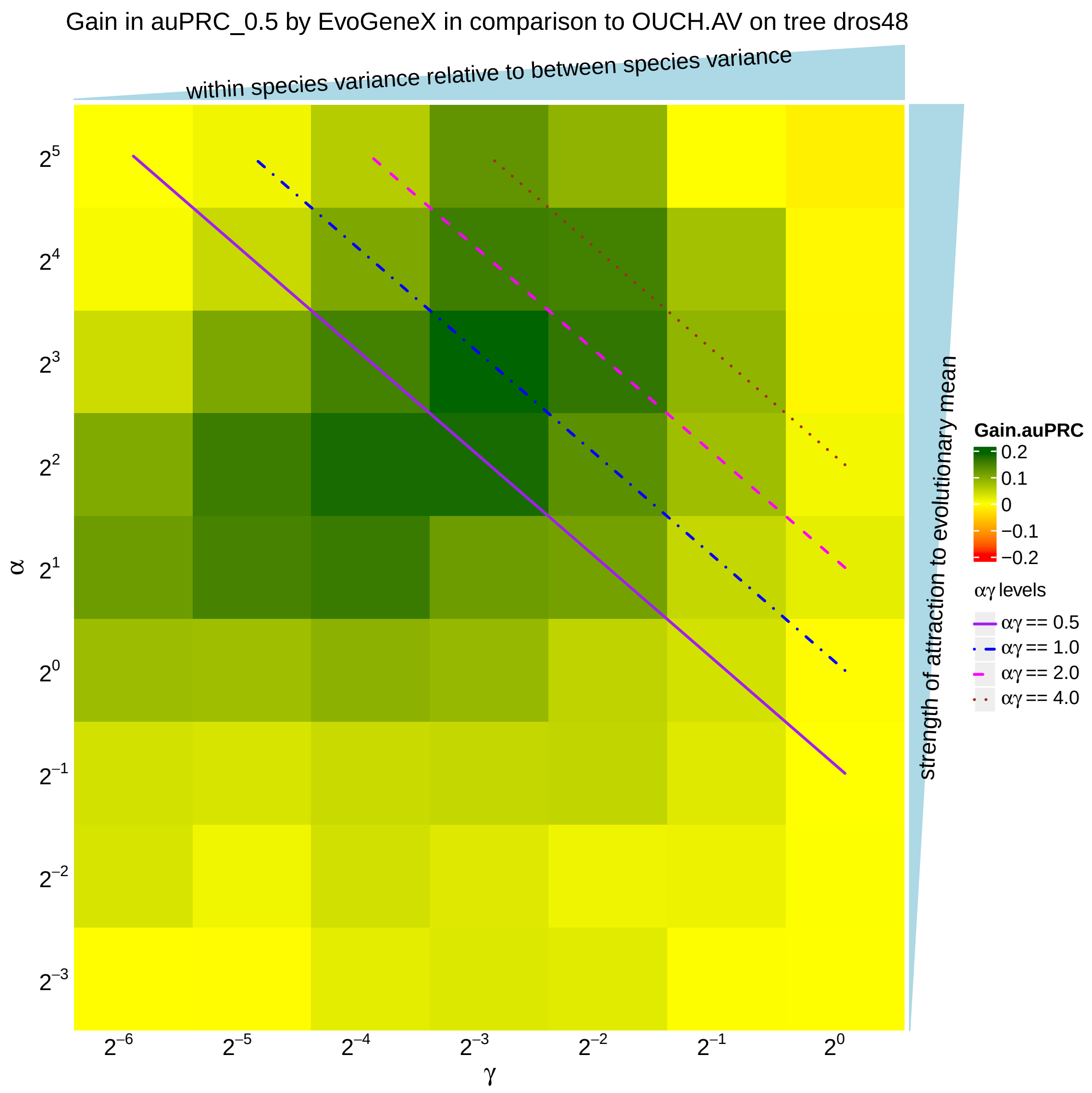

Figure S17: **Gain in auPRC$_{0.5}$ by EvoGeneX in comparison to OUCH.AV on tree *dros48* with 4 replicates in single-regime.** For each combinations of α and γ the genes simulated for all values of $\sigma^2 \in \{1, 2, 5, 10, 20, 50, 100\}$ are aggregated while computing auPRC. The cases $\alpha\gamma > 0.5$ correspond to the cases where the within-species variation is greater than between-species variation, contrary to our assumption. In such cases averaging is expected to give better result. However, we notice that as the number of species in the tree increases even our approach does better.

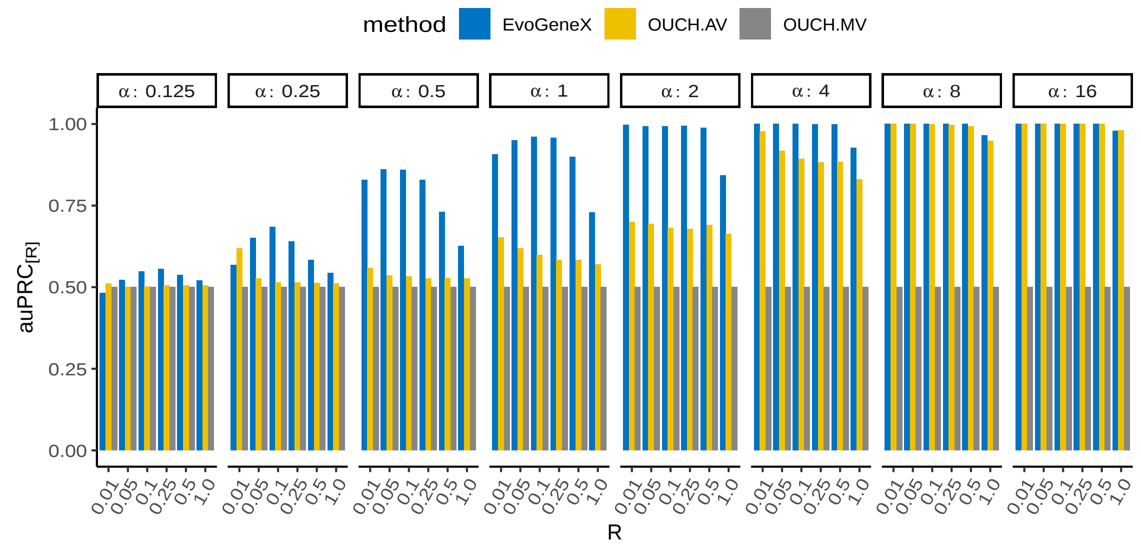

Figure S18: **Detailed results on single-regime simulations on *dros9study* using 4 replicates.** For each α the genes simulated for all values of $\sigma^2 \in \{1, 2, 5, 10, 20, 50, 100\}$ and $\gamma \in \{1, 1/2, 1/4, 1/8, 1/16, 1/32, 1/64\}$ under the condition $\alpha\gamma \leq 0.5$ representing the cases where within-species variation does not exceed the between-species variation are aggregated while computing auPRC. When α is bigger, the attraction towards mean level in OU is stronger and it becomes easier for both the methods to differentiate between constrained and neutral evolution and they perform similarly. For lower α EvoGeneX performs better by utilizing replicated values. It seems that OUCH.MV (experimented with default parameters) performs similar to a random method and hence it was not experimented on other trees.

Table S2: **Robinson-Foulds distance of the known sequence based tree (Section S1.1) from the Neighbour Joining tree obtained by using Spearman's correlation based distance of the expression values considering three gene groups.** (i) all genes, (ii) neutrally evolved of genes as detected by EvoGeneX, and (iii) constrained genes.

Body part	Genes<br>Sex	All	Neutral	Constrained
**Head**	**Female**	0	0	6
	**Male**	0	2	8
**Thorax**	**Female**	2	2	10
	**Male**	2	2	8
**Viscera**	**Female**	2	2	4
	**Male**	2	0	4
**Abdomen**	**Female**	2	0	6
	**Male**	0	0	4
**Gonad**	**Female**	2	0	4
	**Male**	0	0	8

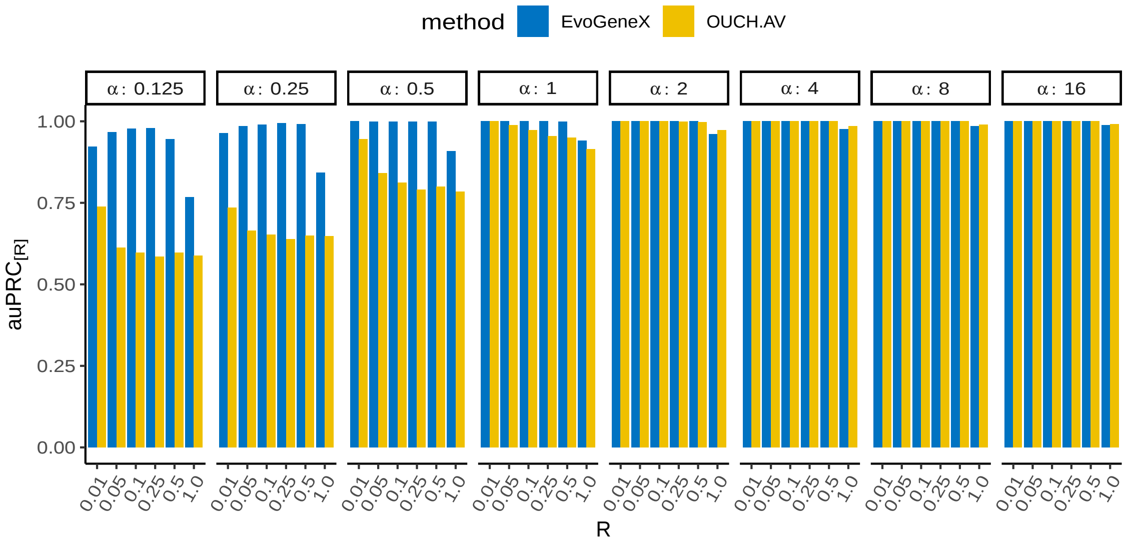

Figure S19: **Detailed results on single-regime simulations on *dros9strch10* using 4 replicates.** For each α the genes simulated for all values of $\sigma^2 \in \{1, 2, 5, 10, 20, 50, 100\}$ and $\gamma \in \{1, 1/2, 1/4, 1/8, 1/16, 1/32, 1/64\}$ under the condition $\alpha\gamma \leq 0.5$ representing the cases where within-species variation does not exceed the between-species variation are aggregated while computing auPRC. As seen in Figures S18, S20 and S19 increasing branch length while keeping α fixed also makes attraction towards mean level in OU stronger (note that α and t appear together in the exponent in the stochastic differential equation) and the two algorithms can easily differentiate between the neutral and constrained mode of evolution. For lower α EvoGeneX performs better by utilizing replicated values.

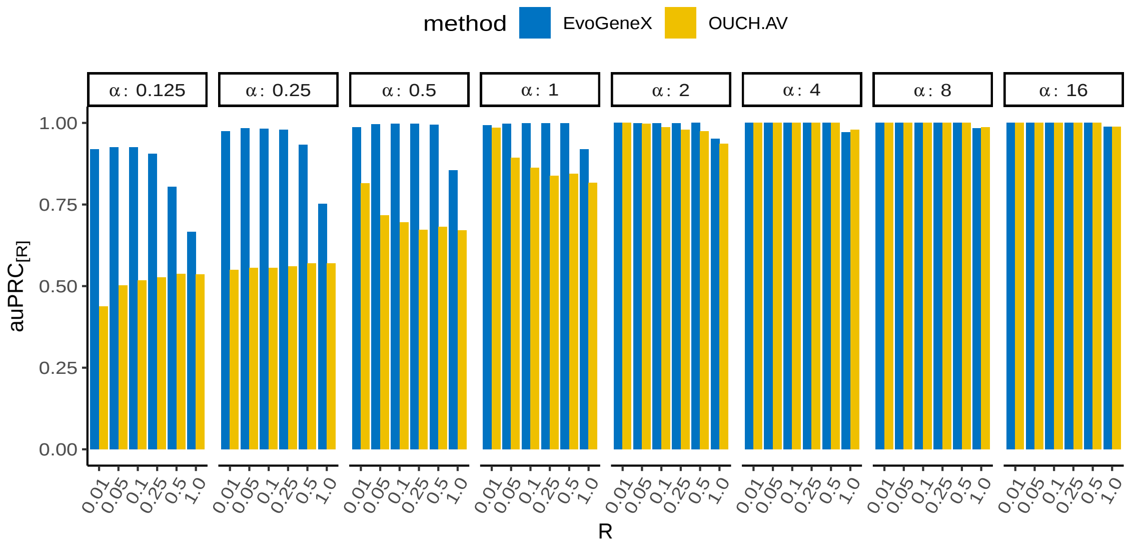

Figure S20: **Detailed results on single-regime simulations on *dros9strch5* using 4 replicates.** For each α the genes simulated for all values of $\sigma^2 \in \{1, 2, 5, 10, 20, 50, 100\}$ and $\gamma \in \{1, 1/2, 1/4, 1/8, 1/16, 1/32, 1/64\}$ under the condition $\alpha\gamma \leq 0.5$ representing the cases where within-species variation does not exceed the between-species variation are aggregated while computing auPRC. As seen in Figures S18, S20 and S19 increasing branch length while keeping α fixed also makes attraction towards mean level in OU stronger (note that α and t appear together in the exponent in the stochastic differential equation) and the two algorithms can easily differentiate between the neutral and constrained mode of evolution. For lower α EvoGeneX performs better by utilizing replicated values.

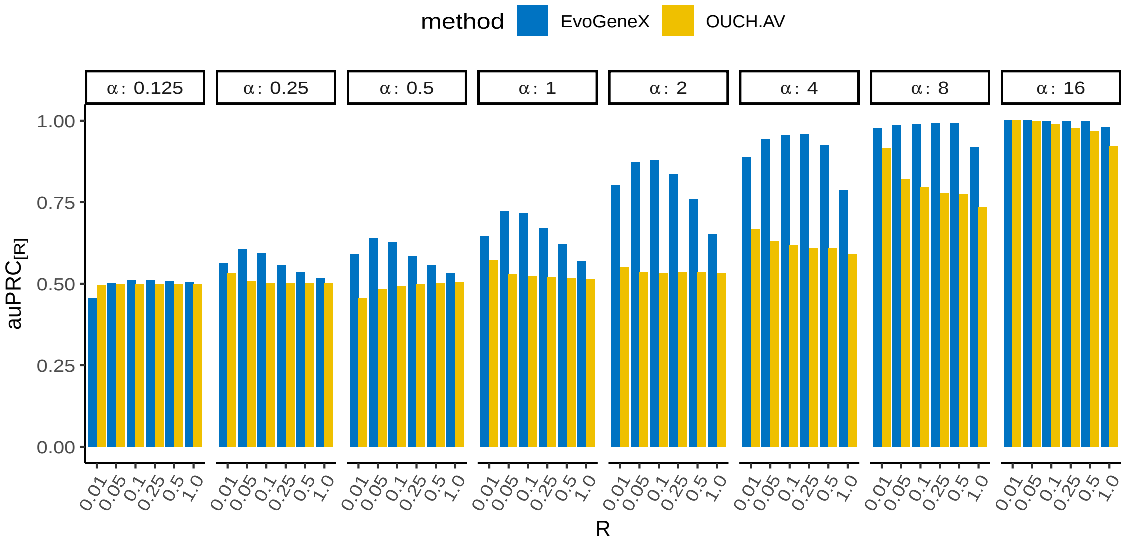

Figure S21: **Detailed results on single-regime simulations on *dros22* using 4 replicates.** For each α the genes simulated for all values of $\sigma^2 \in \{1, 2, 5, 10, 20, 50, 100\}$ and $\gamma \in \{1, 1/2, 1/4, 1/8, 1/16, 1/32, 1/64\}$ under the condition $\alpha\gamma \leq 0.5$ representing the cases where within-species variation does not exceed the between-species variation are aggregated while computing auPRC. As seen in Figures S21, S22 and S23, unless α is too high, increasing number of species while keeping α and branch length fixed, EvoGeneX performs better by utilizing replicated expression values.

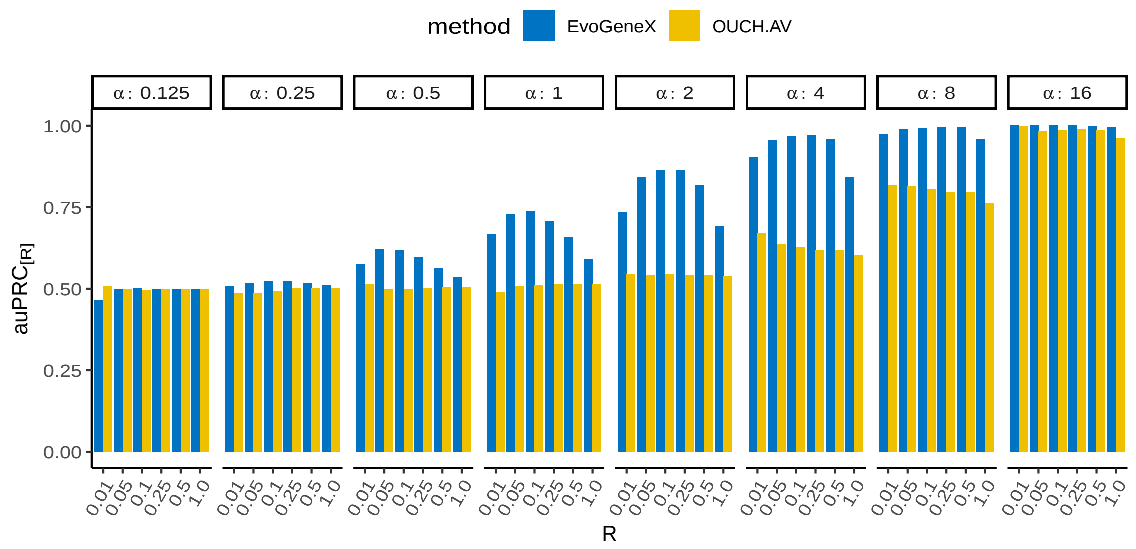

Figure S22: **Detailed results on single-regime simulations on *dros35* using 4 replicates.** For each α the genes simulated for all values of $\sigma^2 \in \{1, 2, 5, 10, 20, 50, 100\}$ and $\gamma \in \{1, 1/2, 1/4, 1/8, 1/16, 1/32, 1/64\}$ under the condition $\alpha\gamma \leq 0.5$ representing the cases where within-species variation does not exceed the between-species variation are aggregated while computing auPRC. As seen in Figures S21, S22 and S23, unless α is too high, increasing number of species while keeping α and branch length fixed, EvoGeneX performs better by utilizing replicated expression values.

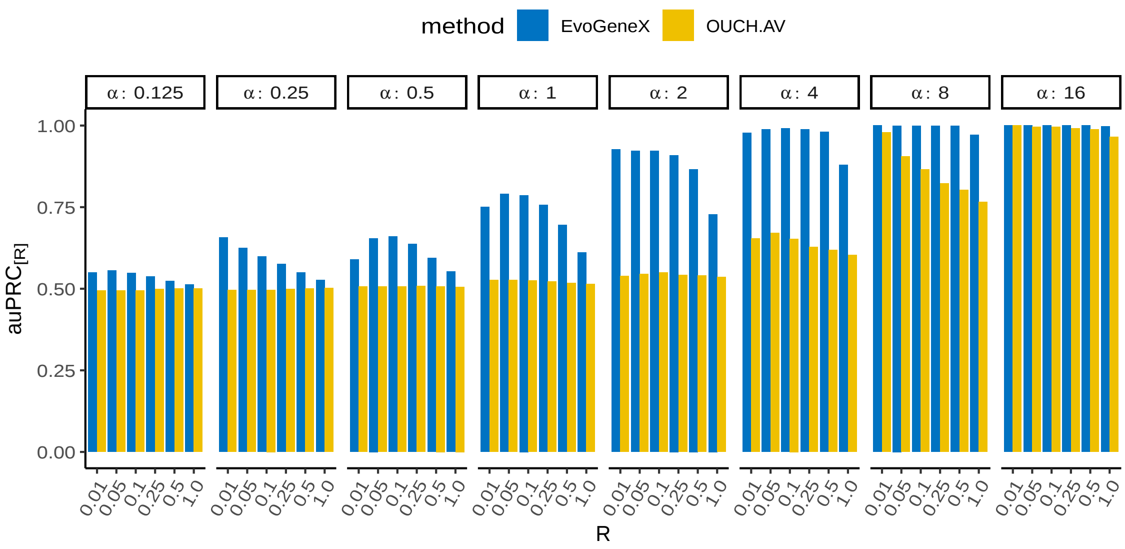

Figure S23: **Detailed results on single-regime simulations on *dros48* using 4 replicates.** For each α the genes simulated for all values of $\sigma^2 \in \{1, 2, 5, 10, 20, 50, 100\}$ and $\gamma \in \{1, 1/2, 1/4, 1/8, 1/16, 1/32, 1/64\}$ under the condition $\alpha\gamma \leq 0.5$ representing the cases where within-species variation does not exceed the between-species variation are aggregated while computing auPRC. As seen in Figures S21, S22 and S23, unless α is too high, increasing number of species while keeping α and branch length fixed, EvoGeneX performs better by utilizing replicated expression values.

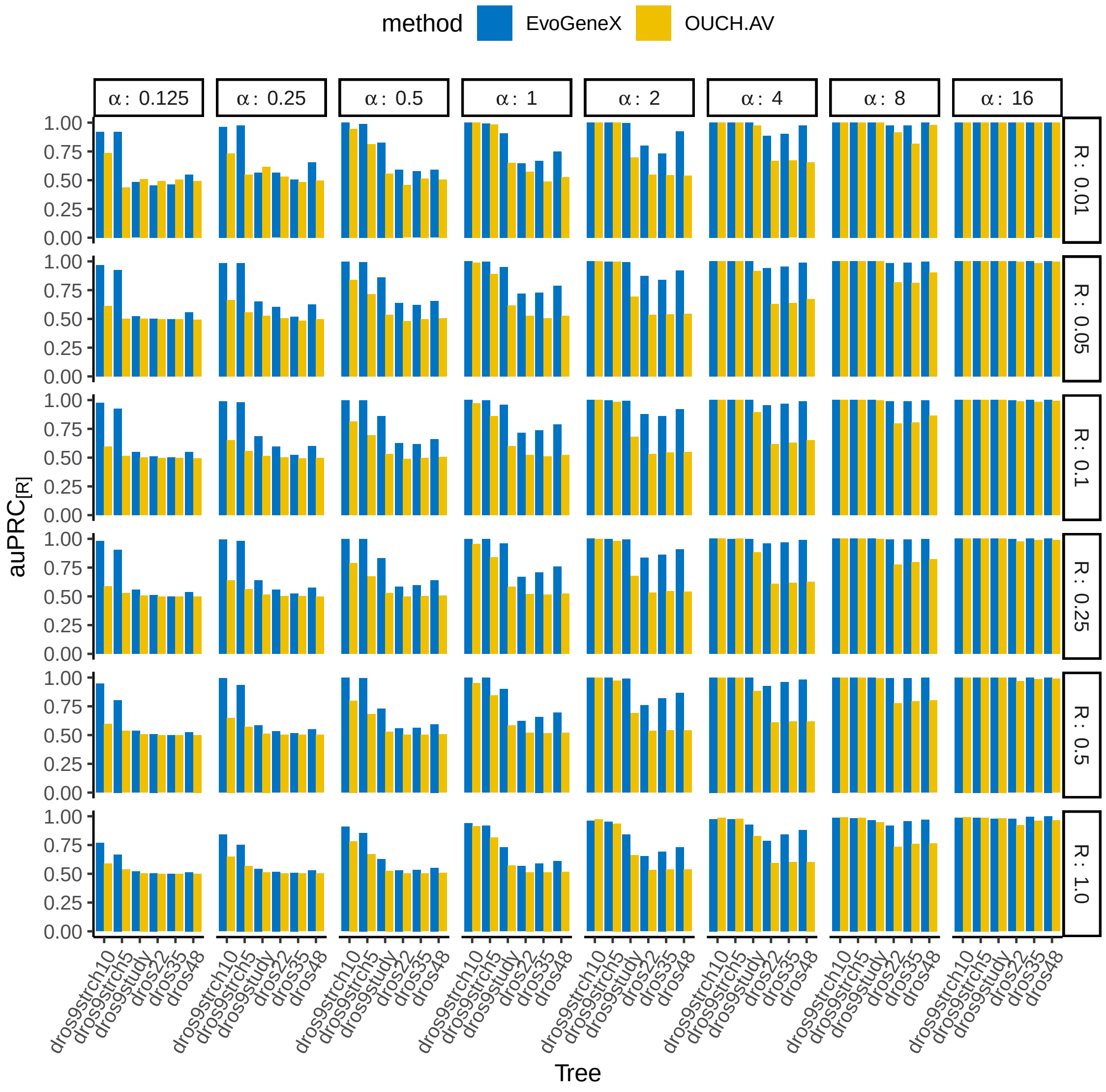

Figure S24: **Comparing single-regime simulation results across trees using 4 replicates.** This figure combines Figures S19, S20, S18, S21, S22 and S23.

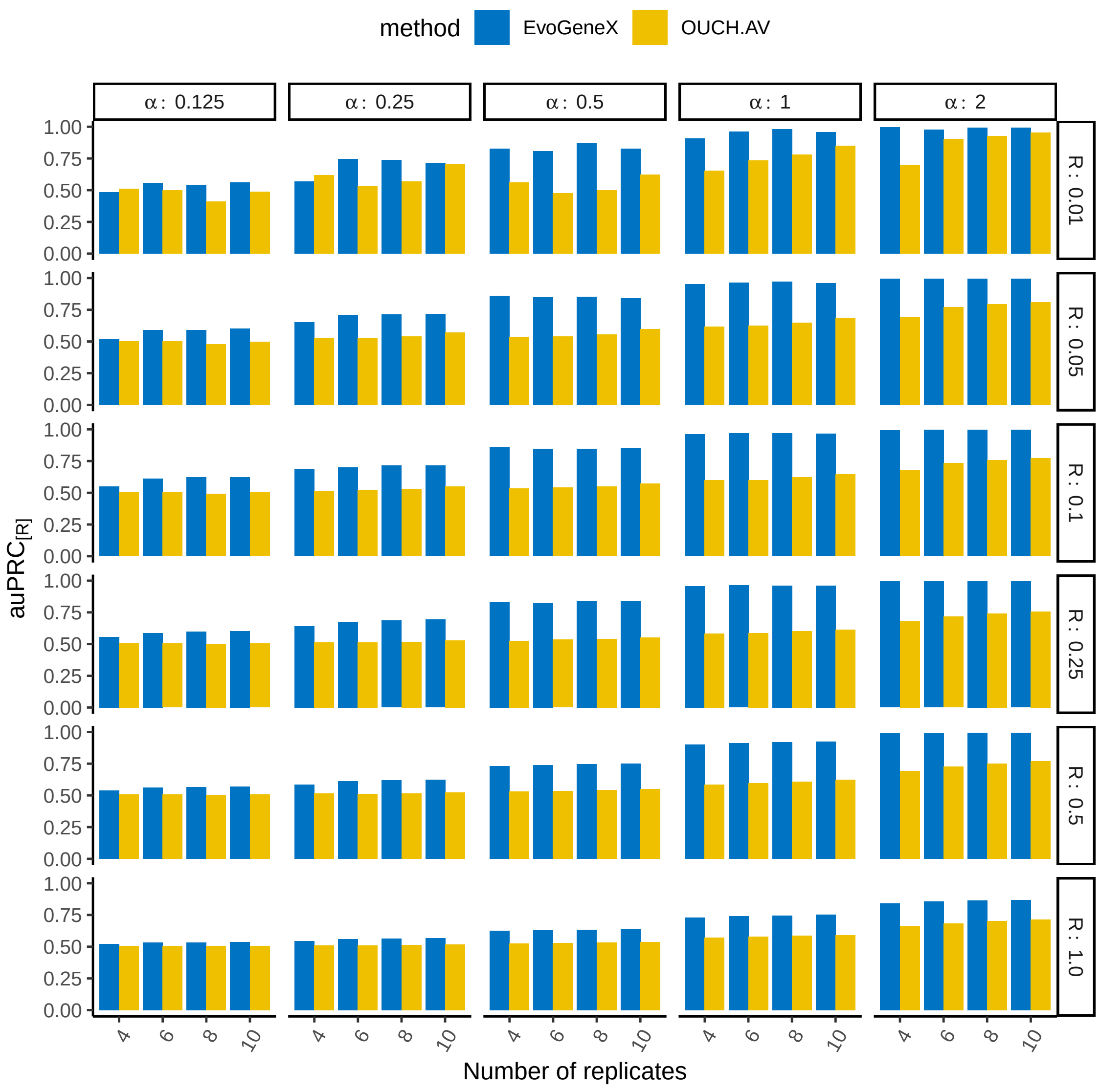

Figure S25: **Comparing single-regime simulation results across replicates on *dros9study*.** For each α the genes simulated for all values of $\sigma^2 \in \{1, 2, 5, 10, 20, 50, 100\}$ and $\gamma \in \{1, 1/2, 1/4, 1/8, 1/16, 1/32, 1/64\}$ under the condition $\alpha\gamma \leq 0.5$ representing the cases where within-species variation does not exceed the between-species variation are aggregated while computing auPRC. As number the number of replicates increases both the methods work better by incorporating more replicated values. However, EvoGeneX achieves better performance even with fewer replicates indicating its advantage in real experiments.

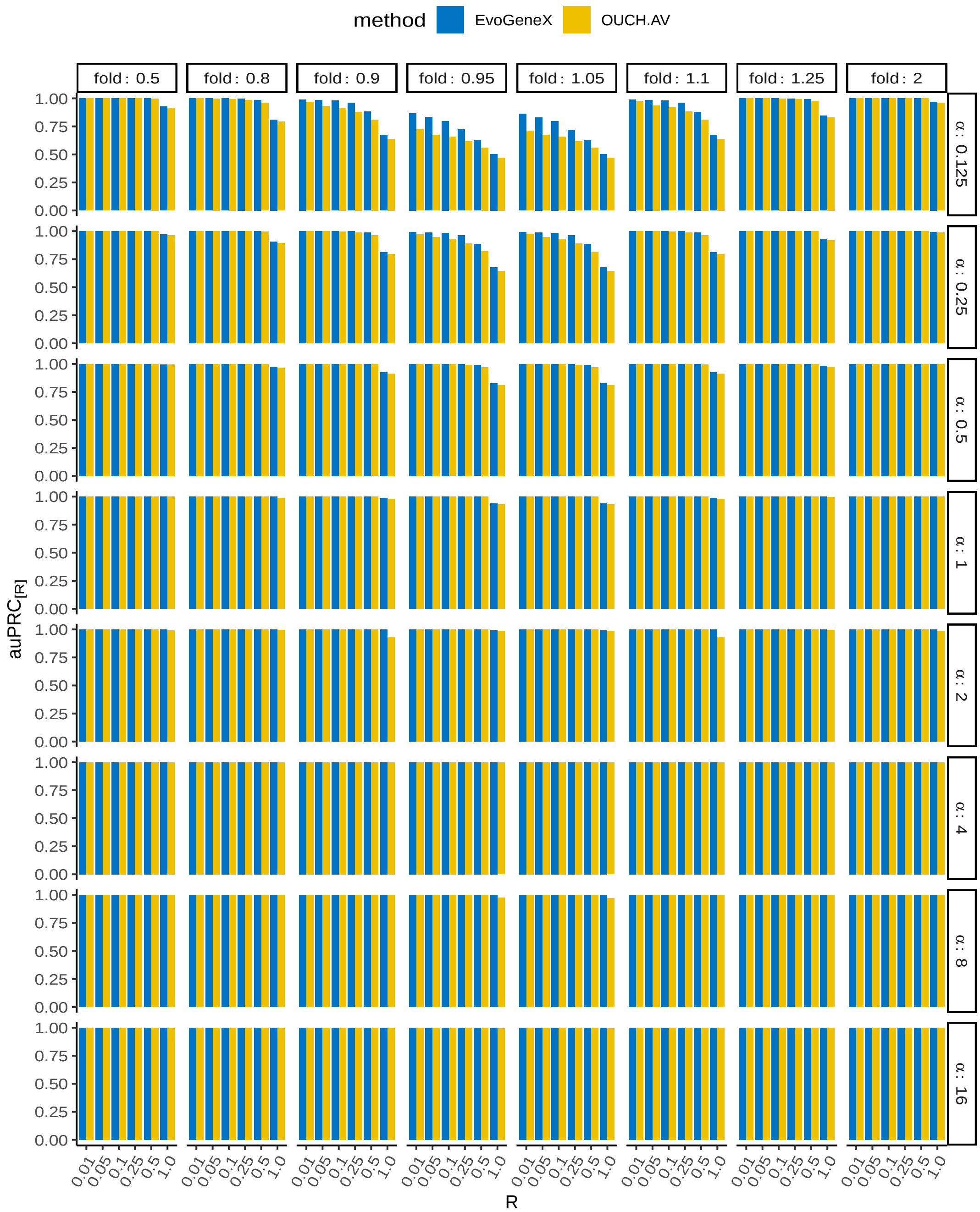

Figure S26: **Detailed results on two-regime simulations in *dros9study* using 4 replicates.** For each α the genes simulated for all values of $\sigma^2 \in \{1, 2, 5, 10, 20, 50, 100\}$ and $\gamma \in \{1, 1/2, 1/4, 1/8, 1/16, 1/32, 1/64\}$ under the condition $\alpha\gamma \leq 0.5$ representing the cases where within-species variation does not exceed the between-species variation are aggregated while computing auPRC. When α is bigger and the two mean levels are far apart (i.e. $fold$ is too high or too low), it becomes easier for both the methods to differentiate adaptive evolution from constrained and neutral evolution and they perform similarly. For lower α and when there is not much difference between the two mean levels, EvoGeneX performs better by utilizing replicated values. As seen in Figures S26 and S27, when branch-length is bigger, then also it becomes easier for both the methods to differentiate adaptive evolution from constrained and neutral evolution and they perform similarly.

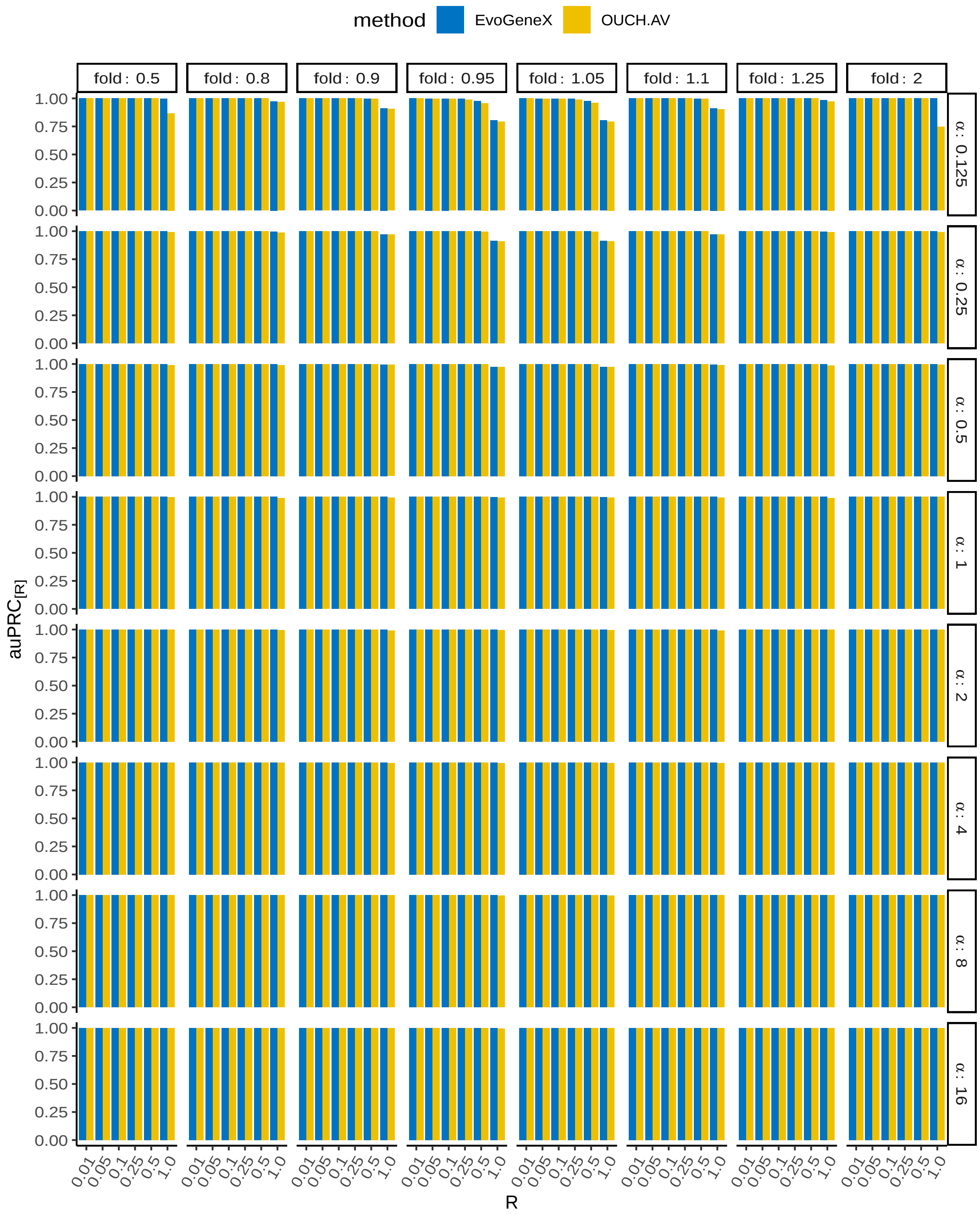

Figure S27: **Detailed results on two-regime simulations in *dros9strch10* using 4 replicates.** For each α the genes simulated for all values of $\sigma^2 \in \{1, 2, 5, 10, 20, 50, 100\}$ and $\gamma \in \{1, 1/2, 1/4, 1/8, 1/16, 1/32, 1/64\}$ under the condition $\alpha\gamma \leq 0.5$ representing the cases where within-species variation does not exceed the between-species variation are aggregated while computing auPRC. When α is bigger and the two mean levels are far apart (i.e. $fold$ is too high or low), it becomes easier for both the methods to differentiate adaptive evolution from constrained and neutral evolution and they perform similarly. As seen in Figures S26 and S27, when branch-length is bigger, then also it becomes easier for both the methods to differentiate adaptive evolution from constrained and neutral evolution and they perform similarly.

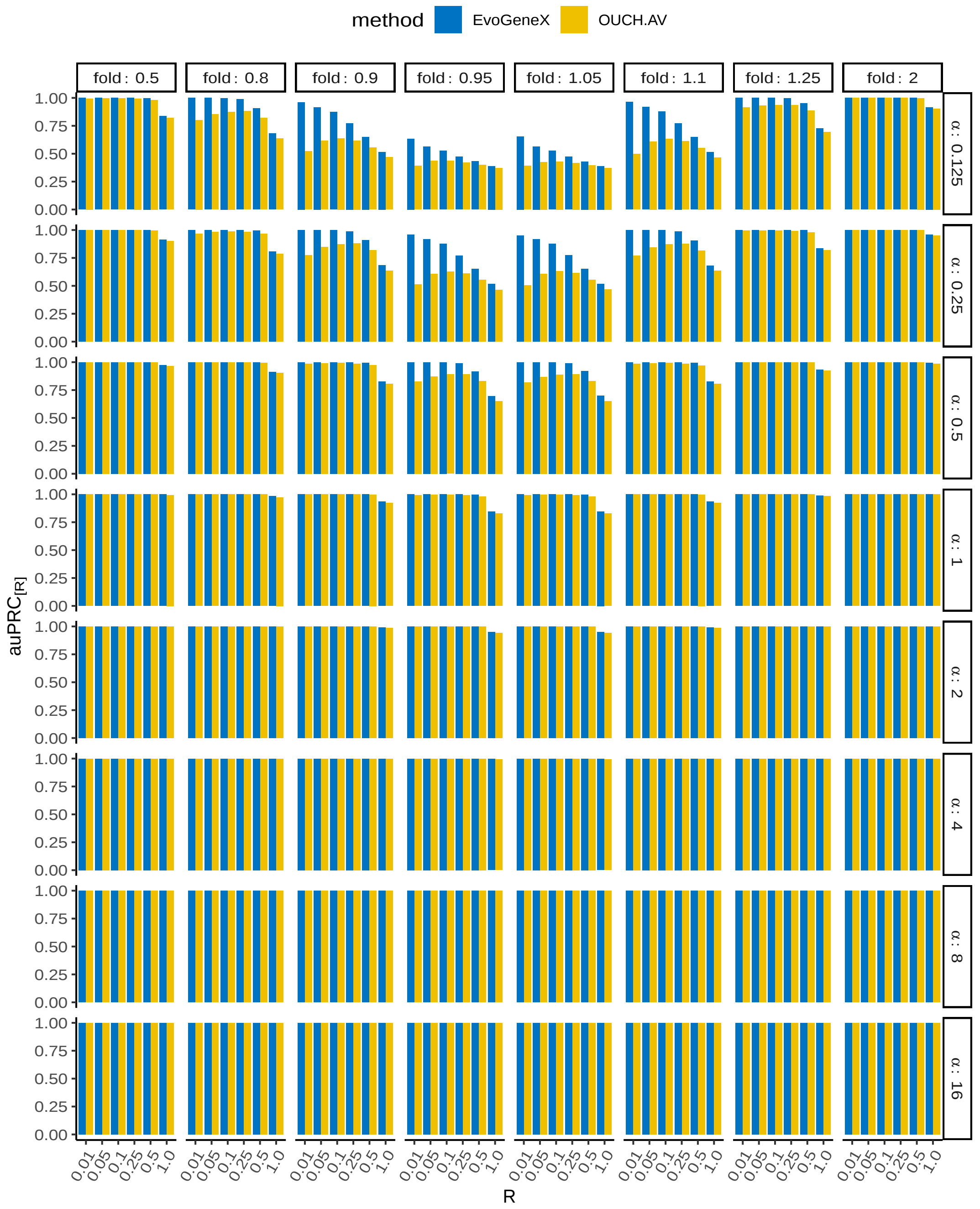

Figure S28: **Detailed results on two-regime simulations in *dros22* using 4 replicates.** For each α the genes simulated for all values of $\sigma^2 \in \{1, 2, 5, 10, 20, 50, 100\}$ and $\gamma \in \{1, 1/2, 1/4, 1/8, 1/16, 1/32, 1/64\}$ under the condition $\alpha\gamma \leq 0.5$ representing the cases where within-species variation does not exceed the between-species variation are aggregated while computing auPRC. When α is bigger and the two mean levels are far apart (i.e. $fold$ is too high or too low), it becomes easier for both the methods to differentiate adaptive evolution from constrained and neutral evolution and they perform similarly. For lower α and when there is not much difference between the two mean levels, EvoGeneX performs better by utilizing replicated values. The region of α and $fold$ where EvoGeneX performs better becomes bigger when the number of species in the tree increases as seen in Figures S28, S29 and S30.

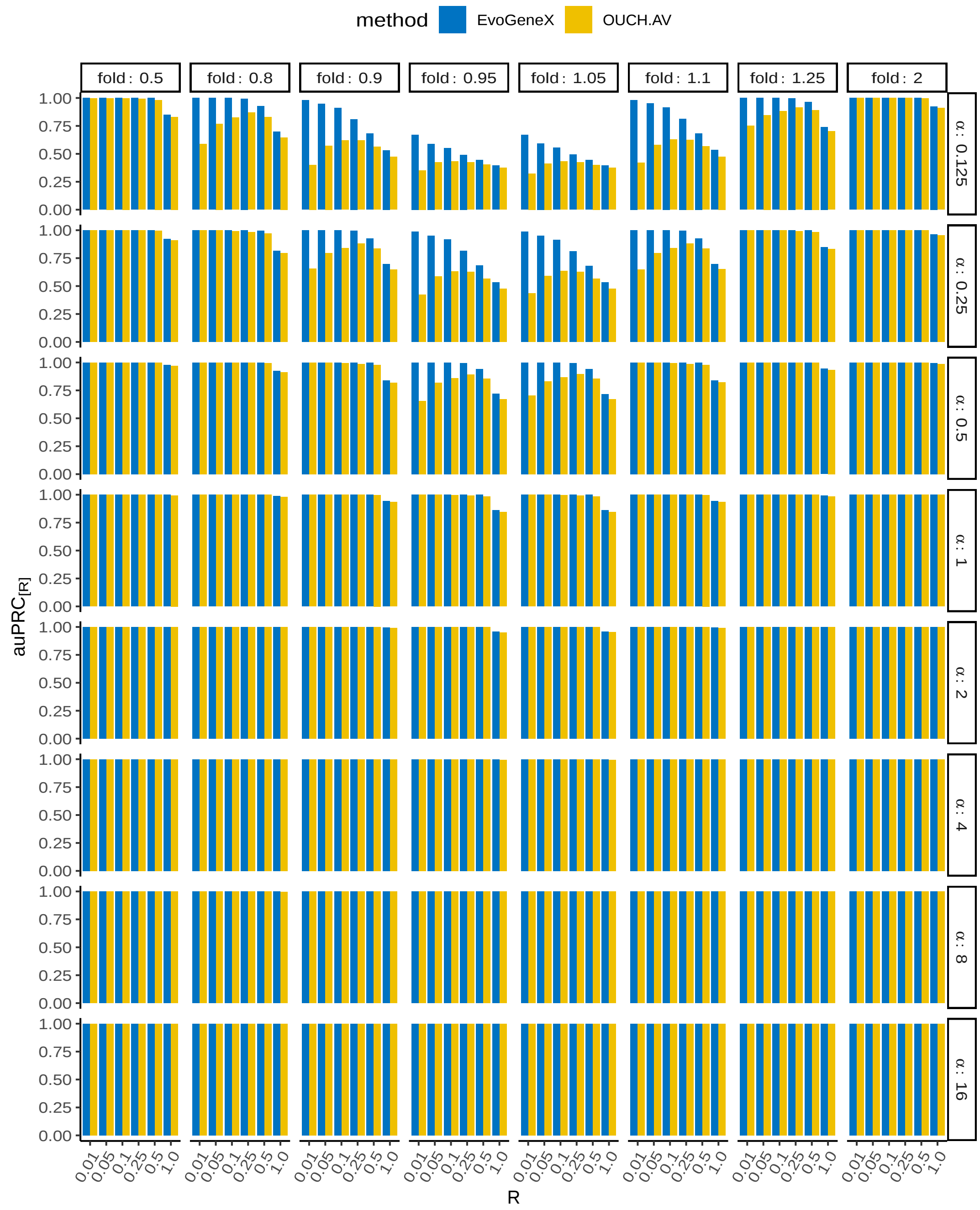

Figure S29: **Detailed results on two-regime simulations in *dros35* using 4 replicates.** For each α the genes simulated for all values of $\sigma^2 \in \{1, 2, 5, 10, 20, 50, 100\}$ and $\gamma \in \{1, 1/2, 1/4, 1/8, 1/16, 1/32, 1/64\}$ under the condition $\alpha\gamma \leq 0.5$ representing the cases where within-species variation does not exceed the between-species variation are aggregated while computing auPRC. When α is bigger and the two mean levels are far apart (i.e. $fold$ is too high or too low), it becomes easier for both the methods to differentiate adaptive evolution from constrained and neutral evolution and they perform similarly. For lower α and when there is not much difference between the two mean levels, EvoGeneX performs better by utilizing replicated values. The region of α and $fold$ where EvoGeneX performs better becomes bigger when the number of species in the tree increases as seen in Figures S28, S29 and S30.

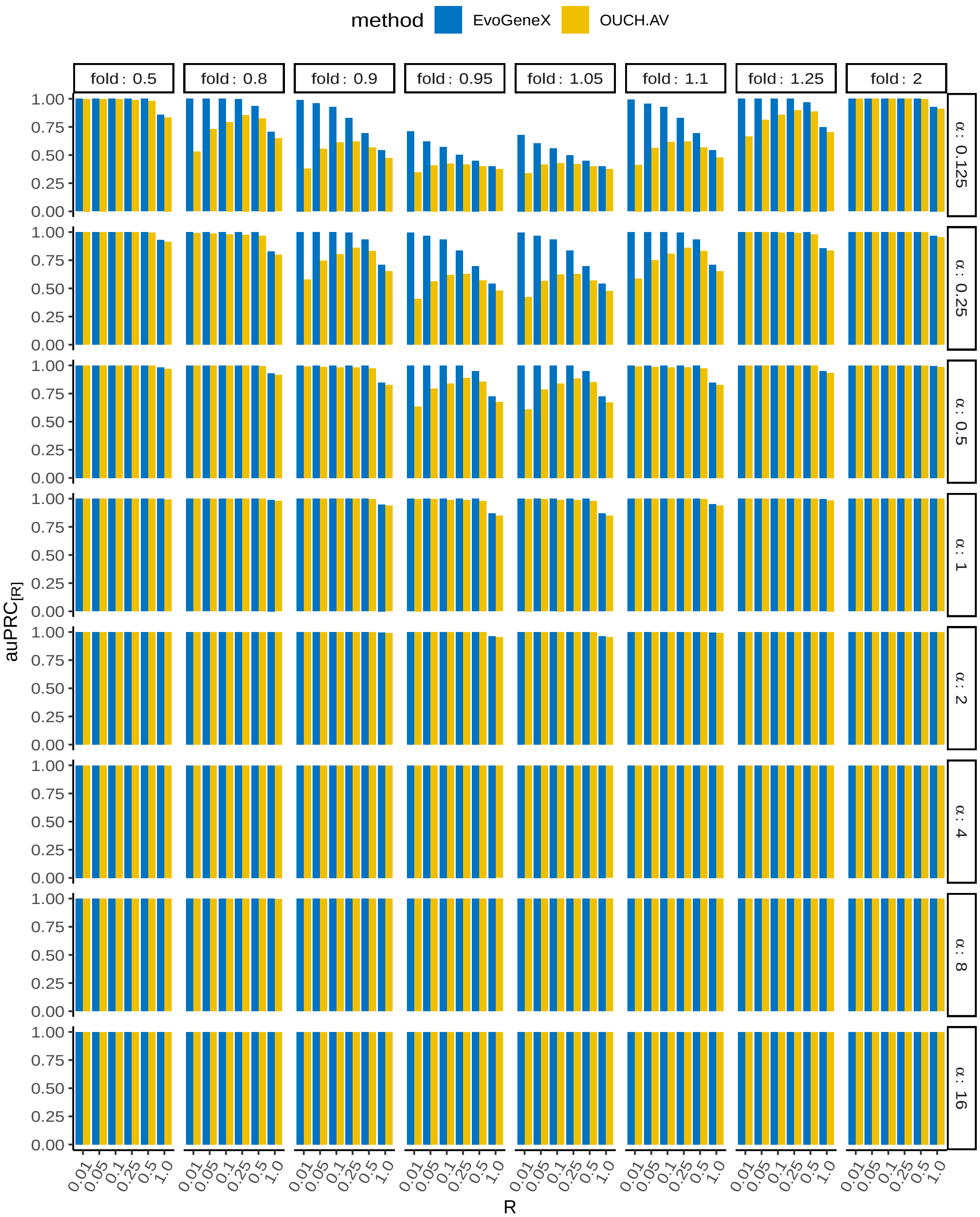

Figure S30: **Detailed results on two-regime simulations in *dros48* using 4 replicates.** For each α the genes simulated for all values of $\sigma^2 \in \{1, 2, 5, 10, 20, 50, 100\}$ and $\gamma \in \{1, 1/2, 1/4, 1/8, 1/16, 1/32, 1/64\}$ under the condition $\alpha\gamma \leq 0.5$ representing the cases where within-species variation does not exceed the between-species variation are aggregated while computing auPRC. When α is bigger and the two mean levels are far apart (i.e. $fold$ is too high or too low), it becomes easier for both the methods to differentiate adaptive evolution from constrained and neutral evolution and they perform similarly. For lower α and when there is not much difference between the two mean levels, EvoGeneX performs better by utilizing replicated values. The region of α and $fold$ where EvoGeneX performs better becomes bigger when the number of species in the tree increases as seen in Figures S28, S29 and S30.

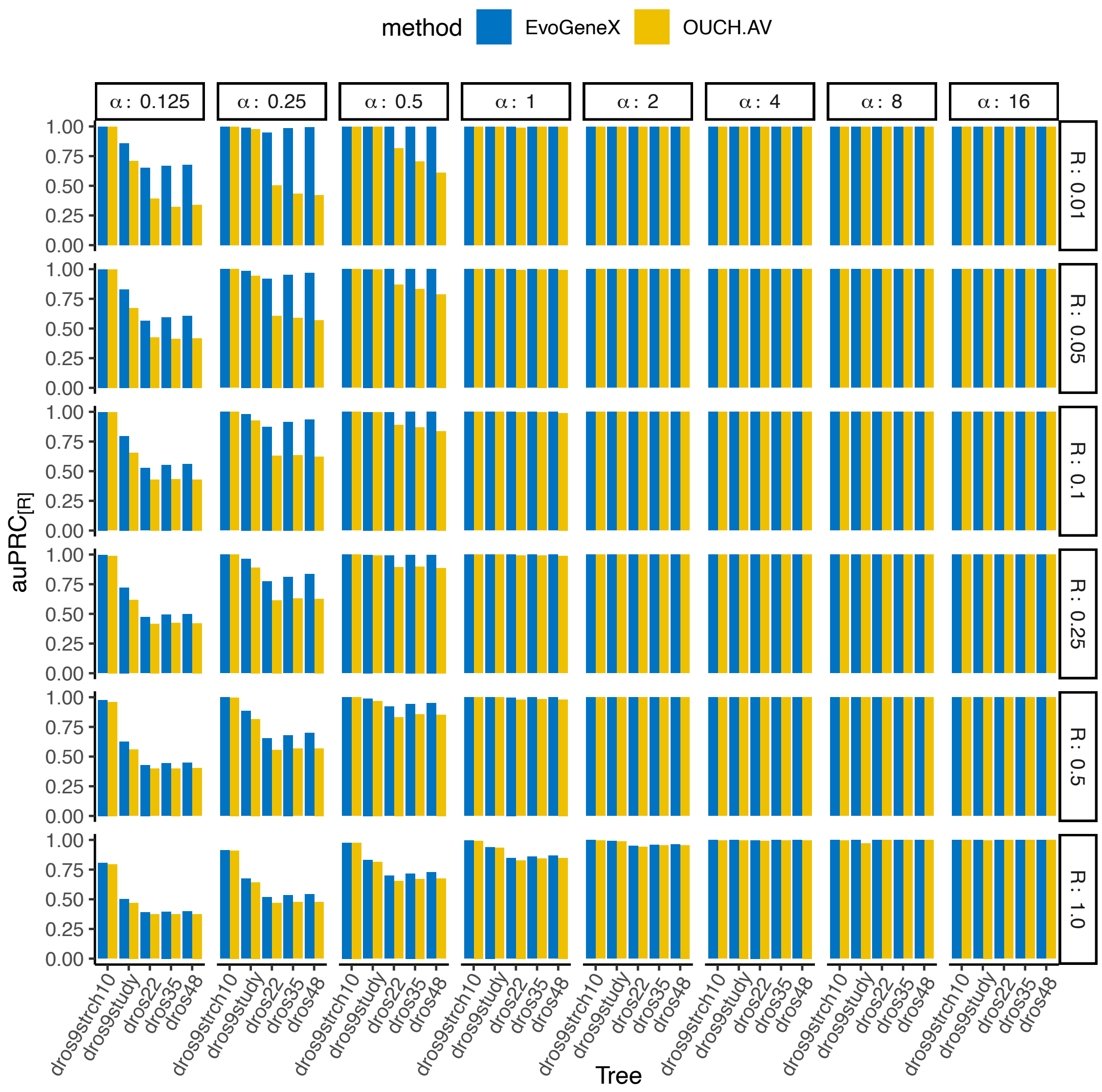

Figure S31: **Comparing two-regime simulation results across trees using 4 replicates.** This figures combines the results for $fold = 1.05$ in Figures S27, S28, S29 and S30. Note that we intentionally skipped *dros9strch5* as the results on it is expected to be somewhat in between the results on *dros9strch10* and *dros9study*.

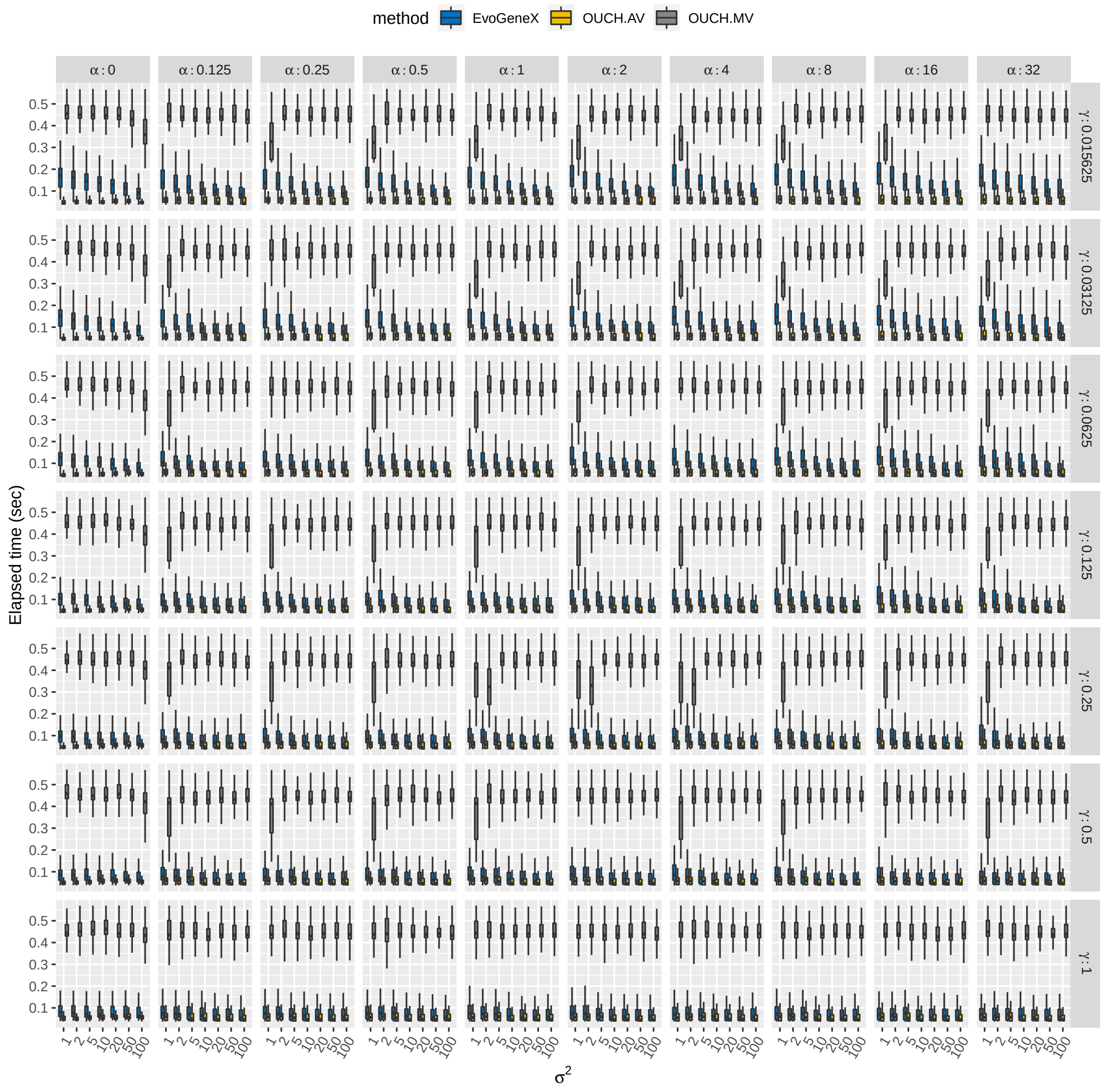

Figure S32: **Box plots of time taken in seconds by OUCH (both AV and MV versions) and EvoGeneX per gene on an all single-regime simulations on *dros9study* using 4 replicates.** Though the time taken by EvoGeneX is slightly higher than OUCH.AV, it takes far less time than OUCH.MV in all cased. Noting that the covariance matrix used by EvoGeneX is $O(K^2)$ bigger than that used by OUCH.AV, if we normalize the time taken (not shown here) by dividing it by the K^2 where K is the number of replicates (here 4), in many cases EvoGeneX does better than OUCH.AV.

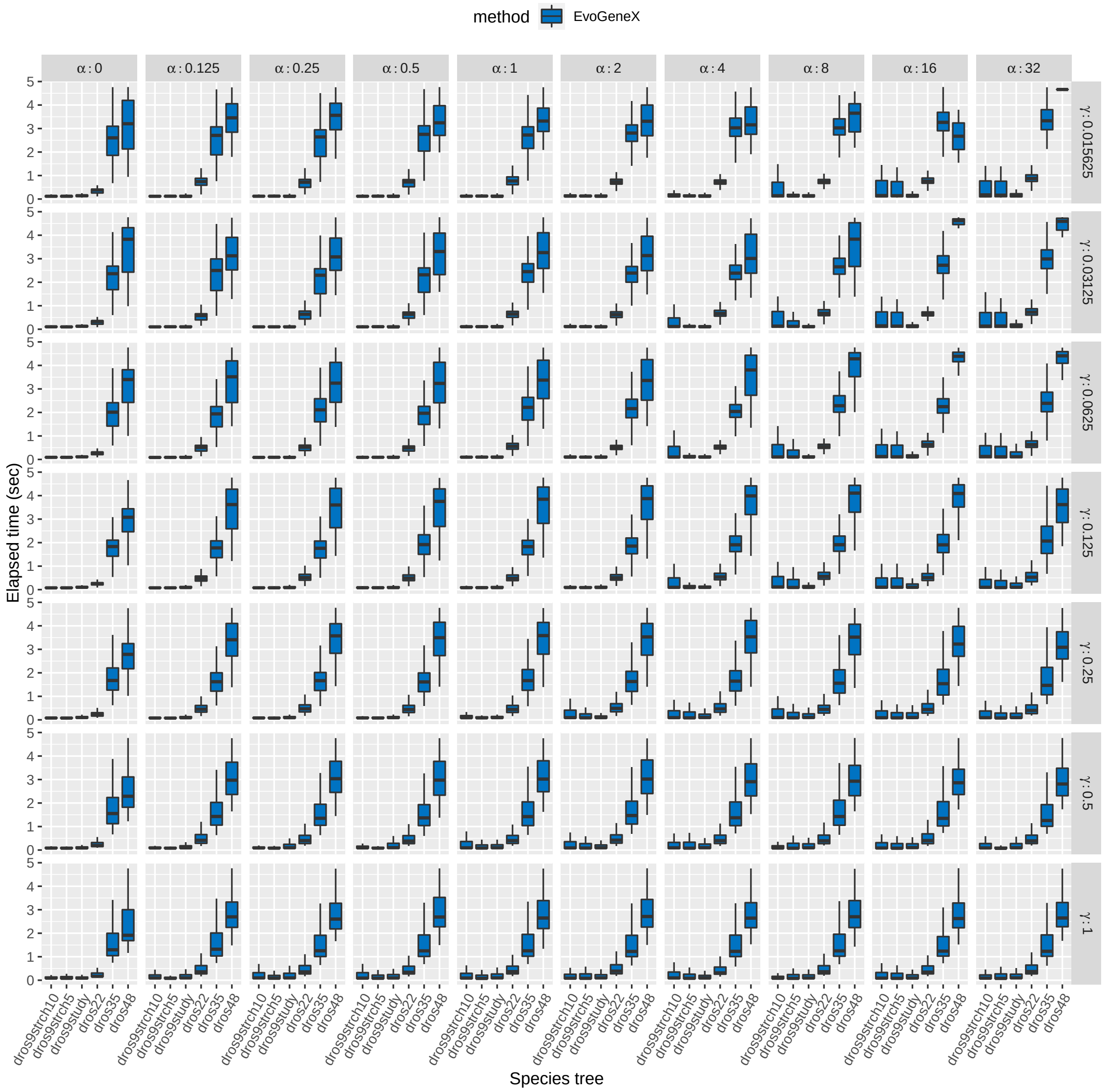

Figure S33: **Time taken in seconds by EvoGeneX on an all single-regime simulations across trees using 4 replicates.** EvoGeneX scales well with increasing number of species.

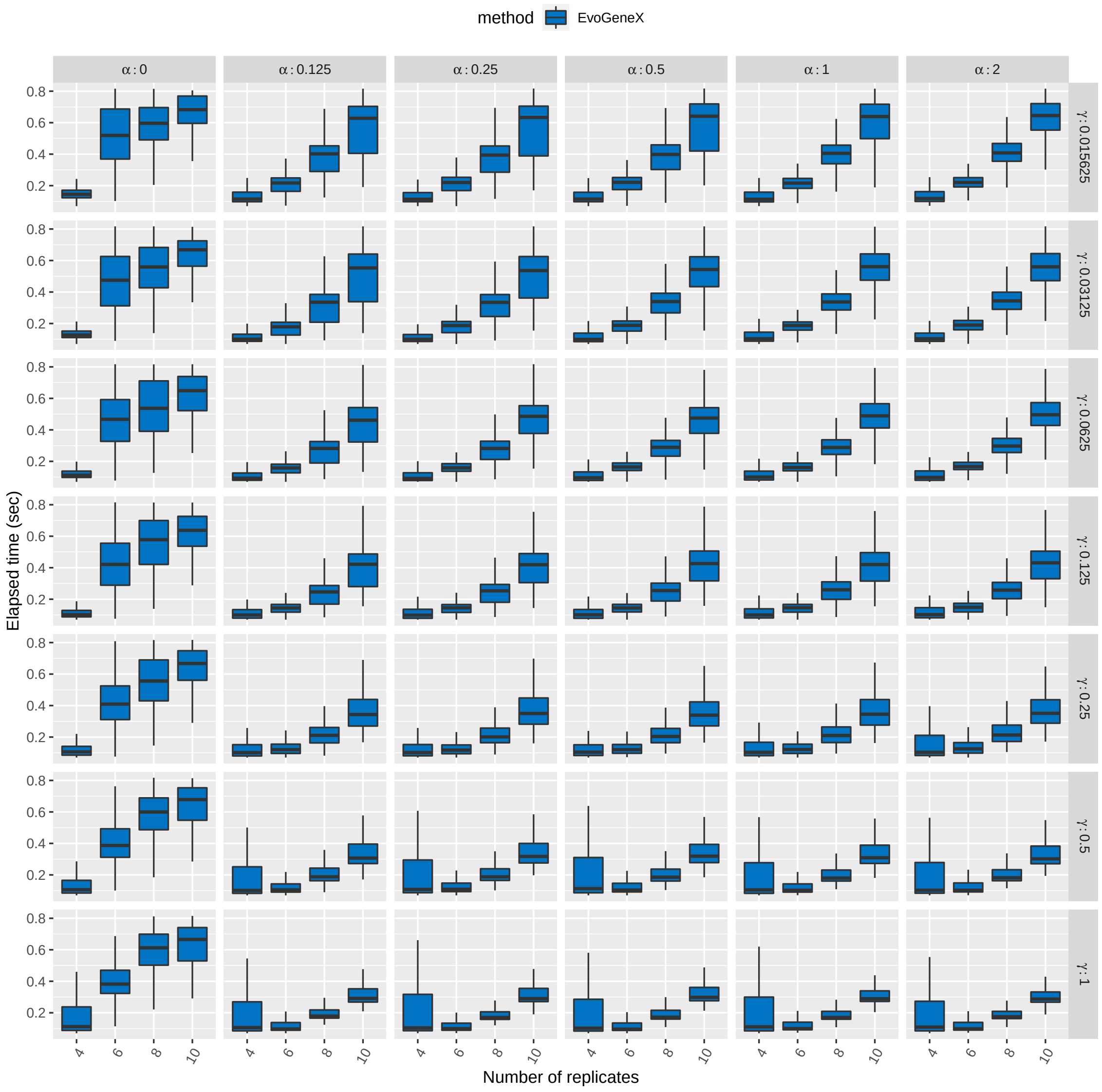

Figure S34: **Time taken in seconds by EvoGeneX on an all single-regime simulations on *dros9study* across total number of replicates used.** EvoGeneX scales well with increasing number of replicates used.

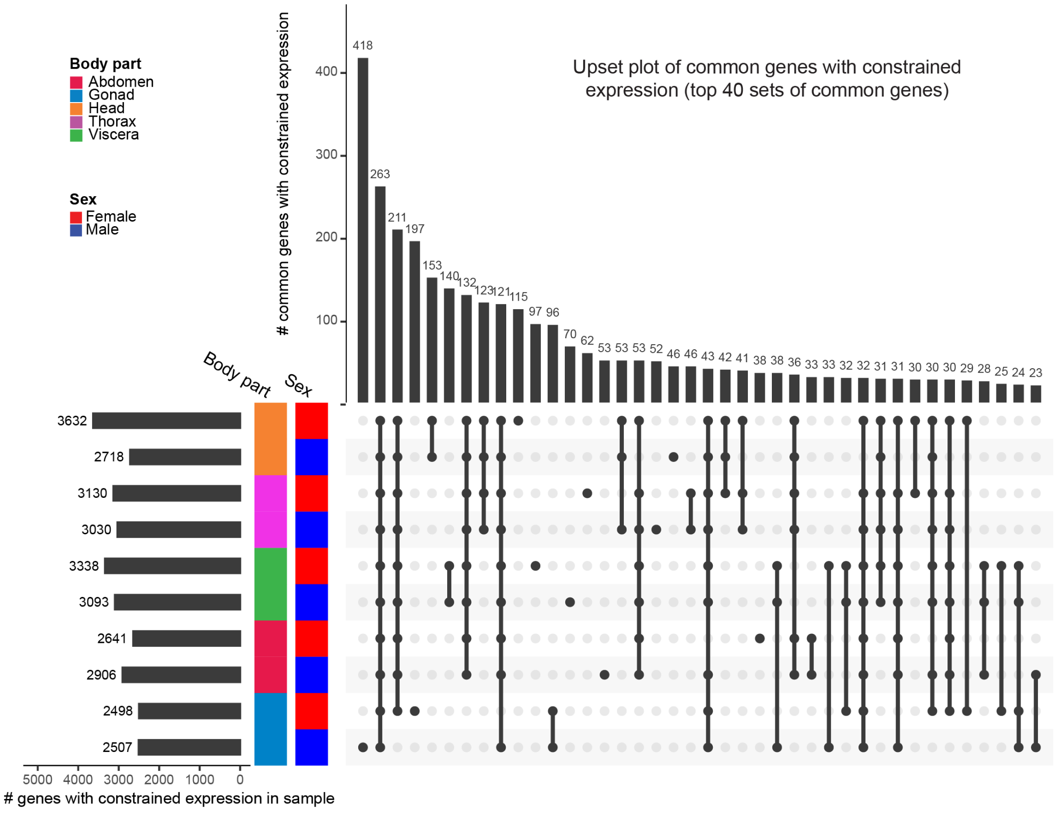

Figure S35: **Overlap of constrained gene groups across tissues and sexes**

S6 Overlap of constrained genes

Figure S35 extends Figure 3e by showing all overlaps of sets of constrained genes.

S7 Expression divergence of gene groups

Figure S37 shows an extended version of Figure 3f demonstrating that the results do not depend on the reference species. Here, two additional species, *D. pseudoobscura* and *D. virilis* were used as reference species from which rest of the species expression divergence and evolutionary time were computed.

Table S3 extends Table 2 utilizing species *D. pseudoobscura* and *D. virilis*, as reference species (in addition to *D. melanogaster*), for computing expression divergence across time. Additionally, another set of Michaelis-Menten curves were fitted, corresponding to the rows labelled as ref = all, considering all points together irrespective of the species used as reference. This demonstrates that the results are not dependent on the reference species.

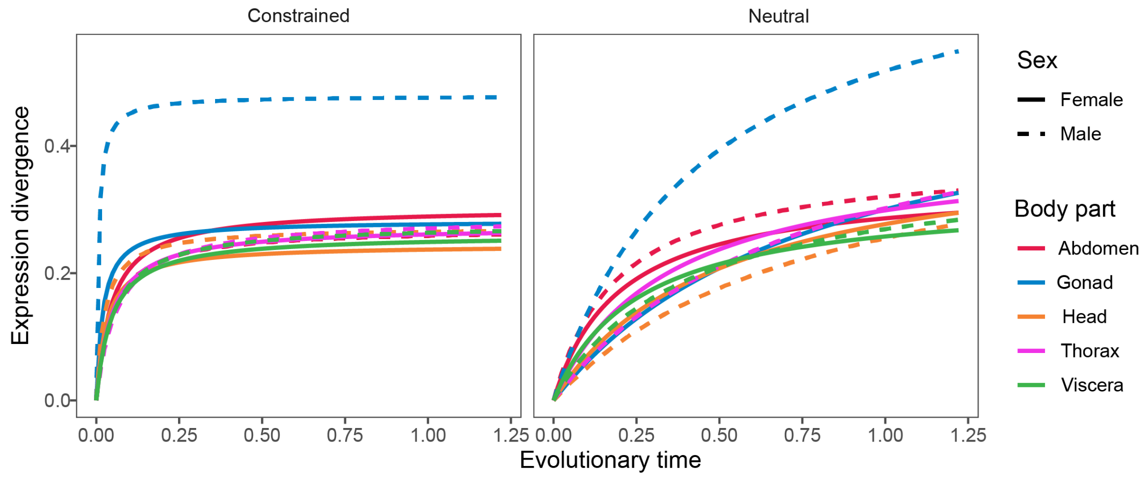

Figure S36: **Michaelis-Menten curves fitted to expression divergence of all species in reference to *D. melanogaster* in all body-parts and sexes.**

Table S3: **Michaelis-Menten curve parameters fitted to expression divergence of different species with respect to three different species (*D. melanogaster*, *D. pseudoobscura*, *D. virilis*) as reference.** The reference 'All' corresponds to case when the divergence points for the three different reference are collectively fitted to a Michaelis-Menten curve.

		V_{max}				K			
	**Genes**	Constrained		Neutral		Constrained		Neutral	
	**Sex**	Female	Male	Female	Male	Female	Male	Female	Male
**Ref.**	**Body part**								
	**Head**	0.221	0.244	0.336	0.330	1.211e-04	4.931e-04	2.472e-01	3.169e-01
	**Thorax**	0.251	0.250	0.323	0.350	1.745e-04	2.826e-03	1.260e-01	1.971e-01
All	**Viscera**	0.227	0.241	0.305	0.330	1.584e-03	3.854e-03	1.599e-01	2.034e-01
	**Abdomen**	0.273	0.253	0.324	0.384	3.074e-04	1.769e-03	1.253e-01	1.356e-01
	**Gonad**	0.266	0.469	0.398	0.634	1.000e-04	9.559e-03	3.360e-01	2.547e-01
	**Head**	0.245	0.272	0.417	0.459	3.174e-02	2.506e-02	5.041e-01	8.012e-01
	**Thorax**	0.274	0.288	0.403	0.532	4.843e-02	6.337e-02	3.486e-01	7.621e-01
Dmel	**Viscera**	0.261	0.278	0.323	0.377	4.665e-02	5.348e-02	2.537e-01	3.996e-01
	**Abdomen**	0.303	0.271	0.343	0.382	4.647e-02	4.613e-02	1.996e-01	1.927e-01
	**Gonad**	0.282	0.479	0.540	0.755	2.022e-02	6.266e-03	7.974e-01	4.573e-01
	**Head**	0.219	0.241	0.536	0.648	1.000e-04	2.109e-04	9.640e-01	1.494e+00
	**Thorax**	0.252	0.248	0.784	1.555	1.000e-04	2.162e-03	1.631e+00	3.918e+00
Dpse	**Viscera**	0.224	0.240	0.563	0.869	1.065e-03	3.106e-03	9.735e-01	1.993e+00
	**Abdomen**	0.274	0.261	0.281	0.346	1.000e-04	1.769e-03	2.966e-02	2.217e-02
	**Gonad**	0.268	0.494	0.562	0.521	1.000e-04	1.065e-02	8.309e-01	4.733e-02
	**Head**	0.223	0.243	0.293	0.277	3.879e-02	4.096e-02	8.298e-02	1.024e-01
	**Thorax**	0.246	0.239	0.288	0.288	1.000e-04	1.000e-04	1.000e-04	1.000e-04
Dvir	**Viscera**	0.219	0.252	0.273	0.299	1.076e-02	9.006e-02	4.893e-02	5.565e-02
	**Abdomen**	0.270	0.261	0.316	0.380	1.488e-02	4.177e-02	5.989e-02	9.329e-02
	**Gonad**	0.320	0.480	0.331	0.541	1.870e-01	8.910e-02	1.156e-01	1.167e-01

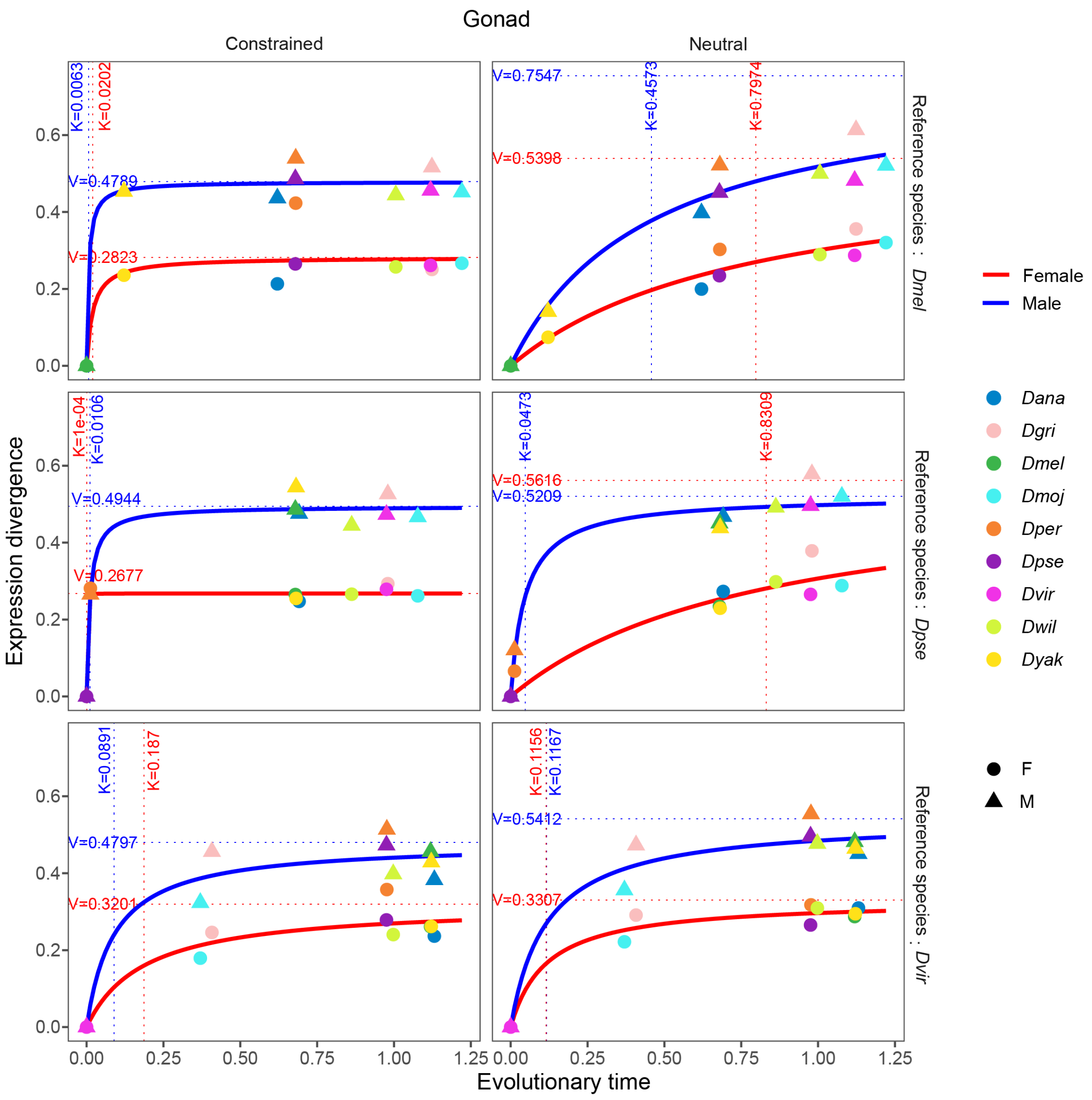

Figure S37: **Expression divergence in gonads over time with respect to three different species,** *D. melanogaster*, *D. pseudoobscura* **and** *D. virilis*, **as reference relative to which the time is calculated.**

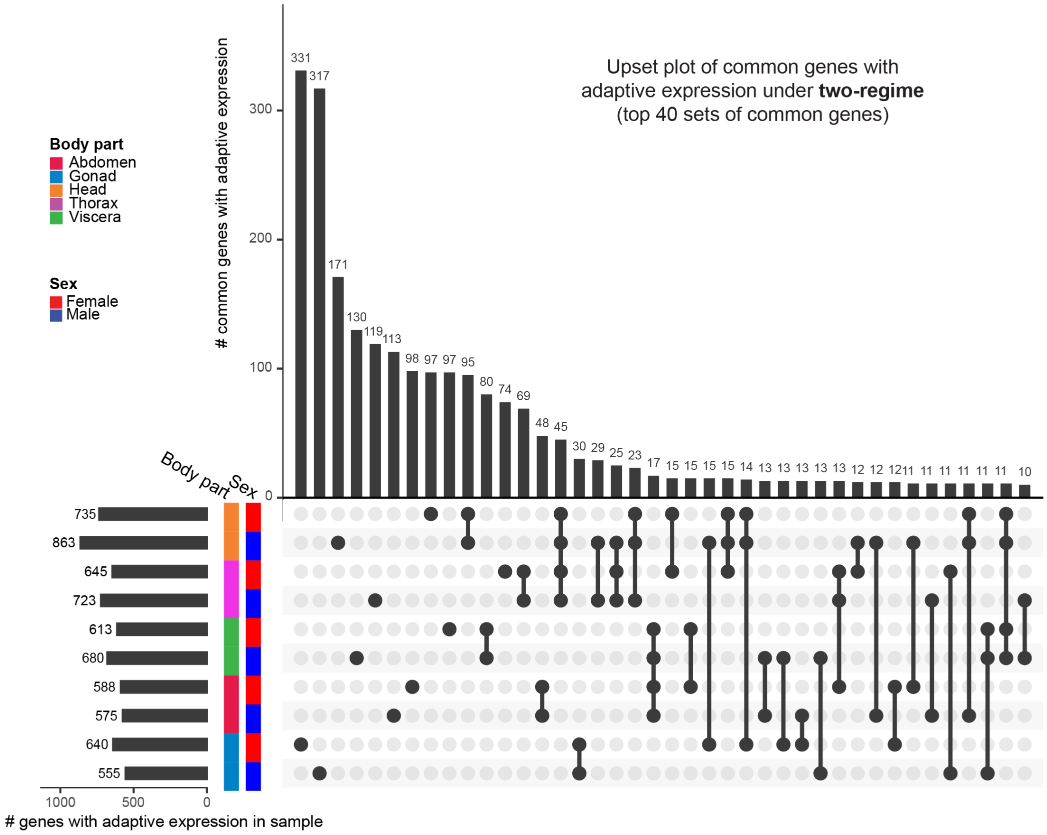

Figure S38: **UpSet plot of top 40 sets of common adaptive genes under two-regime.**

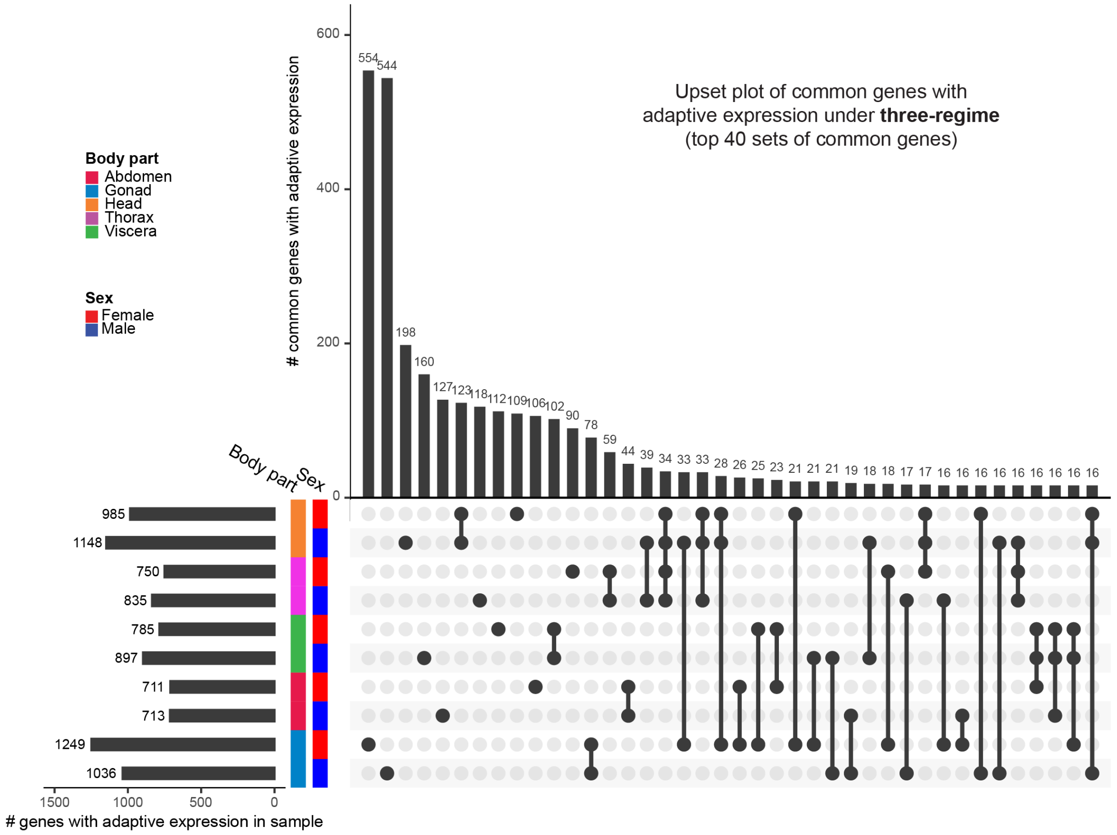

Figure S39: **UpSet plot of top 40 sets of common adaptive genes under three-regime.**

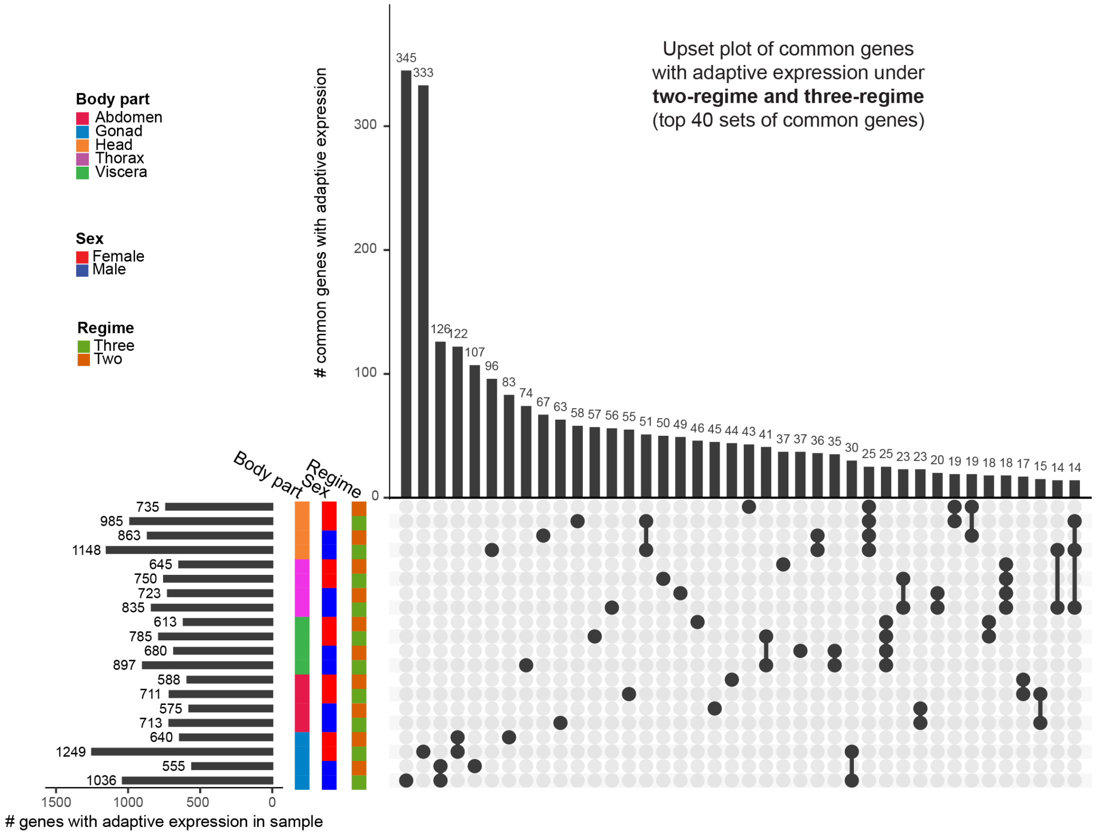

Figure S40: **UpSet plot of top 40 sets of common adaptive genes under both two-regime and three-regime.**

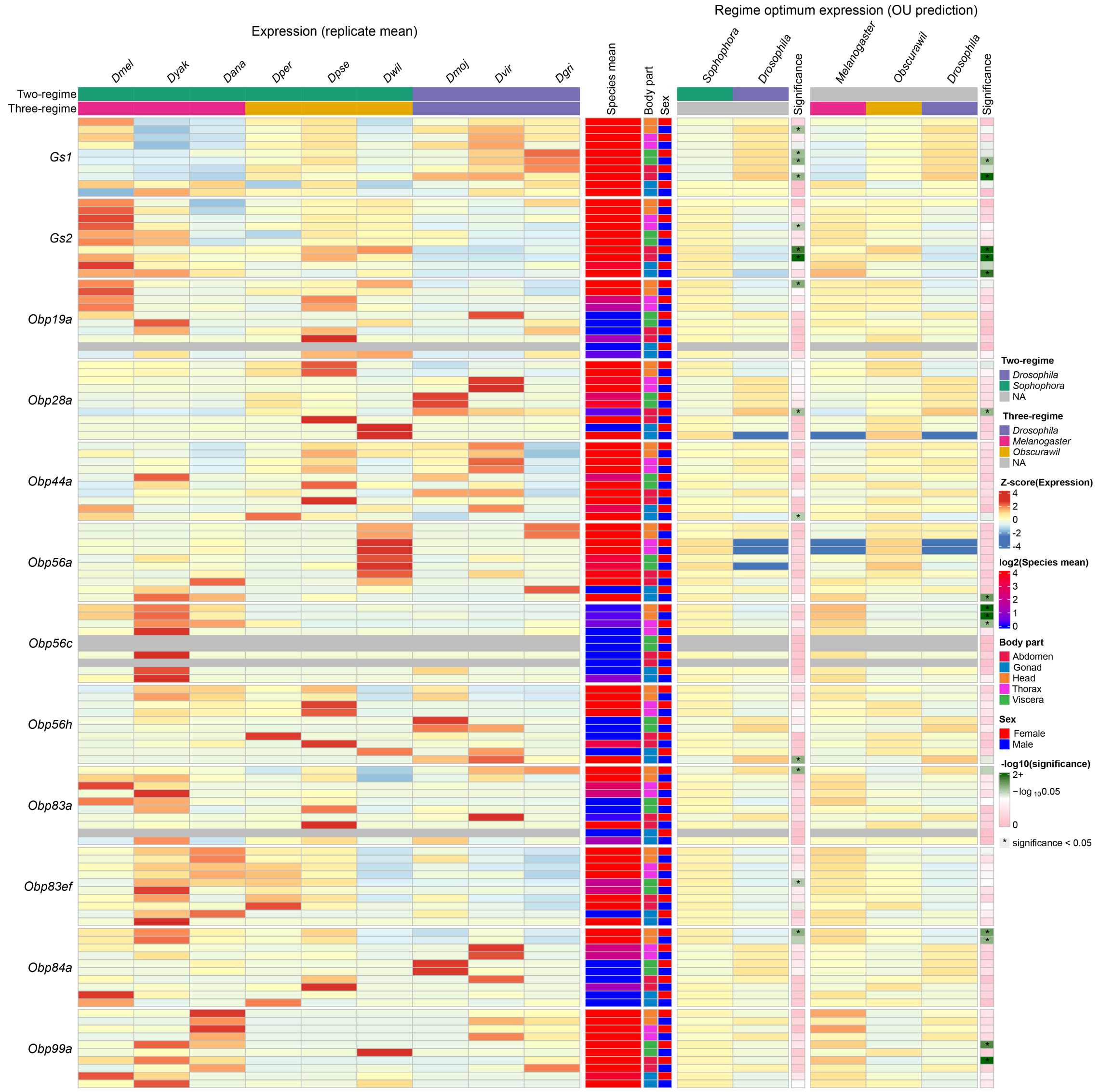

Figure S41: **Detailed view of expression evolution of example adaptive genes in all body-parts.**

References

Brawand, David et al. (Oct. 2011). "The Evolution of Gene Expression Levels in Mammalian Organs". In: *Nature* 478.7369, pp. 343–348. ISSN: 1476-4687. DOI: 10.1038/nature10532.

Chen, Jenny et al. (Jan. 2019). "A Quantitative Framework for Characterizing the Evolutionary History of Mammalian Gene Expression". In: *Genome Research* 29.1, pp. 53–63. ISSN: 1088-9051, 1549-5469. DOI: 10.1101/gr.237636.118.

Chen, Zhen-Xia et al. (July 2014). "Comparative Validation of the D. Melanogaster modENCODE Transcriptome Annotation". In: *Genome Research* 24.7, pp. 1209–1223. ISSN: 1088-9051, 1549-5469. DOI: 10.1101/gr.159384.113.

Hansen, Thomas F. (Oct. 1997). "Stabilizing Selection and the Comparative Analysis of Adaptation". In: *Evolution* 51.5, pp. 1341–1351. ISSN: 0014-3820. DOI: 10.1111/j.1558-5646.1997.tb01457.x.

Hansen, Thomas F. and Emília P. Martins (1996). "Translating Between Microevolutionary Process and Macroevolutionary Patterns: The Correlation Structure of Interspecific Data". In: *Evolution* 50.4, pp. 1404–1417. ISSN: 1558-5646. DOI: 10.1111/j.1558-5646.1996.tb03914.x.

King, Aaron A. and Marguerite A. Butler (2009). *Ouch: Ornstein-Uhlenbeck Models for Phylogenetic Comparative Hypotheses.*

Longdon, Ben (Jan. 2015). "Phylogeny of 48 Species of Drosophilidae". In: DOI: 10.6084/m9.figshare.1289750.v1.

Love, Michael I., Wolfgang Huber, and Simon Anders (Dec. 2014). "Moderated Estimation of Fold Change and Dispersion for RNA-seq Data with DESeq2". In: *Genome Biology* 15.12, p. 550. ISSN: 1474-760X. DOI: 10.1186/s13059-014-0550-8.

Wilks, S. S. (1938). "The Large-Sample Distribution of the Likelihood Ratio for Testing Composite Hypotheses". In: *The Annals of Mathematical Statistics* 9.1, pp. 60–62. ISSN: 0003-4851.

Yang, Haiwang et al. (Dec. 2018). "Re-Annotation of Eight Drosophila Genomes". In: *Life Science Alliance* 1.6, e201800156. ISSN: 2575-1077. DOI: 10.26508/lsa.201800156.
